# The Essays, Cases, Intelligence, &c.: H—R

**Published:** 1820

**Authors:** 


HADLEY, Mr. on the influenza of 1803, x. 212.

HÆMATEMESIS, case of, xiii. 239; particulars of an interesting case of, xxx. 513; attended by melæna, fatal case of, xxii. 180.

HÆMATIRRHŒA, or petechiæ without fever, fatal case of, xxx. 339.

HÆMATOCELE, or venous aneurism, observations on, xxi. 389; cases of, xxiv. 285.

HÆMOPTOE, a singular case of, iii. 424; Dr. Reid's opinion on, xv. 461; efficacy of repeated bleedings in the cure of, xviii. 320; alarming and obstinate cases of, cured by the exhibition in large and repeated doses of acetate of lead, xix. 70.—*See* Lungs.

HÆMORRHOIDS, new treatment of, iii. 334; case of a singular and favourable termination of, xxii. 488.

HAGSTROM, Dr. on chronic dysphagia arising from a ravenous appetite, iii. 366.

HAGUE, Mr. his case of remarkable recovery from a very extensive wound of the abdomen, xxi. 414.

HAHNEMANN, Dr. his test for wine, iii. 171; his discovery of a new salt, which he calls pneumalkali, iv. 446; his preservative remedy against scarlet fever, vi. 367.

HAINES, Mr. recommends Mr. Dennet's mode of preserving the vaccine virus, x. 40.

HAIR, on the dangerous effect of cutting the, during the convalescence from acute diseases, iii. 367; observations on an occasional increase and decrease of bulk in the, xxvi. 308.

HALE, Dr. on the production of animal heat, xxxii. 233; remarks on, 295.

HALFORD, Sir Henry, on the climacteric disease, xxxi. 163.

HALL, Dr. his observations on a French work on legal medicine, ix. 69; on the influenza of 1803, xiii. 209; on vaccination, xiv. 410; on hydrophobia, and on the deficiency of the prophylactic and curative means in meeting this dreadful malady, xv. 409; his observations on the use of nitric acid in icterical affections, xvii. 17; on burns and scalds, 132; on the cure of frambœsia, or yaws, xx. 401; his observations on the irritability of vegetables, 499; on cancerous tumour of the tongue, xxiv. 134; on the peculiar effects of certain odours, 223.

HALL, Dr. R. of Sedburg, his observations on the pemphigus major of Sauvages, iii. 552.

———, Mr. on vaccination, xiv. 410.

———, Mr. of Bridgenorth, on the influenza of 1803, x. 220.

———, Mr. Lodge, his observations of gonorrhœa, with the remarkable case of a female infant, xi. 452; on the screw tourniquet, xiv. 492; xv. 112.

———, Dr. M. on hydrophobia, xvii. 328.

———, Mr. J. on a peculiar disease affecting the hair of children, apparently brought from the East or West Indies, xxi. 228.

———, Dr. Marshall, his case of subcutaneous tubercle, xxxiv. 519.

HALLE, M. his description of an extraordinary fœtus, ii. 480; on the curative art, and the periods when it has made a real progress, iii. 171; his description of a remarkable case of simple idiopathic atrophy, 375; on the efficacy of animal jelly in the treatment of intermittent fevers, xii. 431.

HALLIDAY, Dr. on the influenza of 1803, x. 301; his case of epilepsy arising from worms, xix. 305.

HAMILTON, Dr. James, his letter to Mr. Blegborough on the utility of his machine for conveying a vapour-bath to diseased limbs, vii. 289; his improved saline draught, xiv. 53.

—————, Dr. James, of Edinburgh, his observations on purgative medicines, xv. 182; his observations on the utility and administration of purgative medicines in several diseases, xix. 470; his opinions on hydrocephalus, xii. 318, 390.

—————, Dr. of Halesworth, on digitalis, with cases of consumption and hydrothorax, xvi. 229, 358.

—————, the late Dr. William, his work on hydrophobia, xxi. 272; his opinion of the efficacy of digitalis in some kinds of dropsy, particularly in hydrothorax, 331.

—————, Dr. R. on the effects of ipecacuan, xxiii. 318.

—————, Mr. W. on puerperal fever, xxiv. 107; his case of gravel, 267; on the Walcheren fever, xxv. 1; on scarlatina, 101, 406; on the theory of worms, xxvi. 276; on dissolving gum elastic, 382; on a peculiarity in vaccination, xxviii. 452; on the efficacy of calomel in cases of obstinate vomiting, xxx. 100.

HAMM, Dr. his case of the cure of hydrocephalus by copious depletion, xix. 217.

HAMMICK, Mr. his case of gun-shot wound, xxvii. 265.

HANGING, cases of suspended animation by, with a new and easy process for recovery from, xvi. 529.

HANOVER, particulars of vaccine inoculation in, iii. 471.

HANSARD, Mr. on the influenza of 1803, x. 97.

HANSLOPE, Mr. proposes to substitute the term "*deliverer*" for what he calls the present hermaphrodite expression of man-midwife, xiii. 190.

HARDMAN, Mr. his case demonstrative of the advantage of bringing on premature labour in a distorted patient, v. 253; on two cases in which the Cesarean operation was unsuccessfully performed, 353; on the most effectual mode of opening abscesses, xi. 327; on hastening the delivery in cases of distorted pelvis, 496.

HARDWICK, Mr. his observations on hydrophobia, with the particulars of a case which terminated fatally, xxi. 23.

HARDWICKE, Lord, his letter to Mr. Angerstein, ix. 540; answer to, signed by Dr. Jenner and Mr. Ring, 541.

HARE, Mr. on apoplexy, vii. 397.

HARE-LIP, new method of operating for the, xi. 193; observations on the, xxv. 165.

HARES exhibit the musk-odour in the rutting-season, vi. 342.

HARGENS, Dr. on the utility of alkali in convulsive affections, iv. 183.

HARGROVE, Mr. his case of a solution of continuity in the parietal bones, not consequent to internal injury, xxiii. 373; on the inability to produce the effect of emetics in horses, xxiv. 271.

HARLES, Professor, on the efficacy of the oleum hyoscyami in hæmoptysis, iii. 576; his relation of a remarkable case of morbus maculosus hæmorrhagicus, accompanied by dropsy, v. 489.

HARLESTONENSIS, on the rage for novelty which pervades medical society, and on the failure of mercury, so strongly recommended by some in typhus, xix. 315; reply to, 454; his answer, 522; Veritas in answer to, xx. 132; animadversions on the observations of, by Mr. Strowger, 145; his answer to Veritas and Mr. Strowger, 266; Dr. Mossmann in reply to, more particularly as to his assertion that digitalis had fallen into general disrepute, 328.

HARMATTAN, a wind prevailing in Africa, its influence on epidemics, xix. 115.

HARNESS, Dr. on the application of gastric juice to sores, iv. 167.

———, Mr. of Tavistock, on the influenza of 1803, with a postscript from D. Geddes, esq. x. 291.

HARRINGTON, Dr. on digitalis, and its effects in various diseases, iii. 202.

HARRIOTTS, John, esq. late resident magistrate of the Thames Police-office, his account of the efficacy of cold affusion in his own particular case of a febrile attack, xx. 31.

HARRIS, Dr. on yellow fever, ix. 25; his history of a fatal case of hydrocephalus, xix. 22.

———, Dr. of Haverfordwest, on the influenza of 1803, x. 112.

———, Dr. William, his case of phthisis pulmonalis cured by mercury, xix. 219.

HARRISON, Dr. Edward, on vaccine inoculation, v. 108; his remarks on the ineffective state of the practice of physic in Great Britain, xvi. 279; his circular letter on medical reform, xxii. 330; his memorial addressed to the Lords of the Treasury on medical education, xxiv. 303; his address to the Lincolnshire Benevolent Medical Society, on medical reform, 494; strictures on this address, xxv. 109; observations on his Bill, 216, 402, 492; on his persevering spirit in the pursuit of his object of medical reform, xxvi. 2; further observations on his Bill, 123; his remarks on an amended Bill for regulating mad-houses, xxxii. 1.

———, Dr. Charles, on endemic causes of disease, being an address delivered to the Medical Society of Horncastle, viii. 221.

HARRISON, Mr. his case of dolor faciei, x. 421; on vaccination, xiii. 331.

———, Dr. John, his case of hydrophobia, xviii. 236.

———, Dr. John Edward, his own case of rheumatic gout in the stomach and bowels, xix. 527; his successful treatment of syphilis without mercury, xxiv. 45; his cases of the first stage of cancer, and of fluor albus, 275.

HARRISON, Mr. Thomas, his examination of the merits of the Brunonian system, xvi. 182.

HARROLD, Mr. on fractures of the thigh, with a description of the instrument invented by him for, xv. 8, 339; on crusta lactea, 33; on the efficacy of oil of turpentine in burns, 147; on Mr. Hall's screw tourniquet, 149; on cold water in burns and scalds, 458; his reply to Dr. Kinglake on the latter subject, xvi. 115; his case of paralysis, xxviii. 453; remarks on, xxix. 462; his case of fractured vertebræ, xxv. 201; on the treatment of fractured thigh, xxvi. 120; his case of fractured spine, 371; of fractured os pubis, 375.

HARRUP, Mr. on the influenza of 1803, ix. 512; on the treatment of burns and scalds, xvii. 21; on dissolving the elastic gum, 454; on the improbability of the existence of two diseased actions in the same individual at the same time, xxi. 109; his case of colica pictonum, xxvi. 42; on tinea capitis, 203; on acute rheumatism, 449; on the construction of an electrical machine, xxviii. 265; his singular case of recovery in a patient, after the loss of a considerable portion of the brain, xxxii. 184.

HARTY, Dr. his observations on simple dysentery and its combinations, with an enquiry into the source of contagion in that and some diseases, xiv. 557.

HARVEY, Dr. Wm. his practice of midwifery, xxvii. 470; memoirs of, from notices contained in the Bodleian Library and Ashmolean Museum, xxxiii. 108.

HARVEYAN ORATION, for 1809, xxii. 438.

HASLAM, Dr. his observations on madness and melancholy, with practical remarks, xxi. 495.

HASTINGS, WARREN, esq. his testimonial of the very extraordinary method by which Colonel Martin cured himself of a stone in the bladder, i. 123.

———, Mr. his mode of applying blisters, xxvii. 452.

HATCHET, Mr. of N. A. his discovery of a new metal, or of an acidifiable oxyd, which he calls Columbium, vii. 477.

HATCHETT, Mr. his communication to the Royal Society on artificial tanning, xv. 295.

HAUPTMAN, Dr. Augustus, a celebrated chemist of the seventeenth century, was the first to ascribe the generation of formidable pestilential diseases to animalcula, xxiv. 23.

HAUY, M. his treatise of mineralogy, vii. 471; his ingenious mode of enabling blind men to write, viii. 572; his analysis of a new earth, xi. 285.

HAWES, Dr. the late, his zealous efforts to establish the Royal Humane Society, xxiii. 337.

HAWKER, Dr. on the ophthalmia of hot climates, followed by observations on a disease named by Mr. Chevalier catarrhous suffocations, or coryza trachealis, xii. 407; on epidemics, xiv. 337.

———, Mr. his discovery of the bones of a large fossil animal, xv. 391.

HAWKINS, A. esq. on the exotics which endure the open air in Devonshire, xxv. 248.

HAXBY, Dr. his medical cases, iii. 558; his singular case of aneurism of the aorta, xxxiii. 265; his letter in illustration of the above case, 449.

HAY, Dr. of Reading, Massachusetts, his account of a remarkable disposition to hemorrhage in many individuals of the same family, xxxiii. 9.

HAYGARTH, Dr. on fever arising from venereal poison, iii. 198; on the imagination, as a cause and a cure of the disorders of the body, exemplified by the employment of fictitious tractors, 277; on the prevention of infectious fevers, v. 582.

HEAD, its probable sensations after being severed from the body, i. 51; instead of beheading, another mode of execution recommended, by which the deadly blow, and the complete annihilation of the natural structure of the brain, may take place at the same instant, 52; injuries of the, cases of, described, iii. 30; of infants newly born, a peculiar sort of tumour observed on the, iv. 445; blisters applied to the, their great efficacy in cases of delirium, vi. 571; on ring-worm of the, xiii. 24; effusion into the, on the effects of antimonial powder in some diseases of, xvii. 557; extraordinary tumours on the, a case of, xxv. 165; the physical condition of the, its probable influence on that of the mind, xxix. 48.—*See* BRAIN *and* CRANIUM.

HEAD-ACH, the one usually termed sick head-ach, cured by the use of vegetable acids, i. 286; how to distinguish, when it arises from crudities in the stomach, v. 397; case of an inveterate, xii. 1; periodical, case of, removed by the solution of arsenic, xv. 506.

HEADLY, Mr. his two successful cases of premature delivery, where the pelvis was greatly deformed, v. 312; on angina maligna, and on the expediency of keeping up, in the treatment of this disease, an excitement on the tonsils, uvula, and fauces, 424.

———, Mr. Henry, on ophthalmia, xxiv. 130.

HEALTH, and Life, genius of, a popular work in the form of a pocket-book, for 1801, vi. 253; on, and on longevity, xiii. 527; circumstances which necessarily tend to promote, 530; rules for preserving, and for promoting longevity, 531; regulations for, in a community, ibid.; Board of, its establishment at Somerset Place, with a list of its members, xv. 191; manufactures prejudicial to, a report submitted to the French National Institute, xvi. 100; whether those which emit a disagreeable smell are prejudicial to, 176; Board of, Manchester, report of the, 338.

HEARING, difficulty of, proposed to be remedied by the application of galvanism, viii. 324; difficulty of, remedied in several instances by galvanism, ix. 142; theory of, by Alexander Walker, esq. in his rapid view of the original theory of phonics and musical sounds, xix. 402; observations on the restoration of, by the perforation of the tympanum, xxii. 191; organ of, its anatomy and physiology described, xxix. 81.

HEART, a particular affection of the, described, i. 298; extraordinary conformation of the, in a young girl, viii. 497; cursory remarks on various morbid affections of the, xi. 305; a singular case of malformation of the, xiii. 120; observations on the preceding case, and on the subject in general, 430; history of a peculiar morbid appearance in the, xv. 386; on a diminution of the area of the aperture of the, by which the left auricle communicated with the ventricle of the same side, xxii. 71; account of a very peculiar disease in the, 137; two cases of inflammation of, 252; observations on some of the most frequent and most important diseases of the, 505; case of a preternatural enlargement of the, with diseased pericardium, 513; observations on its functions, xxiii. 22; case of organic disease of the, 205; a collection of cases of organic diseases of the, with remarks, 273; case of inflammation of, xxiv. 415; the action of the, as it is influenced by the brain, physiological researches on, xxvi. 58; case of inflammation of, 396; ossification of, in a linnet, xxviii. 382; enlargement of the, remarks on, 157; observations on organic diseases of the, xxix. 52; diseases and organic lesion of the, xxx. 141; particular diseases of, xxxi. 217; the history of a foreign body discovered in, xxii. 37; case of rheumatism of, 220; of organic disease of, 305; of palpitations of, 307; of aneurism of, 389, 448; pathological researches on malformations of, 513; xxxiii. 49; congenital hernia of, 72; case of malformation of, xxxiv. 100.

HEAT, experiments made on, to prove that it is distinct from light, viii. 572; and light, on the comparative effects of, ix. 83; surprising qualities of a Spaniard in resisting a very high degree of, with details of the trials made before a Committee of the Institute and Medical School of Paris, x. 380; of climate, rarely produces disease without intemperance, xii. 78; disengaged by the compression of air, curious experiments on, repeated before the French National Institute, 575; used by several of the older surgeons in the treatment of burns or scalds, xvii. 131; observations on the effects of solar heat on the human body, xxiii. 193; high degrees of, their effects on the animal economy, xxiii. 307; on, as it is evolved during inflammation of the human body, xxx. 323; simple mode of obtaining a very intense degree of, 346; observations on radiant, 396.

———, *animal*, observations on, viii. 497; in typhus, &c. observations on, xii. 244; reflections on, xxi. 62; experiments and observations on the influence of the brain on the generation of, xxix. 141; on the production of, 441; experiments on the production of, xxxii. 132, 223, 233; xxxiii 113.

HEBB, Mr. his remarkable instance of natural small-pox in the case of a mother and her new-born infant, v. 536; his translation of Croissart's Treatise on the Diseases of the Heart, xxx. 141.

HEBERDEN, Dr. William, his Commentaries on the History and Cure of Diseases, viii. 179; observed apoplexy to occur frequently after a regular and severe fit of gout, xiii. 146; on the mortality of London, 150; delivers the Harveyan oration for 1809, xxii. 438; on a peculiar affection of the eyes, xxxi. 147.

HECKER, Dr. his theory of dentition in children, followed by that of Dr. Brandis, iv. 76; on a new disease called by him angina polyposa spasmodica, vi. 94.

———, Dr. J. Van, on the formation of bones, and on luxuriant callus, iv. 74.

HECTOR, Mr. on vaccination, xiii 52.

HEDERA, botanical description and medicinal qualities of, xii. 226.

HEDWIG, Professor, his ingenious mode of multiplying the *Fritillaria Regia* of Linnæus, i. 297; his new arrangement of ferns analytically described, v. 372.

HEILMAN, Dr. his new theory of galvanism founded on experiments, xviii. 245.

HEINEKE, Dr. on impeded deglutition, arising from organic derangement of the œsophagus, xxxi. 392.

HELLEBORUS, botanical description and medical qualities of, xvii. 375.

HELLWAG, Dr. his experiments on the application of galvanism to medicinal purposes, x. 333.

HELM, Mr. on the digestive power of the human stomach, xi. 44.

HELSHAM, Dr. his two remarkable cases of dysphagia, followed by two others of ischuria, iv. 477; his case of hydrocephalus internus, xii. 5.

HEMICRANIA, observations on, xxiv. 160.—*See* CRANIUM.

HEMINGWAY, Mr. on puerperal convulsions, xxx. 36.

HEMIPLEGIA, efficacy of galvanism in cases of, xiii. 373; successful treatment of, xxxii. 525.—*See* PARALYSIS.

HEMLOCK, extract of, its efficacy in the cure of tetterous eruptions, xi. 342; efficacy of, in the disease called tic douloureux, xxxi. 383.—*See* CICUTA.

HEMORRHAGE, Mr. Carlisle's mode of suppressing it from the lower extremities, i. 23; from the liver, fatal case of, iv. 65; petechiæ, cases of, vi. 233; xxvi. 153; several cases cured by phosphoric acid, ix. 388; on the origin of the use of the ligature for suppressing, xiii. 391; xiv. 40, 103, 111; from divided arteries, the process employed by nature in suppressing, xv. 463; remarks on the opinions of Petit, Morand, Sharp, Powteau, Gooch, Kirkland, White, and J. Bell, respecting the natural cessation of, 466; secondary, observations on, and on the ligature of the arteries after amputation, xvi. 79, 87; of the nose, a remedy for stopping, 191; uterine, practical observations on, with remarks on the management of the placenta, xviii. 281; uterine, and from the lungs, as well as chronic menorrhagia, effectually cured by the acetate of lead, xix. 8; nasal, an obstinate intermittent cured by, 53; efficacy of the acetate of lead in the treatment of, 69; hereditary, disposition to, existing in certain families, xx. 69; uterine, observations on, xxi. 30; from the stomach, case of, xxii. 180; xxiii. 290, 461, 493; from the urethra, case of a very copious, owing to the application of a bougie, armed with argentum nitratum, to a stricture, xxii. 341; Mr. Davies's paper on, examined, xxiii. 290; observations on the restraint of, xxxi. 453; efficacy of secale cornutum, or ergot, in the treatment of, xxxii. 97; use of opium in, 153; remarkable disposition to, existing in many individuals of the same family, xxxiii. 9; on the treatment of, 383, 462; xxxiv. 21.

HENDERSON, Stewart, Mr. his account of the means successfully employed by him to preserve the health of seamen, i. 91; his mention of the well-merited fame acquired by Captain Cook, as to the possibility of carrying a ship's crew through a variety of climates, for nearly four years, without losing one man by disease, ibid.; his narrative of the weather on the Jamaica station, during the years 1787, 88, 89, and a part of the year 90, i. 95; his practical remarks on the diseases of the West India station, 135, 236; his enquiry into the causes which produced diseases at the Cape of Good Hope, with the outline of a new plan of military hospitals, 453.

———————, Dr. John, on the noxious and fatal effects of septic gas on a family living near the place of its evolution, xvii. 225.

———————, Dr. A. his enquiry respecting Dr. Milward, xxvi. 456; his observations on the case of Ann Moore, xxix. 109; his memorial relative to that impostor, xxx. 187.

HENDY, Dr. his successful treatment of a case of hysteric phrenitis, arising from pulmonary inflammation, xiv. 391.

HENEY, Dr. his letter on the efficacy of the zanthoxylon, ii. 31.

HENNEN, Mr. his case of profuse epistaxis, xi. 549; on the use of sponge in ophthalmia, xii. 233.

HENNING, Dr. his remarkable case of elephantiasis in an aged female, ix. 289.

HENRY, Mr. his general view of the nature and objects of chemistry, and its application to arts and manufactures, iii. 189; on digitalis and opiate frictions, ii. 102; on the treatment of gonorrhœa, ix. 53; on the discovery of aromatic vinegar, 307.

HENSLER, Professor, on the leprosy of the West, in the middle ages, ix. 554.

HEPAR SULPHURIS, efficacy of, in tetterous eruptions, xxxii. 165; xxxiv. 257.

HEPATITIS, observations on, ii. 4; a case of, followed by bilious expectoration, xiv. 469; observations on the use of nitric acid in, xviii. 301; case of, and of gout, its effects in removing an apparent paralysis of the lower extremities, xxvi. 153; observations on chronic, xxviii. 95, 279, 300; case of, xxx. 190; of India, observations on the, and on the prevalent use of mercury in diseases of Great Britain, xxii. 415. —*See* LIVER.

HERACLEUM, botanical description and medical qualities of, xii. 365.

HERBAL, Dr. Thornton's New Family, xxiii. 424.

HERDMAN, Dr. on the management of infants, and on the treatment of their diseases, xi. 466; his plan for the improvement of dispensaries, and of the medical treatment of the poor, xxi. 36, 166.

HERHOLDT, M. his method of preventing asphyxia in new-born children, i 179; on respiration, with experiments on animals, vi. 467.

HERMAPHRODITE, dissection of an, in which the genital parts of both sexes were very perfect, ix. 70; observations on, relative to medical jurisprudence, xxxii. 332.—*See* MONSTROSITY.

———————— lamb, description of an, ii. 1.

HERMBSTAEDT, Professor, mode of purifying the atmosphere when loaded with acid gases, i. 46; on the practical study of botany, by means of chemical analysis, 72, 263, 377, 495; iii. 370; his observations on the very slightly marked line of demarcation between animal and vegetable life, i. 72; his very cheap and expeditious process for separating the mineral alkali, or soda, from common salt, iii. 376; on the separation of sebaceous matter from vegetables, v. 361; on the separation of the astringent principle from vegetables, 362; on that of the colouring principle from vegetables, 363; on that of the caustic principle from vegetables, 364; on that of tartarous acid from vegetables, 365; on that of the oxalic acid, 366; on that of the malic acid, ibid.; on that of the benzoic acid, ibid.; on that of the saccholactic acid, 367; on that of tartar and salt of sorrel, ibid.; on that of saltpetre, 368; on that of the sulphat and muriat of kali, or potass, 467; on the analysis of the vegetable fibre, ibid.; on the quantity of carbon contained in the coal of vegetables, 468; on the analysis of the remaining earth, ibid.; this subject concluded, 470; experiments made by, to obtain sugar from the indigenous plants of Germany, iv. 244, 347; his chemical experiments on the leaves and roots of the tulip-tree, (*Liriodendron tulipifera*), 376; notice of his new edition of experimental chemistry, v. 199; on the preparation of nourishing broths from fresh and dry bones, xii. 350; his cheap method of obtaining the sugar of the beet-roof, xvii. 197.

HERNIA, omental, singular case of, i. 156; strangulated, observations on, iii. 330; viii. 472; xiv. 206, 306; xvi. 45; xvii. 55; xxiv. 112, 193, 512; strictures on the usual practice in cases of strangulated, iv. 317; singular case of, in a cat, v. 105; a case of strangulated crural, in which the operation succeeded after the obstruction had continued six days, 113; scrotal strangulated, extraordinary case of, ix. 260; observations on, and on their radical cure, ix. 396; xxxiv. 107; congenital, case of, x. 33; case of, 248; crural, observations on, xi. 48; on the situation of, relatively to the crural bloodvessels, 54; on the situation of the stricture in incarcerated, 55; on the means proposed and employed for removing the stricture in incarcerated, 56; cases of, 120, 191; directions for performing the whole of the operation for incarcerated, under the various circumstances in which this may be necessary, 254; inguinal and congenital, anatomy and surgical treatment of, 279, 372; strangulated, case of, xii. 337; successful treatment of a case of hernia, xiv. 452; of another case, in which it was strangulated, 454; a case successfully treated, xv. 155; scrotal, remarkable case of, successfully terminated, 381; crural, a remarkable case of, xvi. 81; on the structure of the parts concerned in crural, 119, 172; xvii. 23; case of sphacelated, xvi. 178; case of hernia, xvii. 227; case of scrotal, 430; establishment of the London Rupture Society, xviii. 33; femoral incarcerated, case of, 45; Mr. Lawrence's prize essay on hernia for 1806, xix. 85; a case of crural, which terminated in gangrene, 501; strictures on a case of incarcerated femoral, 529; remarks on Mr. Lawrence's prize essay on, xx. 4; on the above case of crural, 63; on a case of umbilical, 312, 405; on a case of crural, 508; scrotal strangulated, two cases of, in which the reduction was greatly facilitated by the application of topical cold, xxii. 233; the slimy juice of snails proposed as a remedy for hernia, when the ruptured part can be returned and safely confined within the body, xxiii. 261; case of strangulated crural, operated on agreeably to the plan of Gimbernat, with observations, 379; new mode of reducing crural, 456; frequency of, with observations on the medical cure proposed by M. Tarenna, xxiv. 18; efficacy of ether in, xxv. 23; cases of, xxvii. 162; list of patients labouring under, relieved by the City of London Truss Society, xxviii. 39; of the brain, account of, 186; xxx. 74.

HERNIARIA, botanical description and medical qualities of, xii. 227.

HERPES, generic character of, xi. 230; vesicularis, description of, 234; phlyctænoides, described, 235; erysipelatosus, description of, 237; miliaris, described, 239; during vaccination, observations on, xvi. 242; exedens vermiculatus, case of, xxiv. 162; ulcerous, the danger of curing too precipitately, 219; eruptive, remedies useful in, xxv. 498; efficacy of arsenic in the treatment of, xxx. 22.

HESSE, Dr. his indefatigable zeal in following up the vaccine inoculation at Constantinople, viii. 95.

HEWITT, Mr. on calculous concretions of the tonsils, iii. 446.

HEWSON, Mr. the late celebrated anatomist, memoirs of, xxv. 59.

HEY, Mr. on the influenza of 1803, x. 194; his practical observations in surgery, 369, 466; on the disease named fungus hæmatodes, xi. 211.

HEYDECK, J. J. his trials of goat-pock inoculation, x. 339.

HEYWOOD, Dr. on diseased uterus, xxiii. 299.

HERZ, Dr. a most determined enemy of vaccine inoculation, his arguments against the introduction of a brutal virus into the human constitution, vi. 571.

HICKIN, Mr. his case of mania cured, xviii. 50.

HICKS, Mr. his strictures on Dr. Pearson's examination of the Report of the Committee of the House of Commons on the claims of remuneration for the vaccine-pock inoculation, ix. 481; his case of hydrophobia, xvii. 271; his case of trismus, 277; his reply to Mr. Ward's remarks on the case of hydrophobia, xviii. 258.

HIEGLITZ, Dr. Johann, his essays on scarlet fever, in which he asserts that it has of late years become more malignant and fatal than in former times, xxi. 252.

HIERARCHIUM, botanical description and medical qualities of, xix. 257.

HIGGS, Mr. on the management of draught horses, xxxiv. 420.

HIGHLANDERS, on the diseases of the, and on the simplicity of their mode of life, xiii. 28.

HIGHMORE, Mr. his case of a fœtus found in the abdomen of a young man, xxxiv 317.

HILDENBRANT, Professor, his chemical encyclopædia, iv. 74; his pocket-book for health, vi. 175; his edition of Boekmann's experiments on the action of phosphorus in different gases, vii. 374; his experiments on the preservation of the flsh of animals in the gases, xxxii. 435.

HILL, Mr. on angina pectoris, iv. 30; on cow pox, v. 152; on the diseases of the bones, vii. 415; on Mr. Ward's theory of the modus operandi of opium, ix 153; on the necessity of a nice discrimination between schirrous and cancerous swellings, and on the too frequent use of the knife in presumed cases of the latter, xii. 410.

——, Mr. G. N. on the inconvenience of healing wounds by the first intention, iii. 12; his further observations on opium, x. 533; on cancer, xii. 410; on contraction of the lower limbs, xv. 326; on medical reform, xvii. 23; on the efficacy of arsenic in a variety of chronic complaints, xxi. 149; his observations on the Liverpool controversy relative to a trial for murder, 399; on the effects of arsenic in the cure of diseases, xxii. 337; xxiii. 238; his case of deformity of the face and throat from burning, removed by chirurgical operations, xxvi. 158.

——, Mr. of Barnstaple, on the influenza of 1803, x. 195.

——, Mr. Samuel, on Mr. Goldson's pamphlet, and in reference to Dr. Macdonald, xii. 445; experiments made by, to prove that the cow-pox inoculation is a permanent security against small-pox, 473; his cases of variolous inoculation, and of vaccine inoculation, with a defence of the latter, xvi. 110.

——, Mr. of Portsea, on the effect of cutaneous eruptions, xiii. 537; on vaccination, xiv. 48.

——, Mr. R. his cases of the recurrence of natural small-pox in several individuals, xvii. 456.

HILL, Mr. his surgical remarks, xxv. 487; on ptyalism, xxvii. 275; on the beneficial effects of caustic issues in an affection of the spine, xxix. 182.

——, Rowland, Rev. in vindication of cow-pox inoculation, xvi. 82; on vaccination, xvii. 175, 329.

HIMLY, Professor, on the utility of the application of extract of hyosciamus, or toxicodendron, in paralysis of the pupil of the eye, vi. 275.

HIP, case of injury of the, with fracture of the neck of the thigh-bone, xxxiii. 469.—*See* JOINTS.

HIP-JOINT, a disease of the, efficacy of the Bath waters in the treatment of, xv. 565; an extensive caries of the, extraordinary symptoms attendant on, with exquisitely painful enlargement of the hip, xviii. 485.

HIPPOCRATES, his strong conviction of the beneficial effects of abstinence in obviating the approach of acute disease, i. 44; observations on his practice of medicine, iv. 151; eulogy on him, viii. 280; his Materia Medica, xii. 470; his remarks on erysipelas, 525; his opinion respecting the origin of epidemics, xiii. 209; his opinion respecting hereditary diseases, xxi. 330.

HIPPURITES, Sicilian, the shells of the, observations on, xxviii. 236.

HIRD, Dr. of Leeds, on the influenza of 1803, x. 518.

HOARSENESS, chronic, with deprivation of the voice, cured by the pellitory of Spain, v. 335; case of, cured by the application of galvanism, viii. 250.

HOBBES, Dr. of Swansea, on the epidemy of 1803, x. 289.

HODGE, Richard, his case of chorea sancti viti, presenting very singular phenomena, i. 21.

———, Mr. of Sidmouth, on the influenza of 1803, x. 197.

HODGSON, Mr. his case of epilepsy cured by electricity, v. 347; his case of fractured wrist, xxxii. 146; his case of popliteal aneurism, xxxiv. 116; his treatise on the diseases of arteries and veins, 129.

HODSON, Mr. his instrument for extracting a stone from the bladder, vii. 429.

HOFFMAN, M. his essay on fixed vegetable alkaline salts, with observations by Dr. Mitchil, xi. 404.

HOGBEN, Mr. on the early discharge of the liquor amnii, viii. 260; his series of anatomico-mechanical tables of the human gravid uterus, xxvii. 346.

HOLDER, Dr. his account of the extraordinary success of vaccine inoculation in the Island of Barbadoes, x. 424.

HOLLAND, Mr. his successful treatment of a case of violent nervous affection, xiv. 517.

HOLME, Rev. John, his analysis of the mineral called arragonite, xxix. 510.

HOLT, Rev. Mr. on the cow-pox, ii. 401.

HOLY OAK, (*Ilex Latifolium* of Linnæus,) the leaves of the, their tonic and resolvent effect, ii. 466.

HOLYOKE, Dr. his easy mode of preparing the sal aëratus, or carbonate of potass, i. 295.

HOME, Sir Everard, on the structure of the tongue, with an account of several cases of diseased tongue admitted into St. George's Hospital, xi. 269; his observations on cancer, connected with the histories of the disease, xiii. 361; receives from the Royal Society the Copleian medal, on which occasion the venerable President takes a brief but perspicuous retrospective view of Sir Everard's philosophical labours and physiological discoveries, xix. 191; his physiological researches on digestion, xx. 2; on the structure and uses of the spleen, 211; remarks on his mode of treating strictures of the urethra, xxi. 80; observations on the functions of the stomach and the spleen, xxii. 5, 17, 127; his anatomical discoveries relative to the squalus maximus of Linnæus, xxiv. 5; his fatal case of the bite of a rattlesnake, with the natural history of this particular snake, and the effects produced by its poison, 391; on the non-conducting powers of the thoracic duct and the spleen from the stomach to the bladder, xxv. 361; xxvii. 13; his discovery of a middle lobe of the prostate gland, xxviii. 6; his practical observations on the treatment of the diseases of the prostate gland, xxix. 226; on the narwall and its tusks, 427, 508; on the formation of fat in the intestines of living animals, xxxi. 43; oration instituted by him, and Dr. Baillie, in commemoration of the late John Hunter, 344; on the influence of the nerves on the pulsations of the arteries, xxxii. 248; on the functions of the brain, xxxiv. 43; strictures on this latter paper, 311; on the respiratory organs of some genera of vermes, and on the mode of generation of the lampreé and myxine, 165.

HONEY-STONE, analysis of the, vi. 189.

HOOPER, Dr. his Anatomist's Vade-Mecum, iii. 286; his compendious Medical Dictionary, v. 482; his anatomical plates of the bones and muscles, viii. 473; on the influenza as it prevailed in London in 1803, ix. 385; his work entitled the Physician's Vade-Mecum, remarks on, xxiii. 75.

———, Mr. his account of the diseases, and of dysentery more particularly, of the sick landed from Corunna, xxii. 428.

HOOPING-COUGH, remedy for, i. 84; observations on, and on the good effects of tincture of opium in, vii. 305; the efficacy of artificial musk in, viii. 41; a case of, superseded by small-pox, 426; efficacy of fixed alkali in, ix. 176; of a particular remedy in, xiv. 39; of nitrat of silver in, xvi. 16; domestic origin of, as it occurred in North America in 1805, xvii. 293; remarks on, xviii 566; its frequency and complication with other diseases in London, in the winter of 1807, xix. 91; efficacy of the enula campana in, 455; observations relative to the treatment of, xxiii. 71; prevalence of, in London, in 1809, 173; query relative to, 261; inquiry into the contagious property of, xxii. 177; on the efficacy of arsenical solution in, xxv. 115; the plant belladonna recommended in cases of, xxvi. 510; remedy for, xxvii. 102; remarks on the origin of, xxxiv. 127.

HOPE, Mr. on the effects of nitrous acid and opium in the cure of dysentery, iii. 413; his case of extra-uterine conception, vi. 360; recipe cited by, for the cure of the bite of a mad dog, xiii. 297.

HORNS, curious case in which two of these substances grew from a young female in good health, xxxi. 518.

HORSE, the common distemper in the glands of the head and throat of, either concomitant, or presaging epidemic or pestilential diseases, i. 79; experiments made at Copenhagen, by injecting remedies into the veins of, and by which diseases were cured which had been deemed incurable, particularly the staggers, v. 399; experiments on the urine of, viii. 476; catarrhal disease of, with the results of hot and cold regimen, xiii. 216; running, the process of training, and queries respecting, 536; bitten by a dog supposed to be mad, and which either died or was killed, interesting account of, xxiii. 1; the foot of the, structure and use of its different parts, with experiments exhibiting the changes produced in it by shoeing, and the causes of the apparent mystery of that art, 175, 338; not to be acted upon by emetics, xxiv. 271; on the treatment of pulmonary inflammation in, and on inoculating glanders and farcy, xxv. 281.

HORSE'S HEEL, the vaccine disease traced from the, by Drs. Jenner and Adams, with the result of their subsequent trials, xv. 582.

HORSFIELD, Mr. description of the rhus vernix, i. 108.

HORTUS KEWENSIS, or catalogue of the plants cultivated in the Royal Botanic Garden at Kew, xxiv. 428.

——— ELGINENSIS, near New York, account of the establishment and progress of the, xxvi. 162.

HOSACK, Dr. his case of tetanus cured by wine, iii. 569; his successful case of an operation for aneurism of the femoral artery, xxi. 424; on contagion, or an endeavour to draw a nice distinction between that and infection, xxii. 513; his Hortus Elginensis, or a catalogue of plants indigenous and exotic, cultivated in the Elgin Botanic Garden, near New York, xxvi. 162; his dissertation on croup, xxvii. 195; remarks on this dissertation, 353; his classification of diseases, xxix. 250; on the advantages of exposing wounds to the air after capital operations, and on schirrous tumours of the breast, xxxi. 540; on the peripneumonia typhodes prevalent in the United States, 471; on the surgery of the ancients, and on their claims to many of the reputed discoveries and improvements of modern times, xxxii. 111, 297.

HOSPICE DE L'HUMANITIE.—*See* HOTEL DIEU.

HOSPITAL, for the treatment of ulcerated legs, observations on the necessity of such an establishment, iii. 135.

———, Pennsylvanian, account of the, ix. 250.

———, Medico-chirurgical, establishment of a, and of a Lying-in Hospital for women, at Tubingen, x. 480.

———, named Addenbrooke's, at Cambridge, brief account of, xiv. 344.

———, Guy's, remarkable case of a sailor in, who had swallowed clasped knives, one of which, having taken a position directly across the stomach, produced death, xxii. 350.

HOSPITAL PUPIL, the, written with a view to facilitate the study of medicine and surgery, iv. 459.

HOSPITALS, Military, outline of a new plan for the construction of, i. 459; county, and other charitable institutions in Great Britain, a brief account of, viii. 121; instructions on the means of maintaining the salubrity and purifying the air of the wards of, applicable also to prisons, &c. xi. 98; medical, of Ireland, observations on, and on the means of supplying the poor more effectually with medical assistance in the remote parts of that kingdom, xv. 242; reply to these observations, 362; foreign, on the establishment of, xxv. 348; curious account of the establishment of Royal, extracted from Holinshed's Chronicles, xxvii. 306.

HOTEL DIEU, in Paris, since styled Hospice de l'Humanité, account of, i. 80.

HOTTENTOT, female, commonly called "the Hottentot Venus," observations on, xxvi. 15.

HOWARD, Mr. J. S. his two cases of the cure of dropsy by digitalis, vi. 418.

———, Mr. John, on a remarkable case of spina bifida, xiii. 496; his practical observations on cancer, xxvi. 501; xxvii. 67.

———, Mr. Luke, on the dangerous fraud committed by the substitution of glass of lead for glass of antimony, with his easy tests, xxiii. 439.

HOWE, Mr. on amputation, xxiii. 108, 479; his account of sea-scurvy, with the curative means employed on ship-board, 233.

HOWISON, Dr. gold medal awarded to, for his preparation of tan, made in the East Indies from the bark of the mangrove tree, xiii. 383.

HOWSHIP, Mr. his observations on lock-jaw with tetanus, with two cases, xxi. 180; his further observations, with a case of tetanus illustrative of the peculiar sympathies existing in the nervous system under the influence of disease, 407; his case of local inflammation and abscess connected with the pericardium, 265; his observations on the phenomena and treatment of tetanus, with a case showing the inefficacy of mercury in that complaint, 446; his case of tetanus, in which amputation failed in checking the fatal progress of the disease, xxii. 120; another fatal case, described by him, under circumstances essentially different, 122; his case of lock-jaw, in which the progress of the disease was arrested, and the complaint cured, by amputation, 123; his case of tetanus, arising from a wound, in which the exhibition of mercury was unsuccessful, 185; his case of tetanus, in which the immediate good effect of compression with the tourniquet was ascertained, but which terminated fatally, 324; his case of lock-jaw, from the irritation of an extensive wound, in which opium certainly checked the progress of the disease, 479; his observations on tetanus, xxiii. 15; his case of epilepsy, in which the recurrence of the paroxysm was prevented by the use of cold affusion, 20; his observations illustrative of the effects produced by the solar heat upon the human body, 193; his case of *coup de soleil*, 196; his second case of *coup de soleil*, 198; his third, and fatal case of *coup de soleil*, ibid.; his observations on convulsions, as a consequence of disease, 369; his case, in which convulsion, connected with great constitutional debility, was treated by the depleting system, 371; his case in which very slight derangement in the structure of the brain produced convulsions, 480; his observations on diseases of the brain, xxiv. 52, 89; his cases of metastasis, or translation of diseased action, 178.

HUBERT, M. his experiments on plants during the process of fecundation, xx. 499.

HUFELAND, Professor, his remarks on the influence of the Brunonian system of the practice of medicine, i. 41; the Brunonian system is, according to him, so replete with defects, that it does not merit the name of a system, and its theory essentially disagrees with matters of fact and experience, 42; on the efficacy of narcotic remedies in chronic inflammation of the eyes, 284; recommends the tincture prepared from the seeds of the thorn-apple as an excellent narcotic remedy, iii. 488; on two species of vomition of the milk observed in children, with the appropriate remedies, 585; his further remarks on the Brunonian practice, iv. 269; on the erysipelas of new-born children, v. 511; on the medical use of oil, vi. 69; his system of practical medicine for the use of lectures, &c. 170; recommends the oil of the walnut-kernel in herpetic and other cutaneous eruptions, ix. 94; also recommends the chenopodium ambrosioides in nervous diseases, spasms, paralyses, and asthmatic complaints, ibid.; on the efficacy of the *flores zinci* externally applied in excoriations and cutaneous ulcerations, xi. 93; his new tincture of digitalis, ibid.; recommends the tincture of cantharides in hooping-cough, 191; his case of incarcerated hernia, in which he employed the digitalis with great success, 191; on the efficacy of savine in chronic gout, xxv. 544; on that of the plant belladonna in hooping-cough, xxvi. 510.

HUGGAN, Dr. A. on the effects of venæsection and opium, ii. 233; on the croup, iii. 56; on vaccine inoculation, 241; his opinions relative to venæsection and opium examined, 326; further remarks on vaccine inoculation, 344; on venæsection, &c. 538; on a case of small-pox which followed vaccine inoculation, iv. 343; on the exhibition of mercury in cases of hydrophobia, where the excision of the bitten part cannot be safely attempted, xiv. 538; on the efficacy of cold water in convulsions, xv. 52; on tapping at the navel, xvii. 209; his observations on hydrophobia, xviii. 396.

HUGHES, Dr. on the influenza of 1803, x. 107.

———, Mr. on cow-pox, i. 318.

HUGO, Mr. on the influenza of 1803, x. 311; on vaccination, xiii. 49; xvii. 365; his case of ruptured uterus, xix. 209; on small-pox after vaccination, xxxii. 478.

HULL, Dr. his British Flora, ii. 489; his defence of the Cæsarian operation, his description of the female pelvis, and Dr. Osborn's opinions examined by him relative to embryulcia, and his account of delivery by embryotomy, iv. 168; his essay on phlegmasia dolens examined, v. 4; on phlegmasia dolens, followed by an account of the symptoms, causes, and cure, of peritonitis puerperalis et conjunctiva, &c. 92; in justification of his essay on phlegmasia dolens, 165; his observations on phlegmasia dolens, vi. 51, 135, 220; his case of intus susceptio (the Ileus volvulus of Sauvages), vii. 33; his observations on crural hernia, xi. 48; on its situation relatively to the crural blood-vessels, 54; on the situation of the stricture in incarcerated crural hernia, 55; on the means proposed and employed for removing the stricture in incarcerated crural hernia, 56; a case of, 120; his directions for performing the whole of the operation for incarcerated hernia, under the various circumstances in which this may be necessary, 254.

———, Mr. Thomas, his account of a singular case of hæmoptoe, iii. 424.

HUMANE SOCIETY, Royal.—*See* Societies.

HUMBEY, Mr. his description of a case of lusus naturæ, iii. 137; on vaccine inoculation, vi. 45.

HUMBOLDT, M. A. Von, his experiments on the stimulated muscular and nervous fibre, with conjectures on the chemical process of life in the animal and vegetable kingdoms, i. 53; is considered in Germany as the founder of a rational system of animal chemistry, ibid.; demonstrates that the irritability of matter is dependent, not on the gases alone, but on the common operation and antagonism of a plurality of substances, 54; explains the functions of vegetables and animals by an ingenious mode of combining the new facts of galvanism, 145; his experiments to determine the quantity of carbonic acid contained in atmospherical air, iv. 83; on the illumination of rotten wood, ibid.; his anthracometer, an instrument for measuring the quantity of carbonic acid contained in atmospherical air, 179; his experiments on the irritated muscular and nervous fibre, with conjectures on the chemical process of life in the animal and vegetable kingdoms, x. 55; details relative to his interesting travels in the interior of America, xiv. 187; his curious experiments on the torpedo, xv. 295; his additional inquiries into the respiration of fishes, xxiii. 451; he is named foreign associate of the French National Institute, xxiv. 434; his observations on the physical causes which arrest the progress of Mexican population, xxv. 230; xxvi. 10.

HUME, Mr. Joseph, his observations on the pharmacopœia, xi. 298; xii. 55; xiii. 217; on the application of the cupping-glass in cases of hydrophobia, xii. 344, 527; on the employment of sulphur as a vermifuge, xix. 382; his new mode of detecting arsenic, xxii. 87; his test for arsenic, xxiii. 448; his second test, xxiv. 145; on arsenical preparations, 403; on the Eau Medicinale, xxvi. 185; on his arsenical test, xxviii. 109, 284; xxix. 46; remarks on his paper on barytes, xxxiii. 35; his reply, 218; his improved method of preparing tartarized antimony, 484.

HUMERUS, observations on luxations of the, xiii. 520.

HUMPAGE, Mr. his extraordinary case of the tumefaction of one of the labia, v. 53.

HUMPHRIES, Dr. his history of a case of paralysis cured by lightning, with minutes of a case of yaws cured by the negro slaves on a plantation, xix. 119.

HUMULUS LUPULUS, botanical description and medical qualities of, xii. 228; efficacy of, in various complaints, xiii. 432; xviii. 403; its efficacy in gout and rheumatic affections, xxv. 168; xxix. 294.

HUNGARY, description of the plants of, iv. 368.

HUNGER, Professor Dumas on the cause of, x. 20.

HUNNEHMAN, Mr. on the progress of vaccine inoculation in Hanover, iii. 471.

HUNOLDT, Dr. his analysis of, and his experiments on, cow-pock matter, ix. 67; on the efficacy of charcoal in cases of herpetic exanthemata, xii. 283; his observations on the restoration of hearing by the perforation of the tympanum, xxii. 191.

HUNT, Mr. his salutary cautions respecting the gout, with strictures on the doctrines maintained by Dr. Kinglake, xiv. 260; observations on his anatomical reflections, xviii. 7.

HUNTER, Mr. Alex. his history of a successful case of the extirpation of an inverted uterus, iii. 554; on acetate of lead, ix. 173; on the influenza of 1803, x. 233.

———, A. M. Mr. his essay on the diseases incident to Indian seamen on long voyages, xv. 289.

HUNTER, John, Mr. defence of his doctrines and language, viii. 412; xxxiv. 29; his opinions on the origin of new morbid poisons, xiii. 79; his opinions respecting yaws, 80; his remark on surgical operations, xv. 355; his opinions on the utility of cinchona in gangrene, 493; his opinions on the influence of local disease on the general system, xvi. 272; remarks on his opinions respecting the means of restoring suspended animation, 532; remarks on his opinions on the influence of cold on the action of mercury, xvii. 378; remarks on his experiments to shew the identity of the cause of syphilis and gonorrhœa, 573; remarks on his distinction of strictures of the urethra, xviii. 381; the disposal of his museum, 386; his observations on the digestion of the stomach after death, xix. 170, 173; xxi. 348, 426; xxiii. 399; xxxi. 2, 6; remarks on the character of his talents, xix. 467; various remarks on his doctrines, 563; remarks on his doctrine of the imcompatibility of the synchronous existence of two diseased actions, xx. 181; his opinions on the different character of disease according to the different texture of the affected part, 184; remarks on his opinions on some points of the history and nature of syphilis, 560; xxxiv. 227; remarks on his mode of treating stricture of the urethra, xxi. 79, 161, 310, 372; xxii. 517; his opinion respecting the cause of the motion of the heart, xxii. 255, 336; his doctrine of sympathy, xxiii. 244; xxxiv. 66; his opinion on the act of death, 404; his doctrine of hectic fever, xxv. 78; xxxiv. 29; remarks on his theory of life, xxxii. 421; remarks on his theory of the vitality of the blood, xxxiii. 276; xxxiv. 29; observations on the act of deglutition and vomiting, xxxiii. 401; his opinions respecting mortification, xxxiv. 138.

HUNTER, William, Dr. his observations on the rare occurrence of hydrophobia after the bite of a supposed rabid animal, xii. 389; his theory of the nature of hydatids, 553; taught in his lectures that ulcers in the urethra were never the cause of gonorrhœa, xiii. 321.

HUNTERIAN ORATION, instituted by Dr. Baillie and Sir Everard Home, xxxi. 344.

HUTCHINS, Mr. on galvanism, ix. 445.

HUTCHINSON, Mr. John, on cow-pox, v. 359.

———, Mr. Benjamin, on a case of lacerated femur, vi. 127; his case of steatomatous tumour, ix. 17.

———, Dr. on popliteal aneurism, xxvi. 241; xxvii. 177; his case of diseased brain, produced by external violence, xxxi. 506.

HYACINTH, analysis of the bulbs of the, viii. 475; botanical description and uses of, xiv. 67.

HYDATIDS, uterine, an extraordinary case of, ii. 447; observations on cancerous, vi. 193; Dr. Adams on, xii. 552; Dr. J. Hunter's theory of, 553; of the breast, Sir E. Home's opinion respecting the origin of, xiii. 365; contained in a herniary sac, case of xxi. 278; contained in the bladder, case of, 226; case of the glottis being completely closed up by, xxii. 140; cancerous, in the breast, case of, 355; discharged by stool and vomiting, xxvi. 506; natural history of, xxvii. 510; of the uterus, case of, 333; observations on, 340.—*See* Tumors.

HYDRARGIUM PHOSPHORATUM, a new and powerful remedy in the secondary stages of syphilis, i. 280.

HYDRARGIRIA, the mercurial disease, description of the, xxvi. 28.

HYDRARGYRUS ACETATUS, utility of, in chronic exanthemata, iii. 585; sublimatus corrosivus, its efficacy in amaurosis, iv. 184; muriatus mitis, (calomel,) experiments made on, xii. 574.—*See* MERCURY.

HYDRARTHRUS, or dropsy of the joints, chiefly confined to the knee, its cause pointed out, xix. 84.

HYDROCELE, cured by electricity, ii. 12; cured by injections with a simple apparatus, viii. 175; on the treatment of, xiii. 389; a case of, which terminated fatally, remarks on, xviii. 531; of the tunica vaginalis testis, particular treatment of three cases of, xxii. 205; chronic, practical observations on, xxv. 175; acute, observations on, 331; on spurious, 353; on chronic of the tunica vaginalis testis, 355; on aqueous injection in, xxvi. 110; curious case of the radical use of, xxviii. 424; case of absorption of the water in, from mental agitation, xxxiv. 515.

HYDROCEPHALUS, internus, a remarkable case of, ii. 131; two cases of, 143; an ambiguous case of, 247; observations on the treatment of, 254, 258, 327; a successful case of, iii. 61; internus, observations on, 113; case of, 325; several cases of, 517; case of, v. 341; and other diseases of the lymphatic system, counteracted by the use of mercury, 450; a remarkable case of, vi. 6; two remarkable cases of internus, viii. 98; treatment of two cases of, in the Finsbury Dispensary, x. 74; case of internus, 150; cases of, xi. 401; xii. 5; the remote cause of the disease called chorea sancti viti, or St. Vitus's dance, xiii. 407; a remarkable case of, xiv. 42; case of, cured by digitalis, 237; Dr. Witte's cases of, xv. 28, 380; observations on, 188; remarks on Dr. Quin's essay on, xvii. 67; observations on, xviii. 93; two cases of, 212; case of, 481; fatal case of, xix. 22; fatal case of internus, 91; case of, cured by copious depletion, 217; observations on, especially as connected with teething, xxi. 83; its great prevalence at Edinburgh in 1809, 259; original remarks on the pathological theory, and practical treatment of, internus, xxii. 378; on certain theories relative to, xxiii. 25; remarks on internus, 171; Dr. Robertson on, xxiv. 210; case of, xxxi. 69; observations on, with a case, xxxiv. 193; case of chronic, in which the mental faculties remained unimpaired until a short time before death, 516.

HYDROGEN, sulphurated, gas, its prevalence in the bowels of drunkards, xii. 87; efficacy of, in stomachic complaints, xv. 274; its union with metals, curious experiments on, xix. 574.

HYDROPHOBIA, its nature and cure considered, ii. 173; proposal to establish in Paris an hospital for the cure of, iii. 489; fatal termination of a case of, iv. 55; mode of preventing and curing, adopted by the inhabitants of the eastern parts of Gallicia, 87; a case successfully treated by copious bleeding and mercury, 122; observations on, v. 291; opiate frictions recommended in, vi. 479; inflammatory nature of, and on the employment of stramonium and cantharides in, viii. 88; an attempt to demonstrate the non-existence of, ix. 390; symptoms of, removed by the application of galvanism, xi. 191; submersion recommended in cases of, 459; efficacy of galvanism in, xii. 259, 512; the application of cupping-glasses to the wound, proposed as a preventive by Mr. Hume, xii. 344; remarks on, 385; on its production by the influence of the imagination, 388; the rarity of its occurrence after the bite of a supposed rabid animal, 389; observations on Mr. Hume's hypothesis relative to, 436; on the utility of the cupping-glass in cases of, 527; xiii. 242; a severe case of, xiii. 155; remedy to prevent it, 297; on the danger of administering arsenic in cases of, and on the fallacy of some other remedies, xiv. 30; case successfully treated by copious bleeding and mercury, 122; cases of, in which the exhibition of mercury promised success, 338; observations on, and on the deficiency of the prophylactic and curative means by which that malady may be encountered, xv. 409; xvi. 437; xvii. 183; cases of, xvii. 218, 271; cases and cures of, selected from the Gentleman's Magazine, 286; use of volatile alkali as a counter-poison in, 293; observations on, and on the practice of the late Dr. Percival, 328; case of, 344; observations on, with the indications of cure, 353, 432, 457; plan of an institution for investigating the cause, symptoms, and cure of, 479; case of, xviii. 236; observations on, 238, 396; history of a case of, 475; case of, with its fatal termination, 551; observations on, xix. 117; Dr. Moseley's case of, examined, 241; treatment of a case of, which terminated fatally, 491; one of the most severe and fatal of the morbid derangements in the class of neuroses, xx 8; case of, which terminated fatally, 195; fatal case of, 198; fatal case of, from the bite of a cat, attended by strongly marked symptoms, 201; observations on the case of, related by Dr. Bardsley, 209; the lichen caninus, or ash-colour ground liverwort, recommended as a remedy against, 271; two cases of, by Mr. Armstrong, who recommends the application of active caustic where excision cannot be readily had recourse to, 323; case of, in which coma supervened, with very decided symptoms of the genuine disease, from which the patient recovered, 326; on preventing death in cases of, according to the proposal of Dr. Physick, by making an artificial opening into the wind-pipe, 358; fatal case of, at Kensington, 396; strictures on Mr. Armstrong's cases of, 452; on Mr. Loudhouter's case of, 454; observations on, 468; fatal case of, in London, 481; uncertain case of, in an infant aged three months, 531; observations on, 543; xxi. 23; particulars of a fatal case of, 53; general observations on the nature and cure of, 134; Mr. Taunton's communication relative to, drawn from MSS. in his possession, 219; fatal case of, 268; observations on, 271, 334, 519; a small gold medal offered by Dr. Roberton for the best essay on, 521; remarks on, xxii. 53; distinction between, and the disease called by nosologists hydrophobia spontanea, an interesting case of which is cited, 113; Dr. Marcet's case of, with the appearances after death, 157; case of, with particulars of the appearances on dissection, 251; on the impossibility of its being caused by the bite of any rabid animal, a treatise, 343; remarkable case of, 344; details of presumed cases of, in horses bitten by a dog supposed to be mad, and which either died or were killed, xxiii. 1; essay on, under the title of Tetanus Rabiensis, with the curative means pointed out, 41; Mr. Ward's theory of, with the mode of treatment it indicates, 72; the latter claims the priority over Dr. Girard, the doctrines and opinions entertained by whom he was the first to adopt, 149; case of supposed, 224; observations on, 459; very prevalent in France since the commencement of 1810, 525; observations on the case of, reported by Dr. Marcet, xxiv. 29; case bearing a resemblance to, from the bite of a cat, xxv. 61; dissertations on, to which the Jacksonian prize was adjudged, 543; observations on, xxvi. 206; xxviii. 249, [illegible]; cases of, 350, 449; xxix. 23; observations on, 42; on blood-letting in the treatment of, 105; observations on, 272; case of, 285; analogy between, and gastritis, 454; cases of, 485; observations on, xxxi. 1; fatal cases of, 166, 122, 514; cases of, xxxiii. 12; xxxiv. 426; pathology of, xxxiii. 139; on the advantages of bleeding in, 178; suggestions relative to the treatment of, 293; cases of, both real and imaginary, 317; case of, xxxiv. 177.

HYDROPS MEDULLÆ SPINALIS, commonly called spina bifida, case of, xxi. 66.—*See* Spina Bifida.

HYDROPS VAGUS, or vagrant dropsy, on the, ii. 182.

HYDROPTHALMIA, case of, xxiii. 125; observations on, xxv. 163.

HYDROSULPHURETS, practical observations on, xxx. 256.

HYDROTHORAX, observations on, iv. 384; case of, ix. 417; cases of, in which the efficacy of digitalis in this disease is demonstrated, ix. 98; xvi. 229, 353; case of, its history and fatal termination, xxi. 443; observations on, xxiii. 330; inquiry into the nature, causes, and cure of, illustrated by cases, 376; cases of, successfully treated, xxix. 369; xxxii. 101; fatal case of, 134; case of, xxxiv. 103.

HYOSCYAMUS, oil of, useful in hæmoptysis, iii. 576; extract of, its utility in cases of the paralysis of the pupil of the eye, vi. 275; botanical description and medical qualities of, xii. 132.

HYPERIAN, botanical description and medical qualities of, xix. 164.

HYPOCHŒRUS, botanical description and medical qualities of, xix. 257.

HYPOCHONDRIASIS, considered as a gouty affection of the stomach, i. 147; singular instance of, xiv. 277; more prevalent in Great Britain than in former times, xx. 7; the more general employment of assafœtida recommended in cases of, xxi. 477; the cause of, traced to hepatic diseases, xxvi. 212; common in Iceland, xxvii. 123.

HYPOCHONDRIUM, calculus found in a tumour of the, xxxiv. 437.

HYPOPION, observations on, xvii. 81.

HYSLOP, Mr. the Jacksonian prize awarded to, by the Royal College of Surgeons, for his dissertation on the diseases of the eye, and its appendages, xix. 575.

HYSTERALGIA, history of a fatal case of, with the appearances on dissection, xix. 550.

HYSTERIA, considered as connected with gout, i. 151; a case of, promptly cured by the exhibition of musk, v. 235; case of, accompanied by an extraordinary optical illusion, xiv. 117; proceeding from menstrual obstructions, tansy *(tanacetum)* recommended in cases of, xix. 354; more prevalent in Great Britain than in former times, xx. 7; efficacy of assafœtida in cases of, xxi. 477; two remarkable cases of, as they occurred in Edinburgh, xxii. 171, 172; case of, successfully treated, xxxii. 104.

HYSTEROTOMY, remarks on two unsuccessful cases of, v. 353.—*See* CÆSAREAN OPERATION.

I.

IACCA, its efficacy in the treatment of venereal ulcers, xiv. 431.

IBBETSON, Mrs. her observations and experiments on the physiology of vegetables, xxvi. 170.

ICE, experiments on the temperature of, i. 169; its efficacy in various disorders, vi. 106.

ICE VINEGAR, a new mode of preparing, v. 395.

ICELAND, mineralogy of, as described by Sir George Mackensie, xxv. 362; description of the diseases most prevalent in, xxvii. 118.

ICONOCLASTIS, his Pethox Parvus, a small pamphlet in ridicule of those who are still attached to small-pox inoculation, xviii. 384.

ICTERITIA, an uncommon disease of infants, described, xix. 189.

IDIOSYNCRASY, two remarkable cases of, iii. 586; singular insance of, xvii. 295; extraordinary case of, xxi. 486; remarkable instances of, with respect to the influence of different species of aliment, xxii. 403.

IDIOTISM, remarks on, as connected with medical jurisprudence, xxxii. 315.

IENITE, a new mineral, discovered in the island of Elba, xx. 97.

IETROS, on quacks and empiricism, xi. 554; xii. 137, 212, 423; reply to, in defence of Dr. Brodum, xiii. 258.

ILEUM, singular case of thickening and stricture of the, xxxi. 71.

IMAGINATION, its influence on the physical functions of the body, xvii. 477.

IMHOFF, Professor, his instructions for the proper application of electricity in diseases, i. 54.

IMPEY, Mr. Elijah, on the efficacy of vitriolated zinc, combined with opium, in cases of dysentery, ix. 55.

IMPOTENCE, remarks on, by Dr. Mahon, in his work on medical jurisprudence, ix. 70.

IMPREGNATION, remarkable case of distinct, i, 399; remarks on animal, ii. 152; observations on a remarkable case of, xiv. 416.—*See* GENERATION.

INCURABLES, on the establishment of an hospital for, xvii. 361.

INDIA-CORN, experiments made on the stalks of, iv. 247.

INDIANS, characteristics of the Mexican, xxvi. 13; complexion of the, how influenced by climate, 14; their surprising vigour and muscular strength, xxv. 246; North American, curious mode of curing diseases practised by a tribe of, xxxi. 209.

INDIES, East and West, observations on diseases of, iv. 354; xv. 83; xviii. 124, 499; xix. 114, 115, 199; xxi. 75, 76; on the hepatitis of, xxii. 415.—*See* CLIMATES.

INDIGESTION.—*See* DIGESTION, DYSPEPSIA, *and* STOMACH.

INFANTICIDE, reflections on, with a case, showing the danger of forming too hasty a decision in supposed cases, viii. 447; observations on, ix. 448; W. R. on, 449.

INFECTION, arising from the disturbance and too superficial interment of bodies in church-yards, x. 174; venereal, researches on, xx. 560; morbid, observations on, xxxiv. 97.—*See* CONTAGION *and* CONTAGIOUS DISEASES.

INFLAMMATION, some general observations on, xviii. 80; as it is connected with fever, observations on, xix. 168; xx. 174, 183; treatise on, more particularly as connected with diseases of the eye, xxii. 167; various observations on, xxiv. 230.—*See the distinct Organs and Appellations of* DISEASES.

INFLUENZA, as it prevailed in Paris in 1803, account of the, ix. 287; observations on the, 366; as it prevailed in London and its environs, as well as in various parts of Great Britain and Ireland at the same time, with historical abstracts concerning the catarrhal fevers of 1762, 1775, and 1782, 377, 385; on the report made in Paris, where it was named the *gripe*, 419; reports on the, collected from various parts of Great Britain and Ireland, 460, 505, 509, 512, 515, 517, 522; x. 46, 80, 97, 98, 99, 101, 102, 103, 105, 107, 108, 109, 110, 112, 113, 114, 115, 116, 117, 118, 119, 122, 125, 126, 127, 129, 193, 194, 195, 197, 198, 199, 200, 201, 202, 203, 204, 206, 208, 209, 210, 211, 212, 213, 214, 215, 217, 219, 220, 221, 222, 223, 224, 225, 226, 228, 229, 230, 232, 233, 289, 291, 293, 294, 295, 296, 301, 303, 304, 305, 306, 308, 311, 312, 355, 385, 386, 387, 389, 390, 392, 393, 395, 396, 397, 398, 399, 405, 408, 409, 517, 518, 519, 520, 521, 522, 523, 525, 527, 528, 529; xi. 81, 488; xiii. 209; xiv. 226.

INJECTIONS, new liquid for anatomical preparations, xiv. 52; remarks on, in the treatment of hydrocele, xxvi. 110; remarks on the use of, in gonorrhœa, xvii. 547.

INKS, writing, report made on it to the French National Institute, with trials on an indelible ink, xxvii. 224.

INOCULATION, experiments with the virus of goat-pox, x. 339; with measles, experiments on, xiv. 363; xix. 153; various observations on, xiv. 506; of a cow, with small-pox virus, xv. 48, 58; xix. 153; observations on Mr. Bryce's test-puncture, xv. 171; various remarks on, 255, 516; xix. 377; xxii. 173; with the matter of gonorrhœa, 312; with variola after cow-pox, xx. 258; mode of effecting it for cow-pox, xxiv. 482.

INSANITY, review of Dr. Arnold's Treatise on, xvii. 84; general observations on, xviii. 182; its origin in disorder of the stomach and bowels, xix. 94; on the metaphysical causes of, xxi. 496; compared with dreaming, 502; pathology of, 501.—*See* Mental Alienation.

INSECT, venomous, two cases of fever induced by the bite of a, xix. 220.

INSECTS, iconographic description of, as they are preserved in the museums of Natural History, in Paris, iv. 450; very acute sense of smell possessed by, vi. 340; on a new genus of, named by naturalists atracto ceros, viii. 282; *larvæ* of those of the *tenebrio molitor* and *musca domestica minor*, expressed from the bowels, xxv. 447; anatomy of, important observations on the, in a paper read before the French National Institute, xxxi. 515; memoir on the compound and smooth or simple eyes of, and on the manner in which these two species of eyes concur in vision, xxxii. 314, 400, 500.

INSTITUTE, French National, its particular attention to the learned foreigners sent to Paris by the allies of France, to assist in obtaining a general uniformity of weights and measures, i. 195 —*See* Societies.

INSTITUTION, clinical, at Wurtzburg, annals of the, ii. 484; medico-pneumatic, observations on the, 488; Royal, lectures on galvanic electricity, given at the, iv. 113; vaccine-pock, London, report of the committee of the, on the practice during the years 1800, 1801, and 1802, with engravings, x. 187; rules of the extended medical, for the benefit of the sick and drooping poor, 571; plan of the Royal Sussex Jennerian, xii. 477; for the cure and prevention of contagious fever in the metropolis, report made by the, in May 1805, xiv. 340; Royal Sussex Jennerian, its annual report for 1805, 346; cow-pox, at Nottingham, proceedings of the, xv. 251.

INSTRUMENTS, new, for the extraction of teeth, i. 13; surgical, invented by Dr. Clark, his account of, xii. 543; for the extraction of the fœtus, xxii. 103; for the operation for popliteal aneurism, xxvi. 246.

INTEGUMENTS of the human body, observations on the, xxxi. 458.

INTERMITTENTS.—*See* Ague *and* Fever.

INTESTINES, cases of introsusception of, vii. 33; xi. 145; xv. 470; xviii. 175, 350; xxii. 158; case of sphacelus of, vii. 430; account of an extraordinary cure of a wound of the, xviii. 501; organic injury of the, terminating in phthisis pulmonalis, xxii. 150; case of stricture of the, 392; observations on inflammation of the, with a case, xxiii. 54; on the process of nature in repairing injuries of the, xxviii. 8; case of ulceration of, 247; the lower, on the formation of fat in, xxix. 507; extraordinary case of schirrus in the, arising from hairs remaining in the canal, xxx. 515.

INVALID, the, with the obvious means of enjoying health and long life, xiii. 81.

IODE, or violaceous gas, discovery of the, xxxi. 255.

IODINE, experiments and observations on, xxxiii 37.

IPECACUANHA, efficacy of, in indigestions and dyspeptic complaints, xvi. 569; the effluvium of, its pernicious effect, xxi. 486; xxiii. 199, 318, 447; xxiv. 60.

IPSWICH, the station of the troops on their return from Holland, statistical report of the Walcheren fever, as it prevailed there, xxv. 1.

IRELAND, Dr. Joshua, on fungus hæmatodes, and on the practicability of the success of its curative treatment, xxv. 303.

IRIS, on the division of the, xv. 4. xvi. 1; on the inflammation of the, and on the influence of the extract of the belladonna, to prevent the consequent obliteration of the pupil, xvi. 55; procedentia of, xvii. 81; two memoirs on the organization of the, and on artificial pupils, 403; on the vibration of the, xxii. 468; inflammation of the, or ophthalmia iridis, described, xxvii. 232. —*See* Eye.

——, (the plant) *fulva*, a new species, account of the, xxix. 260; *prismatica*, another new species, (both from North America,) 261.

IRON, on the medicinal properties of, i. 389; citrat of, its medicinal qualities, 401; ammoniated, or martial flowers, easy mode of preparing, vi. 468; oxydation or rusting of, a new mode of preventing, xi. 286; carbonate of, its efficacy in the cure of cancer, xvii. 201; filings of, their efficacy in a case of inflexibility of the articulations, xix. 6; its conversion into steel, observations on, xx. 347; carbonate of, its efficacy as a tonic, 447; a particular preparation of it, efficacious in obstinate coughs, xxii. 70; pyrolignite of, the acid so called, its utility in the arts, 439; carbonate of, recommended in cancer, xxiv. 138; xxix. 13; in ulcerated uterus, 332; moulds, method of discharging from cotton, xxx. 84.

IRRITABILITY, its causes considered, x. 25, 252.

IRWINE, the late Dr. William, his observations on diseases, chiefly as they occur in Sicily, xxvii. 65.

ISATIS, botanical description and medical qualities of, xviii. 173.

ISCHIA, Island of, efficacy of its warm baths in venereal exostoses, xxiii. 498.

ISCHIAS, commonly called a hip case, efficacy of the Bath waters in the treatment of, xv. 565.

ISCHURIA, observations on, iii. 331; cases of, 479; case of, xiv. 252; severe cases of, xviii. 570; observations on, and on the introduction of the catheter in cases of, xiv. 448; Dr. Cuming's case of, strictures on, xv. 36; severe case of, xix. 227; remarkable case of, xx. 115; case of, from a contusion, xxiv. 381; observations on, xxix. 353; xxx. 44, 498.

ISENFLAMM, M. on the constituent parts of the marrow of bones, vi. 470.

ISLAND, at the western extremity of Table-Bay, sudden disappearance of an, after an earthquake, xxiii. 263, 526; xxiv. 87; new, afterwards named Sabrina Island, discovered among the group of the Azores, xxvii. 80.

ISSUES, the inflammation and stimulus produced by, considered as contributing greatly to the cure of cases of paralysis of the lower extremities, xxi. 109; caustic, observations on the application of, in an affection of the spine, xxix. 182.

ITALY, brief account of the state of medical and physical literature, ix. 434.

ITARD, Dr. his account of the savage of Aveyron, viii. 376.

ITCH, the repulsion of, followed by a singular case of dropsy, which was cured by sulphur, i. 191; effectually cured by the *enula helenium*, 298; a new preparation, named sulphurated soap, recommended for the cure of, xxxii. 79; whether caused by insects? 496; new formula pointed out for the cure of, xxxiii. 246; another new mode of treating, 515; observations on, xxiv. 126.

ITCHINGS, from particular causes, danger arising from their sudden subsidence through the effect of external applications, xxiv. 125, 214.

### J.

JACCA, its use in the cure of venereal ulcers, xiv. 431.

JACKSON, Dr. on the causes, symptoms, and cure of fever, i. 467; his case of sphacelus of the intestine, &c. vii. 430; on the constitution of the medical department of the British army, with a detail of hospital management, and an appendix on the causes of fevers, xiii. 275, 472, 564; xiv. 85, 179; his system for the medical department of the army, xiv. 547; on cold affusion in fever, xx. 81, 164; on the virtue of the spider's-web in suspending the course of intermittents, xxi. 353; xxii. 369; on the efficacy of the field plant eyebright, in dimness of sight, xxiii. 104.

JACKSON, Dr. S. H. remarks on his work on the Gibraltar fever, xviii. 8.

———, J. G. Esq. his account of the plague of West Barbary, xxii. 193.

———, Mr. of Stonesby, Leicestershire, his case of the cure of cataract, xix. 102.

———, Dr. James, on the morbid effects of dentition, xxix. 51; his discourse on the tic douloureux, xxxi. 179, 383; his cases of a particular disease which prevailed in certain districts of the United States, xxxiii. 27; on inflammation in the mucous membrane of the alimentary canal, xxxiv. 18, 100.

JACOBI, Dr. on tussis convulsia, and its treatment, xv. 222.

JACOBUS, on the metallic basis of alkalies, xxi. 27; strictures on, in vindication of the discoveries of Sir Humphry Davy, 129.

JALAP, memoir on, with an appendix of observations, xxi. 392.

JAMES, Dr. his powder examined, with a method of preparing, in the humid way, a similar substance, vi. 518.

———, Mr. of Wrington, on the influenza of 1803, x. 105.

———, Mr. of Hereford, on the importance of the nervous system in the animal economy, xxxiii. 198.

JAMESON, Professor, treatise on Cheltenham waters, xxiii. 508; his papers on subjects of mineralogy, xxix. 430.

———, Mr. discovery of a new acid, vii. 381.

JAMES'S POWDER, analysis of, by a Neapolitan chemist, xxi. 438; combined with opium, efficacy of, in cases of tetanus, xxxi. 73.

JAPANESE, account of their mode of treating tympanitis, vii. 236.

JAQUES, *frere*, account of, xi. 9.

JARDINE, Mr. new invented surgical instruments described, viii. 482; his letter to Mr. Savigny, 487; on the extraction of teeth, x. 240; on surgical instruments, xii. 198; his improved trephine, 201; his improved raspatory, 202; his improved lenticular, 203; his successful treatment of a case of collapsed brain, xiv. 289.

JARROLD, Dr. philosophical, physiological, and political dissertations on man, xviii. 178; observations on his work entitled Anthropologia, xxii. 8.

JAUNDICE, observations on the cure of, iii 94; arising from an hitherto undescribed cause, cases of, vi. 29; cases of, arising from a schirrus of the pancreas, 218, 312; puerperal, two cases of, viii. 358; in infants, remarks on, xi. 184; efficacy of the nitric acid in the treatment of, xvii. 17; case of, successfully treated, xxxii. 106.

JAW, dislocation of the, iv. 503; case of luxated, xxxi. 187.

JAYMES, M. case of the cure of vomica pulmonum, xxxii. 120.

JEFFRIES, Dr. case of purpura hæmorrhagica, xxix. 419.

JELLY, efficacy of animal, in the treatment of intermittent fevers, xii. 431.

JENKINSON, Mr. on the efficacy of the opiate friction, vi. 432; on the use of myrrh, steel, &c. with his ingenious mode of coating pills, viii. 48; on the cure of rheumatism, xi. 492; on the efficacy of a solution of arsenic in chronic rheumatism, xiii. 523.

———, Mr. of Sutton Ashford, his cases of vaccine inoculation, viii. 197.

———, of Oxford, his facts determining the efficacy of Fowler's mineral solution in chronic rheumatism, xviii. 250; on the efficacy of the solution of arsenic in rheumatism, xix. 476.

———, Mr. on abstinence from bodily exercise, in the treatment of phthisis pulmonalis, xxi 227; on repose in cases of phthisis pulmonalis, xxii. 89; on the effects of digitalis in the treatment of that disease, 93; on the particular diseases in which arsenic ought to be administered, 337.

JENNER, Dr. on cow-pox, i. 313; on the eruptions said to attend the vaccine disease, iii. 101; his letter to Mr. Shorter on vaccine inoculation, 348; his continuation of facts and observations relative to the variolæ vaccinæ, or cow-pox, 378; his address to the public on the advantages of vaccine inoculation, with the objections to it refuted, iv. 69; presented with a medal by the Naval Medical Officers, v. 432; on the origin of vaccine inoculation, 505; report of the Committee of the House of Commons on his discovery of vaccine inoculation, vii. 485; appendix, 489; viii. 17, 141; valuable diamond ring presented to him by the Empress Dowager of Russia, viii. 480; letter signed by him, and by Mr. Ring, in answer to the letter of Lord Hardwicke, on the subject of establishing a Jennerian Society in Dublin, ix. 541; on the modifications of the vaccine vesicle, xii. 97; diploma conferred on him by the Imperial University of Wilna, xiii. 427; on herpetic eruptions, 541; letter from Dr. Spens to, electing him an Honorary Fellow of the Royal College of Physicians of Edinburgh, xvii. 139; letter to, from Dr. Stuart, a Fellow of the College, 140; remuneration proposed to, by Dr. Valentine, of Marseilles, xviii. 341; letter to, on the abuse of variolous inoculation, and on the falsehoods disseminated relative to vaccination, xix. 356; his observations on a canine epizootic, xxii. 240; his two cases of small-pox infection communicated to the fœtus in utero, 242.

JENNER, Mr. the Rev. on vaccine inoculation, v. 402.

———, Mr. on the spurious vaccine pustule, vii. 203; description of his footstool for injuries of the lower extremity, xxx. 174.

JENNINGS, Rev. Dr. on the effects of laborious exercise in the cure of pulmonary consumption, xviii. 448.

JEVANS, Mr. his singular case of spontaneous recovery from a crippled state bordering on paralysis, xxii. 237.

JIGGERS, account of that insect, viii. 455.

JOCKIES, observations on the training of, xiii. 533; queries concerning, 536.

JOHN, Professor, analysis of certain animal and vegetable substances, xxxii. 434; on chronic acid, and on its unknown combinations with certain bases, xxxiii. 33.

JOHNSON, Dr. Samuel, contempt of the opinion generally received of the influence of the air on the human frame, xix. 475; anecdote of, to prove that he was "just, not mad," xxi. 501.

———, Dr. James, essay on the influence of tropical climates, xxx. 227; his case illustrative of the good effects of cold applications to ulcers, 511.

JOHNSON, Mr. C. T. practical essay on cancer, xxiv. 72.

JOHNSTON, Dr. narrative of a man who subsisted on large quantities of raw flesh, iii. 209.

JOHNSTONE, Dr. on madness, iv. 258.

———, Dr. John, account of the discovery of the power of mineral-acid vapours, to destroy contagion, x. 78; his reply to Dr. Carmichael Smyth, with a further account of the discovery of the power of mineral acids, in a state of gas, to destroy contagion, xv. 373.

JOHNSTONE, Mr. on the efficacy of the steam of vinegar, and on that of the muriatic fumigation in cases of malignant epidemics, vii. 424.

JOINTS, observations on their diseases, ix. 374, 568; on the caries of the, and on the utility of amputating the extremities of the bones in cases of caries, x. 557; wounds of, observations on, xi. 395; ankle, case of a compound fracture of the, xviii. 162.—*See the different* JOINTS.

JONES, Dr. on the process employed by nature in suppressing the hemorrhage from divided arteries, and on the use of the ligature, xv. 463; xvi 87; his case of aphonia successfully treated, xxii. 252; his observations on ligatures, and on the natural means by which hemorrhages are suppressed, xxiii. 253.

———, Mr. of Montgomery, on the influenza of 1803, x. 107.

———, Mr. of Pembroke, on the influenza of 1803, x. 113.

———, Mr. Edward, on the efficacy of nitrat of silver in hooping-cough, xvi. 16.

———, Mr. of Howden, on the effects of arsenic, and on the difficulty of detecting it in the stomach after death, xxiii. 384; xxiv. 59, 274.

———, Mr. account of a child born in Wales without eye-balls, xxv. 277.

———, Mr. John, of Guisbro', singular case of twins, xxv. 311.

———, Mr. William, case of puerperal convulsions, xxx. 455; his singular case of abdominal inflammation, xxxi. 371.

———, Mr. R. case of tetanus, xxiv. 40; his cases in which a large quantity of laudanum was swallowed, xxxiii. 350.

JORDENS, Dr. remedy in cases of chronic ophthalmy, iv. 88.

JOSEPHI, Professor, remarkable account of a fifteen years' pregnancy, during which the child was contained in the urinary bladder, xiv. 519.

JOSEPHUS, account of a tree of the Balm of Gilead having been brought to Jerusalem by the Queen of Saba, and presented to King Solomon, i. 33.

JOUVILLE, M. his notices relative to the mineralogy of the island of Ceylon, xviii. 483.

JUCH, M. on the acid of sugar, ii. 298; on the muriate of barytes, iii. 581.

JUDEA, Balsam of, or Balm of Gilead, once grew in many parts of Palestine, but has since been totally lost there, i. 35.

JUKES, Mr. observations on the removal of cartilage, with the description of an instrument contrived by him for that purpose, xxvii. 372; on certain varieties in the arterial system, xxviii. 440.

JULLION, Mr. case of hydrocephalus internus, x. 150.

JUNCKER, Professor, on vaccine inoculation, iv. 141; on tympanitis, vii. 231.

JUNIPERUS, botanical description and medical qualities of, xviii. 370.

JURISPRUDENCE, medical treatise on, ix. 67; observations on, xxi. 100; xxxi. 230.

JUSTICE, courts of, on the opinions delivered in, vii. 283.

JUVENIS, remarks on reading, as a means to acquire medical knowledge, xii. 506; on a case of ulceration following venæsection, xv. 348.

K.

KAEUFER, M. cases of the cure of mania by belladonna, ii 72.

KALI, explanation of its nature when imperfectly saturated with carbonic acid, iv. 375; purum, successful application of, as a caustic, in strictures of the urethra, xi. 477; preparatum, its diuretic qualities and virtues in hydrothorax, xxiii. 334.—*See* Potash.

KAMSKATKA, a fermented liquor procured by the inhabitants of, from the infusion of the epilobium, xiv. 171.

KANT, Professor, notice of his treatise on the faculty of the mind to conquer the sensations produced by diseases, by means of the simple act of the will, i. 192; concise analysis of the above work, 259; his system of regimen, with a view to prolong life, 259, 493; his opinion of the organic powers of bodies, vii. 461.

KARSTEN, M. on the peculiar acid contained in cork, vi. 276.

KASTELYN, M. on the crystallization of potash, &c. ii. 81; on sublimed ammonia, 82; on sulphate of potash, iii. 169; on magnesia, 170.

KEATE, Mr. his evidence before the Committee of the House of Commons in favour of Dr. Jenner's discovery, viii. 149; his testimony relative to particular documents on vaccine inoculation, 150.

KEIL, Dr. his observations and experiments on torpid animals, notice of, xxiv. 9.

KEITH, Rev. Patrick, notice of his paper on the cotyledons of grasses, xxix. 510.

KELCH, M. his galvanic experiments on the body of a criminal, xi. 94.

KELLIE, Dr. George, his historical and critical analysis of the functions of the skin, xiv. 84; his case of suspended animation successfully treated, 267; his case and treatment of chronic rheumatism reviewed, xix. 556.

KELLY, Mr. George, his case of sphacelated hernia reviewed, xvi. 178.

KELLSON, Mr. of Seven Oaks, on vaccine inoculation, iv. 21; his remarkable case of extra-uterine pregnancy, xi. 293; notice of his pamphlet on cutaneous complaints, xxxiv. 78.

KENNON, Mrs. midwife to the Princess Dowager of Wales, anecdote of, xxvii. 330.

KENT, establishment of a general hospital in, viii. 123.

KENTISH, Dr. Edward, his successful mode of treating burns and scalds, iii. 206, 296; his notice to medical practitioners on this subject, 262; his observations on burns, in refutation of the opinions entertained by Sir William Earle and Sir Walter Farquhar, iv. 452; his cases of cancer, with observations on the carbonate of lime in the treatment of that disease, ix. 380; efficacy of his method of treating burns, xiii. 14; his views relative to gout and its treatment, 350; on the efficacy of oil of turpentine in burns, xiv. 396; observations on his mode of treating burns and scalds, xv. 342; on the treatment of burns and scalds, xvi. 8; his treatment of burns and scalds contrasted with that of Dr. Kinglake, xvii. 3, 21, 321; critically examined, 440; his opinion relative to the scars formed in burns and scalds, xviii. 412; his reply to Dr. Wood's charge of literary piracy, xix. 417; Dr. Wood in reply to, 519; observations on the controversy between, and Dr. Wood, xx. 53; his reply to Dr. Wood, 56; on dispensaries of heat, xxiv. 358; on the vapour bath, with the particulars of a case in which it was successfully employed, xxv. 317; his account of baths, and of a Madeira-house at Bristol, xxxii. 61.

KENWORTHY, Mr. his case of obstruction of the urethra, xxi. 225; history of a case of vicarious menstruation, xxv. 25.

KERMES, mineral, new process for preparing, xiv. 112; process for obtaining, prize offered by the Pharmaceutical Society of Paris for a, xviii. 35.

KERRISON, Mr. particulars of his fatal case of hydrophobia, xxxii. 431; his case of tic douloureux, 433.

KEUTSCH, Dr. his details relative to oil frictions in yellow-fever, xi. 480; xii. 382.

KEW, Royal Botanic Garden at, review of the catalogue of the plants in the, xxiv. 428.

KEY, Mr. his remarks on some cases of supposed cow-pock failure, xvi. 460; Mr. Ring, in reply to, 459; his cases of cow-pock failure, xx. 61; his remarks on Mr. Bellingham's case of hydrophobia, with arguments in favour of bleeding in that disease, xxxiii. 178.

KEYT, Captain, his statement of the introduction of the vaccine virus into the Island of Ceylon, ix. 391.

KIDD, Dr. his analysis of a new mineral found in Cornwall, xvi. 95; his outlines of mineralogy announced, xxi. 86.

———, Mr. W. L. his observations on hydrophobia, xiii. 459.

KIDDERMINSTER, the malignant fever which prevailed in, effectually corrected by the vapour of the muriatic acid, viii. 186.

KIDNEYS, particular diseases of the, and of the bladder, examined and explained by dissections, v. 284; remarkable degeneration of the, in a case of diabetes, 3[illegible]3; a case of diseased, ix. 438; diseases of the, how distinguished from those of the surrounding parts, xv. 573; case of diseased, xvii. 181.

KIDNEY VETCH, botanical description of, xix. 76.

KIEL, the University of, the first to abrogate the custom of writing the inaugural dissertations in Latin, i. 88; the medical faculty of, their report in favour of Dr. Jenner's discovery, xvii. 190.

KILIAN, Dr. C. J. notice of his genius of Life and Health, for the year 1801, vi. 253.

KILLER, Mr. controverts the efficacy of digitalis, vi. 133, 322.

KING, Dr. W. his remarks on inoculation, for scarlatina anginosa, xiii. 167.

———, Dr. David, notice of his cases showing the deleterious effects of the irrespirable gases, xxvi. 162.

———, Mr. J. his account of the diseases in the Pneumatic Institution, Bristol, vii. 301.

———, Mr. of Bath, his case of gun-shot wound, ix. 165; of inflammation in the maxillary sinus, 423; of spina bifida, x. 237.

———, Mr. South Carolina, his case of elephantiasis, xvi. 362.

———, Mr. R. H. his case of small-pox after vaccination, xxx. 33.

KINGLAKE, Dr. Robert, on the nature and properties of vital power, ii. 127; on a particular case of muscular inflammation, 285; on the effects of digitalis, iii. 120; on digitalis, and on its insufficiency in the last stage of pulmonary consumption, iv. 61; on the delivery of the placenta, 156; on the medicinal efficacy of sulphuric acid in chronic eruptions, 482; his reply to Mr. S. Thomas's criticism on his paper relative to parturition, 487; on epilepsy, and on the inefficacy of argentum nitratum in its cure, v. 435; on the cure of arthritic affections, vi. 454; his observations on dyspepsia, vii. 425; on sedative efficiency, 522; on the treatment of gout by cold applications, ix. 116; his vindication of this treatment, 442; his observations on the influenza of 1803, 517; x. 306; his observations on reduced temperature, x. 357; his correction of a mis-statement made by a correspondent, 530; his remarks on digitalis, xi. 59; a reply to, 150; his answer, 241; his dissertation on gout reviewed, xii. 561; on a particular case of gout, in which the cool treatment was followed, xiii. 231; on another case, in which very irregular symptoms were manifested, 357; strictures on his reply to the history of two cases, said to have proved fatal in consequence of the external use of ice and cold water, 460; his reply to Dr. Scott, of Newbury, in vindication of the cool treatment in gout, 512; his explanation relative to a fatal case of gout in which the hot treatment was followed up, xiv. 72; he announces an extensive variety of additional cases in proof of the salutary effects of the refrigerant treatment, 192; his reply to Mr. Mantal's observations on the nature and cure of gout, 215; Mr. Mantal, in answer to, 455; 497; on the radical identity of the variolous and vaccine forms of disease, xv. 48; strictures on his theory of the gout, 181; on his paper relative to the variolous and vaccine forms of disease, 219; his observations on the effects of the heating treatment of burns and scalds, 342; his remarks on the doubtful origin of cow-pock matter, 539; on burns and scalds, in reply to Mr. Harrold, xvi. 17; on the cooling treatment of burns and scalds, 460, 526; an attempt to reconcile his treatment of burns and scalds with that of Dr. Kentish, xvii. 3; his strictures on the nature and cure of gout, announced, 200; his remarks on the Angustura bark, 428; his treatment of gout examined, xviii. 52; a case of burn treated by his method, 152; on gout, in reply to Mr. Pope, 240; his treatment of burns and scalds examined, xix. 17; further observations on that subject, and on the treatment recommended by Dr. Kentish, 54; his observations on the effects of cinchona bark in acute gout and rheumatism, 427; a few words addressed to, 535; his reply on the treatment of gout, xx. 65; his observations on the use and abuse of blood letting, 391; his remarks on admissible criticism, with a tribute to the memory of the late Dr. Beddoes, xxi. 89; on the infectious nature of the last stage of phthisis pulmonalis, 193; on the salutary effects of topical cold in two cases of strangulated hernia, xxii. 253; on the influence of cold, applied to the sternum, in restraining bleeding from the nose, 327; his case of an enormous tumour in the cavity of the abdomen, xxiv. 105; his two cases illustrative of the destructive effects of a foreign body admitted into the trachea, 269; on the seeming analogy between hydrophobia and gastritis, xxviii. 273; on morbid sensation, 441; his further observations on the nature of hydrophobia, xxix. 42; his ideas on its analogy with gastritis anticipated by Mr. Barrett, 454; on the efficacy of arsenic in herpetic affections, xxx. 23; his reply to Mr. Aber, on Mr. Barrett's paper, 96; on the altered specific powers of vaccine and variolous matter, xxxii. 180; on the terebinthinate remedy proposed by Dr. Brenan in puerperal fever, xxxiii. 179; his observations on vaccine and variolous inoculation, 257; on general and local disease, 459; on the cooling treatment of gout, xxxiv. 6; on morbid infection, 97; on the efficacy of spirit of turpentine in puerperal fever, 183; on obstetric practice, 291; his reply to N. W. on topical cold in gouty inflammation, 361.

KINGTON, Mr. his case of luxated jaw, xxxi. 187.

KINNING, Mr. on the epidemic disease which prevailed in the garrison at Gibraltar, with particulars of the treatment, xiii. 193.

KINO, experiments on, xi. 276; its recent introduction into medicine, xxii. 361.

KIRBY, Rev. W. notice of the preparation of his Introduction to Entomology, xxiii. 262.

KIRCHER, among the first to maintain the doctrine of animalcular action on human bodies, in plague and febrile diseases, xxiv. 23.

KIRKLAND, Dr. Thomas, his opinion relative to the cause of true apoplexy examined, vii. 90; notice of his appendix to an inquiry into the present state of medical surgery, xxix. 174.

KIRWAN, Mr. his essay on mineral waters, iii. 562.

KITAIBEL, Dr. his description of the plants of Hungary, iv. 368.

KITE, Mr. remarks on his case of cataract cured by electricity, xvi. 1.

KITSON, Mr. his case of tic douloureux reviewed, xvi. 178; his case of scirrhous ulceration of the pharynx and œsophagus described, xviii. 475.

KLAPROTH, Professor, his discovery of a new acid, iii. 583; his contributions to the chemical knowledge of mineral bodies, ix. 472; his chemical analysis of diabetic urine, xi. 174; his discovery of a new vegetable salt, 288; his observations on meteoric, stony, and metalline substances, 351; his analysis of a Swedish fossil, in which a new earth was discovered, xii. 69; his discovery of a new vegetable acid, 92; his new mode of analyzing tin ores, xv. 295; his analysis of native cinnabar, xvi. 575; analysis of his paper on the solubility of white oxyde of arsenic in water, xxxii 253.

KLEIN, Dr. his case of the Cæsarean operation, with the remark that, of one hundred and sixteen cases ninety had been successful, v. 584.

KLOPSTOCK, M. on the treatment of the Cossacks in a peculiar disease of horses, xxxiv. 437.

KNACKSTAEDT, Professor, describes the *enula helenium* as an effectual remedy for tinea, itch, and other cutaneous disorders, i. 298.

KNAWELL, hoary, its medicinal properties, xv. 263.

KNEBEL, Dr. notice of his work on the history of medical science, ii. 397; his method of curing intermittents, iv. 81.

KNEE-JOINT, treatise on the morbid affections of the, viii. 470; two cases of diseased, relieved by electricity, ix. 568; cases of enlargement of the, critically examined, xxii. 515.

KNIGHT, T. A. Esq. his proposal to confirm the merits of Dr. Jenner's discovery, vii. 378; the Copleyan medal conferred on, for his numerous discoveries in vegetable physiology, xvii. 196; his improved method of cultivating the alpine strawberry, xxiv. 87; his details respecting a new and expeditious mode of budding, xxv. 253; notice of his experiments on the manner in which plants shoot forth their radicles, 452; remarks on the obvious analogy between some of the organs of animals and those of plants, xxvi. 7; his indefatigable researches into the physiology of vegetables, 170; connected view of his theory of vegetation, 171.

KNIPE, Mr. on a case of amputation above the wrist, by double incision, iv. 415; on epilepsy, v. 503.

KNIVES, swallowed by a sailor at Guy's Hospital, remarkable case of, xxii. 350.

KNOT-GRASS, its botanical description and medicinal qualities, xv. 67, 262.

KNOWLES, Dr. J. S. his observations on vaccination, xvi. 332; his appointment of physician to the Royal Jennerian Society, 478; correspondence between, and Dr. Clarke, on the subject of vaccine inoculation, xvii. 368.

————, Mr. his successful mode of treating leeches, xxiii. 321; his remarkable case of plica polonica, 388; his case of ascites, xxiv. 140; his case of diarrhœa, xxvi. 183; remarks on the above case, and on some others from the same author, 387; his reply to the above, xxvii. 42; strictures on his cases, 188; his reply to the latter, 370.

KNOX, Dr. Robert, notice of his paper on the relation between the time of the day and various functions of the human body, xxxiv. 63.

KOCH, Dr. his testimony of the surprising effects of the avens root in the cure of intermittents, i. 188.

KOENIG, Charles, Esq. his communication relative to the practice of midwifery among the modern Greeks, vi. 151; notice of his paper relative to the human skeleton from Guadaloupe, xxxi. 338.

KOPP, Dr. his account of an obstinate case of dolor faciei, xv. 230.

KOTZEBUE, forgery committed on, in the details given of his illness and recovery, vii. 93.

KOUMISS, how prepared by the Tartars, xxv. 250.

KRAMERIA TRIANDRIA, its medicinal qualities described, xiv. 127.

KRASKOVITZ, M. his address to the Royal Jennerian Society, xiii. 479.

KRETSCHMAR, Frederic, his essay on the effects of medicines, vii. 80.

KRUGER, M. his new method of preparing concentrated vinegar, vi. 474; his nervous tincture of Bestucheff, ibid.; his new processes for obtaining concentrated vinegar, and for the preparation of the milk of sulphur, xii. 111.

KUHN, Professor, his observations on hydrocephalus internus reviewed, xvii. 188.

KURSWIG, Dr. his successful case of the Cæsarean operation, v. 584.

KYNION, Mr. on swelled legs, and on the utility of bandages, &c. v. 242.

### L.

LABATTS, Dr. Samuel, his compendium of vaccination reviewed, xiv. 84.

LABIA, pudendi, ecchymosis of the, a singular case described, xi. 42; case of inoculated cow-pock succeeded by a confluent eruption of genuine vaccine vesicles on the, 385; extraordinary tumefaction of, v. 53.

LABORIOUS EXERCISES, salutary effects of, in pulmonary consumption, xviii. 448.

LABOUR.—*See* Delivery.

LABOUR-PAINS, extraordinary effects of the secale cornutum, or ergot, in exciting, xxxii. 90.

LABRADOR, the coast of, notice relative to its geology, xxviii 73.

LAC SULPHURIS, remarks on the preparation of, xi. 437; new process for preparing the, xii. 111.

LACEPEDE, M. his natural history of fishes, i. 203; his memoir on two species of non-descript oviparous quadrupeds, viii. 283.

LACERTA AGILIS, curious particulars relative to the natural history of the, xxvi. 27; medicinally employed, 250.

LA CHARITE, *hospice de,* general account of, i. 81.

LACONIA, rapid progress of vaccination in, xii. 449.

LACRETELLE, his natural history of salamanders, v. 177.

LACROIX, M. his account of an extra-uterine conception, ii. 480; analysis of snow-water, iii. 90.

LACTUCA VIROSA, the extract of, recommended as a remedy for catarrhal complaints, xxxiii. 161.

LAFFECTEUR, M. his essay on the moral and physical maladies of women, i. 310.

LAFOURCE, Dr. A. Leveque, his observations on an amaurosis and cophosis, attended by very peculiar symptoms, xxv. 510.

LAFUENTE, Dr. his new mode of curing yellow-fever analysed, xiv. 531.

LAGRANGE, M. on the nature and properties of styrax, i. 163; his analysis of the leaves of senna, iii. 85; his analysis of ambergris, xi. 167; also of the substance called tannin, xvii. 392; notice of his pharmaceutical report on the acetate of potash, &c. xxiv. 224; his analytical essay on the scammonies of Aleppo and Smyrna, xxv. 156.

LA GRAVE, M. his experiments on the galvanic pile, x. 63.

LAIR, Pierre-Aimée, his essay on human combustion from the abuse of spirituous liquors, iv. 44.

LAMARK, notice of his work on physics and chemical philosophy, i. 109; on the essential particles of bodies, iii. 89.

LAMB, Mr. successful trial of his apparatus for distilling fresh-water from sea-water, xx. 193.

———, hermaphrodite, description of an, with an engraving, ii. 1.

LAMBE, Dr. William, his researches into the properties of spring-water, xi. 462; his reply to the critique of the Editors of the Journal on the subject of these researches, 570; his case of inveterate head-ach, successfully treated, xii. 1; strictures on his medical and experimental enquiry into the origin, symptoms, and cure of constitutional diseases, xv. 74; analysis of his reports on the effects of a peculiar regimen on scirrhous tumours and cancerous ulcers, xxi. 508; xxii. 79; his defence of the peculiar plan of regimen suggested by him for the treatment of cancerous affections, xxiv. 306; Mr. Royston in reply to, 363; his paper on arsenic, read before the Royal Society, xxvii. 513.

LAMBERT, Mr. Daniel, of Leicester, his extraordinary weight and bulk, xv. 582.

LAMERT, Dr. particulars relative to, xii. 425.

LAMP, notice of a, for preventing explosions in coal-mines by the combustion of carburetted hydrogen gas, xxx. 82.

LAMPADIUS, M. his discovery of the substance called by him alcohol of sulphur, xxix. 509, 518.

LAMPSADIUS, Professor, his experiments on the sugar of the beet-root, with a curious analysis, iv. 366.

LANCASHIRE, particulars of a medical spring in, near the village of Flookborough, xxv. 84.

LANCET, description of a new, for dividing the inflamed vessels in cases of ophthalmia, xxii. 42.

LANCISI, his account of a malignant epidemy, which, in 1695, ravaged a particular district of the city of Rome, xvi. 123.

LANE, Mr. John, his narrative of a singular disorder which affected the crew of one of the ships under the command of Sir Thomas Cavendish, in his second voyage to Magellanica, in 1591, xxxi. 306.

LANG, Dr. on the management and preservation of the eyes, xiv. 11.

LANGFORD, Mr. of West Shefford, particulars of his second and fatal attack of natural small-pox, after having had that disease to a violent degree in his infancy, xv. 439.

LANGSLOW, Dr. his explanatory observations on apoplexy, vii. 77; his replies, on that subject, to Dr. Girdlestone and Mr. Crowfoot, 274; to Mr. Wilkinson, 275; to Mr. Atkinson, 277; his further replies to these gentlemen, 455; Mr. Crowfoot, in reply to, viii. 115; his remarks on the remuneration proposed to be made to Dr. Jenner, 164; his historical sketch of the controversy on apoplexy, &c. 379.

————, Mr. on the case of apoplexy published by Mr. Crowfoot, vi. 552.

LANGSTAFF, Mr. his history of a case of introsusception analysed, xviii. 175; notice of his account of two cases of gonorrhœa, attended with unusually severe symptoms, xxv. 445.

LANGUAGE, cursory remarks on that of modern medical writers, xxvii. 496, 513.

LANOIX, M on the dangerous effect of cutting the hair during the convalescence from acute diseases, iii. 367.

LANSDOWN, Mr. his method of preparing canvas for flexible tubes for conveying fresh air into coal-mines, &c. vii. 198.

LAPLANDERS, useful application made by the, of the leaves of the *rumex*, or common sorrel, xiv. 71; details relative to the diseases of the, xxxi. 51.

LARDNER, Mr. W. notice of his case of obliteration of the internal jugular vein, xxvii. 163.

LARKSPUR, wild, botanical description of the, xvii. 71.

LARREY, Baron, observations on the use of muriate of barytes, i. 469; his details relative to a peculiar kind of leeches, swallowed and stopped in different parts of the throat, ix. 262; his observations on the guinea-worm, xii. 434; on the atrophy of the testicles, as it was observed in Egypt, 518; on the inoculation of blennorrhœa, in cases of sudden suppression of that discharge, 519; strictures on his observations, xiii. 150; his observations on the sarcocele of Egypt, with cases, xxv. 289.

LARYNGITIS, particulars of a case of, at the Hotel Dieu, Paris, xxxii. 166; xxxiv. 255.—*See* CYNANCHE TRACHEALIS.

LARYNX, case of divided, v. 346; case of, successfully treated, xi. 63; on the formation of the, in eunuchs, xii. 575; case of ulceration of the, xxxii. 25.

LASCARS, or Indian seamen, essay on the diseases incidental to, strictures on the, xv. 289.

LASKEY, Mr. his remarks on the London Museum, Bridges-street, Covent-garden, xviii. 434.

LASSUS, M. his remarkable case of the sudden and deleterious effects of opium, v. 491; on umbilical hernia, vi. 278; memoir by, on the morbific elongation of the tongue out of the mouth, 353, 435, 522; his case of a woman poisoned with opium, xvii. 422.

LATHAM, Dr. J. F.R.S. critical analysis of his cases of tetanus in consequence of wounds, with particulars of the treatment, xxxi. 73; of his remarks on tumours which have occasionally been mistaken for diseases of the liver, 78; of his observations on certain symptoms occasionally denoting angina pectoris, 161.

LATHYRUS, botanical description and medicinal properties of the, xix. 77.

LAURENCE, Mr. John, his improved crutches for lame persons, described, xxxiv. 128.

LAUROCERASUS, its medicinal qualities, i. 503; its efficacy in repressing hæmorrhage, v. 500; mode of preparing the distilled water of, ix. 288; its properties traced to its containing prussic acid, xi. 93; observations on its effects on the animal system, xiv. 572.

LAUTH, Thomas, his elements of myology and syndesmology, iv. 177.

LAVA, hot, extraordinary change induced by, in metallic bodies, vi. 570.

LAVOISIER, defence of his theory relative to atmospherical air, i. 27; his works on the antiphlogistic system of chemistry the first which appeared on that subject, 174; remarks on his doctrine of phlogiston, 371; his hypothesis to account for the elasticity of aëriform fluids considered, v. 550.

LAWRENCE, Mr. William, his translation from the German of Blumenbach's Comparative Anatomy, announced, xvii. 200; analysis of his treatise on hernia, which gained the prize offered by the Royal College of Surgeons in 1806, xix. 85; his observations on a peculiar affection of the testis, critical remarks on, xx. 171; analysis of his observations on lithotomy, xxi. 415; his account of a child born without a brain, xxxiii. 152.

————, Mr. John, on the origin of the cow-pox, with remarks on the pox of swine, i. 114; symptoms of the latter, with the preventive means and remedies, described by him, 116; his general treatise on cattle, &c. reviewed, xiii. 564; his observations on vaccination, xvi. 214.

————, Mr. of Cirencester, on the influenza of 1803, x. 199.

LAZARETTO, floating, recommended in preference to those within land, xiii. 273; necessity of their establishment where infectious fevers prevail, xiv. 205; the earliest, its establishment in 1448 at Venice, together with that of the quarantine system, xviii. 114.

LEAD, as it is contained in various spring-waters, baneful effects of, on the human system, xi. 463; not to be readily detected, on account of the mode of its combination with the fluid, 571; its sedative qualities, xiii. 498; efficacy of the sugar of, in epilepsy, xiv. 10; xviii. 355.

LEAD, glass of, substituted for glass of antimony, by what tests to detect, xxiii. 439.

———, acetate of, combined with cold affusion, its efficacy in a case of cholera, iv 289; observations on, vii. 507; on the internal use of, viii. 209; its efficacy in curing epilepsy, xiv. 10; xvii. 355; its efficacy in several cases of hæmoptoe, xviii. 8; its efficacy in uterine hemorrhage, xix. 69; in a variety of diseases, particularly in profuse hæmorrhage, and in cases of salivation, xxii. 350; case of paralysis occasioned by the, xxvii. 100.

———, the poison of, cautions to those whose avocations may subject them to be influenced by, xxi. 526.

———, new, salts of, chemically analysed, xxix. 523.

LEADBEATER, Mr. his case of periodical spasm entirely subdued by the arsenical solution, xxv. 477.

LEASE, Mr. his case of tetanus, from a gun-shot wound, xxix. 100.

LEATH, Mr. description of his new lancet, provided with a guard, for dividing the inflamed vessels in ophthalmia, xxii. 42.

LECTURES, medical, &c. in London, notices of, ii. 191, 298; iii. 179, 377, 588; iv. 91, 281, 571; v. 103, 200; vi. 192, 284, 380; vii. 94, 191, 384, 575; viii 288, 383, 480; ix. 96, 292, 584; x. 285, 384, 480; xi. 95, 288, 480, 576; xii. 284, 287, 384; xiii. 94, 191, 288, 479, 576; xiv. 96, 284, 383; xv. 94, 198, 487; xvi. 285, 287, 383, 480, 576; xvii. 94, 199, 392, 487, 584; xviii. 290, 387; xix. 95, 288, 479, 576; xx. 194, 288, 385, 482; xxi. 87, 263, 351, 440; xxii. 87, 176, 262, 351, 528; xxiii. 86, 175, 263, 440, 528; xxiv. 88, 262, 344, 526; xxv. 90, 186, 368, 461, 547; xxvi. 254, 346, 423; xxvii. 78, 172, 257, 523; xxviii. 242, 338, 425, 517; xxix. 85, 174, 257, 433, 526; xxx. 174, 261, 349, 522; xxxi. 85, 170, 173, 433, 521; xxxii. 170, 258, 347, 526; xxxiii. 74, 163, 253, 429, 516; xxxiv. 172, 259; delivered in the University of Jena, in 1799, account of, i. 87; medical, in Columbia College, New York, summary of the, 83; on diet and regimen, critical analysis of, ii. 301, 393; on Animal Life, critical analysis of a Course of, iii. 184, 283; on the laws of animal nature, prospectus of a course of, 487; at the University of Glasgow, notices of, xii. 286; xiv. 283; xvi. 286; xx. 288; xxii. 261; xxiv. 262; xxv. 186; xxviii. 338; xxxi. 346; xxxiii. 258; xxxiv. 258; at the Anatomical Theatre, Bristol, notice of, xxv. 90; xxvi. 254, 346; a course of, on electrical and electro-chemical science, account of, xxv. 280; at Trinity College, Dublin, notice of the, xxx. 261; on the physiology of the brain, notice of a course of, xxxii. 82; in 1815, delivered before the Royal College of Surgeons of London, critical analysis of part of the, xxxiv. 65.

LEDUM PALUSTRE, its efficacy in the cure of cutaneous and other diseases, iv. 97.

LEE, Mr. of Philadelphia, his remarks on Dr. Mitchill's hypothesis relative to the nitric acid in a gaseous state, vii. 317.

———, Mr. Thomas, his facts and arguments demonstrative of the necessity of suspending the public opinion relative to vaccine inoculation, viii. 182.

———, Mr. of Bristol, on the influenza of 1803, x. 99.

———, Mr. of Spanish Town, Jamaica, his case of a remarkable and successful termination of scrotal hernia, xv. 381.

———, Mr. P. his note to the Editors of the Journal, on the effects of ammonia in preventing contagion, xxxi. 83.

LEECHES, details relative to a peculiar description of, which was swallowed and stopped in different parts of the throat, ix. 262; their great mortality in particular seasons, xii. 219; how such mortality is to be accounted for, 357; general observations on the mode of preserving them, and the temperature best adapted to them, 486; how best preserved, xiii. 76, 299; on the right management of, xiv. 186; simple mode of treating, xv. 198; the occasional injurious effects of, observations on, xix. 57; changes undergone by, before any particular alteration of the weather, xxiii. 212; mode of preserving, 213; directions in applying, ibid.; treatment of, after they are satiated with blood, 214; mode of treatment by which they are best preserved, and fit to be almost instantly re-applied, 322; case of a, situated in the back of the throat, behind the velum pendulum palati, 498; medicinal observations on their structure, xxvi. 408; efficacy of, in the fever incident to children, xxxi. 364; description of a substitute for, xxxiv. 61.

LEESE, Mr. Edward, on the existence and progressive stages of small-pox and measles in the same person, iv. 28; on vaccine inoculation, 337; his fatal case of natural small-pox subsequent to variolous inoculation, xiv. 251; his case of stricture of the urethra, with the appearances on dissection, xvi. 107.

———, Mr. L. of Copthall-court, his observations on vaccination, xii. 164.

LEESON, Mr. his observations on cataract, viii. 548; his case of semi-osseous tumour, x. 162.

———, Mr. B. jun. of Grantham, on croup, with the particulars of two cases treated by him, iv. 33; further account of the case of semiosseous tumour, xii. 465.

LEFEBURE, Professor, notice of his researches on the nervous fluid, vi. 175.

LEG, the Rev. Mr. notice of his contrivance for discharging the superfluous water from ponds, tanks, and reservoirs, in times of floods, xx. 383.

———, case of a compound fracture of the, successfully treated, iii. 549; artificial, invented by Mr. Potts, observations on the, vi. 547; singular case of extenuation in the, with the curative means employed, ix. 231; an ulcerated, successfully treated, x. 8; treated by the stimulating plan, xv. 217; observations on ulcers of the, with their treatment, xxiii. 432.

LEGS, on artificial, v. 493; vi. 445, 447, 547; œdematous, mode of reducing, xiii. 288; the calves of the, two cases of a singular affection of, xviii. 278.

LEGALLOIS, M. his experiments on animals, demonstrative of the influence of the nerves on the motions of the heart, xxvii. 375.

LE GROS, Mr. his account of a seaman, one side of whose face, having been scorched by gunpowder, became perfectly smooth, while the other was scarred as before with the small-pox, xix. 20.

LEIPZIG Michaelmas fair, in 1813, catalogue of medical books published at the, xxxi. 342.

LEMON, Mr. his case critically examined, in which a mass, resembling a placenta without a fœtus, was discharged from the womb, xxxiii. 333.

LEMONS, the acid of, or the citric acid, combined with the malic acid in the juice of lemons, in the sap of gooseberries, as well as in a variety of other fruits, i. 169; the expressed juice of, simple mode of concentrating, iii. 171.

LEMPRIERE, Mr. observations on the diseases of the army in Jamaica, i. 409; his report on the Walcheren fever, xxiii. 183.

LENTIN, M. on the nature of caries ossium, i. 294; on the nature of the falling star, ii. 298; case of hydrothorax, ix. 94.

LENTZ, Dr. notice of his mineralogical pocket-book, ii. 193.

LEONHARDI, M. on phlogiston and oxygen, ii. 83.

LEONTODON, botanical description and medicinal qualities of, xix. 166.

LEPROSY, utility of oxygenated pommade in, iii. 167; grecorum, case of, vi. 124; remarks on that of the middle ages, ix. 554; case of, cured by mercurial purgatives and blisters, xii. 404; mercurial, case of, xiii. 177; the utility of arsenic in, xv. 296; case of, successfully treated by sulphurated kali, xvii. 313; remarks on, xviii. 84; account of the diseases which have been thus termed, xxii. 407.

LEROY, M. observations on the use of hot flour as an application to erysipelas, i. 152.

LEROUX, M. case of fistula of the stomach, viii. 361; on the hyacinthus non scriptus, 476.

LESTES, a wind at Madeira, account of, xxxiv. 409.

LETTSOM, Dr. J. C. history of the tea-tree, ii. 192; on the cow-pox, iv. 567; his testimony before the House of Commons relative to cow-pox, vii. 494; on cow-pox, xvi. 181; on the efficacy of spirit-of-turpentine in cases of tænia, xxv. 167; biographical account of, xxxiv. 526.

LETTUCE, on the narcotic extract of, vi. 396.

LEUCOMA, observations on, viii. 329.

LEUCORRHŒA, on the utility of cantharides in, xv. 415; diagnosis of leucorrhœa and gonorrhœa, 416; utility of cantharides in, xvi. 78; remarks on, xxi. 34; xxxii. 239.

LEVANT, on the pestilential fever of the, viii. 449.

LEVANTERS, (winds,) observations on, xxxiv. 407.

LEVEILLE, M. on the use of muriate of barytes, ii. 469; on an extraordinary fœtus, 479; on the retina of the human eye; on a case of imperforate anus, ibid.

LEWEN, Meyer, Dr. on the state of medicine amongst the Hebrews, i. 436; on the medicinal effects of yeast, ii. 374.

LEWIS, Mr. case of enlarged clitoris, xxv. 236.

LICHEN ISLANDICUS, on its medical qualities, viii. 568; ix. 20, 292, 488; x. 379; xi. 90, 283; xii. 463; xvii. 255; xix. 34; xx. 160.

LICHTENBERG, Hr. his experiments with nitric acid, v. 176.

LIFE, treatise on the prolongation of, by Hufeland and Kant, i. 259, 493; review of Bichat's researches on, v. 476, 563; the result of organization, xx. 105; consists in motion, 108.—*See* VITALITY.

LIGATURE, remarks on the passing the thread through the centre of an artery, 340; use of it known to Celsus, Galen, and Paulus Ægineta, xiii. 391; remarks on Paré's claim to the introduction of its use, xiv. 40, 103; on the date of its use in hæmorrhage, 111; employed by the Greeks for aneurism, 112; Dr. Jones's experiments on its mode of action, xv. 463; xvi. 88; observations on its use, xvii. 192; xxiii. 253; on its application to wounded intestines, xxviii. 10.

LIGHT, the doctrines of Richter respecting it, i. 269, 271, 375; its influence on metals, iii. 91; Dr. Blackburn's opinion respecting it, 501; its influence on phosphorus, vi. 374; its distinctness from heat, viii. 573; a ready mode of producing it, 572; its effects compared with those of heat, ix. 83.

LIGHTNING, M. Claubrey's observations on the utility of alkalies in accidents from, iv. 370; some remarkable effects of in the human body, vi. 277; xxiv. 437.

LIGUSTRIUM, botanical description and medicinal qualities of, xii. 364.

LIMBS, on the means of supplying the loss of, v. 277; case of uncommon affection of, ix. 233; on injuries of the lower, x. 88.—*See* LEGS.

LIME, observations on its febrifuge qualities, i. 274; its utility in diabetes, vii. 66, 108; carbonate of, its utility in cancer, ix. 496; various chemical observations on, 493; its use in yellow fever, 100.

LIME-TREE, botanical account of, v. 394.

LINCK, Professor, observations respecting a pregnant mule, iv. 186.

LINCOLNSHIRE, its medical topography, viii. 221.

LIND, Dr. James, on the utility of opium in tetanus, vii. 125; on typhus fever, ix. 502; review of his treatise on the diseases of hot climates, xxi. 71.

LINUS, botanical description and medical qualities of, xiv. 62.

LIPSCOMB, Mr. on dyspnœa, iii. 520; review of his work in favour of small-pox, xv. 81.

LIQUIERE, M[me.] on the mechanism of the act of parturition, i. 193.

LIQUOR AMNII, case of early discharge of, viii. 250.

LITHOSPERMUM, botanical description and medicinal qualities of, xii. 124.

LITHOTOMY, description of M. Guerin's instrument for, viii. 385; account of Mr. Abernethy's gorget, ix. 393; case of, in which a pin formed the nucleus of the stone, 204; history of, xi. 3; on the position of the patient in the operation for, xv. 569; on the use of the knife in preference to the gorget, xxi. 415; observations on two instruments for, xxii. 207, 224, 339; xxiii. 238; remarks on the operation of, xxvi. 72, 285; various remarks on, xxviii. 496.

LITHONTRIPTICS, on the efficacy of muriatic acid as such, xv. 570.—*See* CALCULI.

LITTLE, Mr. on vaccination, xiv. 58.

LITTLEHALES, Dr. on the influenza, x. 386.

LIVER, on hæmorrhage from, iv. 67; general observations on the diseases of, viii. 238; case of extraordinary disease of, ix. 129; cases of abscess of, x. 1, 489; remarks on the diseases of, xi. 205; on the diseases of, in hot climates, xv. 87; reflections on its functions, xvi. 193, 289; case of abscess of, evacuated through the lungs, xvii. 500; reflections on its functions, 508; its influence on the rest of the system, 510; on its functions, xviii. 293; on inflammation of, 301; wound of, successfully treated by copious blood-letting, 333; rupture of, from a blow, 391; on the economy of, 426; case of tuberculated, 485; reflections on its functions, xx. 339; disease of, connected with intemperance and intermittent fever, xxii. 191; on inflammation of, as it occurs in India, and on the use of mercury for it, 415; on its functions, 421; case of inflammation of, xxiii. 226; case of extensive suppuration of, terminating favourably, xxv. 164; disease of, the cause of hypochondriasis, xxv. 212; synonyms of the word in various languages, 312; case of rupture of, from external injury, 335; cases of abscess of, xxvii. 94, 278; observations on the pathological anatomy of, xxviii. 509; on chronic inflammation of, 95, 279, 300; effects of disease of, on the cerebral functions, 213; utility of arsenic in diseases of, xxx. 22; on inflammation of, 190; case of abscess of, xxxi. 8; remarkable disease of, 161, 353; treatise on its diseases in general, 425; utility of mercury in chronic affections of, 447; abscess of, consequent on injury of the head, xxxii. 119; extensive abscess of, 370; on its functions in the fœtus, xxxiii. 224; morbid anatomy of, in general, xxxiv. 71.

LIVERPOOL, report of diseases at, xv. 388.

LOBELIA, its utility in venereal and other diseases, xii. 516.

LOBSTEIN, Professor, on the nutrition of the fœtus, ix. 255; xv. 229.

LOCHIÆ, on the cessation of, x. 13.

LODGE, Mr. observations on asphyxia, xiv. 150.

LOFFLER, Dr. case of inversion of the uterus, xi. 207.

LOMBART, M. on the application of cold to strangulated hernia, ii. 46; distortion caused by the use of stays, 47; case of abscess of the liver, 48; on fistula of the anus, 154; on cholera morbus, 155.

LOMBARDY, discovery of cow-pox in, vii. 172.

LOMAS, Mr. on influenza, x. 198.

LONGEVITY, Sir John Sinclair's queries respecting it, viii. 381; observations on, xiii. 527, 81.

LONICERA, botanical description and medicinal qualities of, xii. 130.

LOTUS, botanical description and medical qualities of, xix. 163.

LOUDHOUTER, Mr. case of hydrophobia, xx. 327.

LOUPING DISEASE, (the jumping ague of Scotland,) description of, xxvii. 486.

LOWE, Mr. on the use of musk and ammonia, ii. 342; case of hydrothorax, ix. 417; mode of making casts of the ear, 551; x. 357; observations on hydrophobia, xii. 436; on ring-worm of the head, xiii. 24; case of effects of some poisons, xxviii. 182.

LOWER, Dr. on restriction of liquids in some diseases of the lungs, i. 20.

LOWTZ, Professor, his experiments on the production of artificial cold, i. 196; his experiments with vegetable acids, ii. 81; his mode of preparing vinegar, v. 395; on the decoloration of vegetables, viii. 471.

LOWNDES, Mr. on medical electricity, xvii. 257.

LOWRY, Dr. observations on the cure of gout, xiii. 349.

LUBBOCK, Dr. on the chemical doctrines of Mayow, i. 220, 418; on cow-pox, v. 23; on diabetes, 56; vii. 107; cases of catalepsy, 105; xiii. 180.

LUCAS, Mr. James, of Coninsbrough, case of schirrous tumour in the breast, vii. 49; case of singular extenuation of the leg, ix. 233.

LUCIUS, M. on the influence of light on metals, iii. 91.

LUMBAGO, in females, observations on, xxi. 34.

LUMBAR ABSCESS.—*See* ABSCESS.

LUMINOUS ANIMALS, observations on, xxv. 409, 502.

LUNAR INFLUENCE, case of apparent, in epilepsy, xviii. 356.—*See* SOL-LUNAR INFLUENCE.

LUNATICS, remarks on the Act relative to, xxxii. 4.

LUNGS, on stony concretions coughed-up from, x. 327; remarks on their functions, xi. 46; physiological observations on, xii. 239; on inflammation of, xvi. 481; xvii. 158, 580; on the diseases of, xviii. 9; on inflammation of, 57; the influence of deleterious gases on, xix. 203; severe cases of inflammation of, 248; on condensation of, xx. 178; on the treatment of inflammation of, 242; xxi. 82; xxii. 84; remarks on diseases which simulate those of the heart, xxix. 55; on the black matter found in, 506; on inflammation of, xxx. 190; xxxii. 171, 283.—*See* CONSUMPTION.

LUNN, M. on the removal of schirrous parotid glands, xxii. 221.

LUSCOMBE, on styptic applications, viii. 197; on influenza, x. 127; case of fever, xiii. 348.

LUSSAC M. Gay, his experiments on the torpedo, xv. 295.

LUXMOORE, description of his bag-truss, xi. 304.

LYALL, Mr. on cupping, xxii. 388; on purulent ophthalmy, xxiii. 154; on the staphyloma, xxv. 359; on the effluvium of fraxinella, 520.

LYCOPODIUM, botanical description and medical qualities of, xix. 540.

LYTTA VITTATA, on its vesicating qualities, xxiii. 323.

M.

MACARTNEY, Dr. James, observations on luminous animals, xxv. 409, 512.

MAC DOWELL, on the treatment of burns by cold water, xvii. 320; case of phthisis cured by mercury, xix. 20.

MAC GREGOR, Dr. James, medical sketches of India and Egypt, xii. 77; his opinion on contagion, xxi. 241.

MACHELL, Mr. T. description of his annular saw, xxxiii. 189.

MACKIE, Dr. history of a case of ossified uterus, iii. 422.

MACCULLOCH, Mr. on blood-letting in fever, xxvi. 108.

MACLEAN, Dr. observations on the medicinal qualities of digitalis, ii. 177; iii. 150; iv. 127, 237; observations on paracentesis of the abdomen during pregnancy, vii. 193; review of his treatise on hydrothorax, xxiii. 330; cases of hydrothorax, 276.

MAC LEOD, Mr. observations on yellow fever, xxxiii. 5.

MAC MULLIN, Mr. on mercurial erythema, xv. 280.

MAC WHIRTER, Dr. case of cancer, vii. 51.

MADREPORIT, analysis of the, ix. 476.

MAERKER, Dr. case of obstruction in the fauces, xi. 190.

MAGENDIE, Dr. observations on the organs of absorption, xxv. 44; on the use of the epiglottis, xxxi. 318; experiments on the act of vomiting, xxxii. 419.

MAGENNIS, Dr. observations on dropsy, iv. 212; on epilepsy, 417; remarks on apoplexy, ix. 320; on the influenza, x. 122.

MAGNESIA, remarks on its preparation, iii. 87, 170; observations on its utility in calculous disorders, xxiii. 477; xxiv. 203; xxx. 324.

MAHON, Dr. remarks on medical jurisprudence, ix. 69; on the communication of the venereal disease from mother to child, xx. 560.

MAIDEN, Mr. case of recovery from a severe wound in the chest, xxix. 68.

MAINGAULT, Dr. experiments relative to the act of vomiting, xxxii. 517.

MALE, Dr. case of suppuration of the liver, xii. 497; case of extraordinary appearance of the stomach, xiii. 164.

MAL D'ALEPPO, account of the, xxxi. 232, 372.

MALTA, account of the plague of, xxxiii. 477.

MAMMOTH, observations on the, xv. 486; xviii. 95; xix. 287.

MAMMUT OBIOTICUM, observations on the bones of, iv. 186.

MAN, philosophical, physiological, and political, treatise on, xviii. 178; observations on the natural history of, xxii. 8.

MANN, Abbé, his extraordinary case, xi. 217.

MAN-MIDWIFE, on the origin of the term, xxviii. 100.

MANSCEY, M. on the division of the symphisis pubis, ix. 57.

MANSFIELD, Mr. observations on the conversion of animal matter into adipocire, iii. 10.

MANSFORD, Mr. observations on animal heat, xxix. 441.

MANTAL, Mr. observations on gout, xii. 500; xiii. 144, 437.

MANUFACTURES, enquiries respecting those prejudicial to health, xv. 100.

MAPLESON, Mr. review of his treacise on cupping, xxxiv. 77.

MARCET, history of a case of diabetes, ii. 209; his correspondence with Dr. Jenner, ix. 462; case of a blue girl, xiv. 471; on the use of the white oxide of bismuth, xv. 563; case of hydrophobia, xxii. 157; observations on the blood in diabetes, xxvi. 467; on a new mode of treating rheumatism, xxx. 235.

MARCUS, Dr. various pathological remarks, ix. 268; on intermittent fevers, 304; account of his galvanic experiments, x. 333.

MARIUS, Bishop of Lausanne in the sixth century, acquainted with cow-pox, vi. 379.

MARQUAIS, Dr. experiments relative to the act of vomiting, xxxii. 518.

MARSHALL, Dr. on cow-pox, iv. 386, 544; vii. 480; on the influenza, x. 405; on vaccination, xi. 39; biography of, xxxiii. 60, 109.

————, Mr. case of concussion of the brain, xxix. 460; case of production of premature parturition, xxxii. 100.

————, Mr. H. case of hydrophobia, xxxii. 217.

MARSILLAC, M. on a new mode of inoculation, ii. 468.

MARSON, Mr. observations on the use of the catheter, x. 174; on the furniture of the chamber of a sick person, 175; on church-yard infection, 177; on the delivery of the placenta, 433; xi. 328; on vaccination, xvi. 70; on the treatment of burns, xvii. 130; on vaccination, 131; xviii. 70.

MARTEN, Dr. on the medical application of galvanism, x. 330.

MARTIN, Mr. observations on paracentesis of the abdomen, xiv. 418; case of imperforate anus, xviii. 297; on the treatment of burns and scalds, xix. 17; on cold affusion in fever, xx. 51.

MARTINEAU, M. on cold affusion in fever, iii. 51.

MARYAN, remarks on hydrophobia, xxii. 34; remarks on his opinions, xxiv. 46.

MASCAGNI, observations on calculous concretions in the urine, iv. 467.

MATERIA MEDICA, notice of Professor Arnemann's system of, i. 29; of Bartoni, ibid.; remarks on medicines termed febrifuge, iv. 51; treatises on the mode of agency of the, vii. 80; viii. 90; xix. 365; on the difficulty of ascertaining the qualities of the, ix. 19; remarks on the doses of active articles of the, xviii. 55; those used by Hippocrates, xii. 470; those comprised in Dr. Swediaur's Pharmacopœia, 560; theory of the effects of, xiv. 372; xix. 365; xx. 12; various observations on, xxiii. 424.

MATON, Dr. on the medicinal qualities of the ledum palustre, iv. 97; case of catalepsy, vii. 30; case of superfœtation, xxxi. 150.

MAURICE, Mr. on the coincidence of diseases, ix. 38.

MAXILLARY SINUS, case of inflammation in the, 423.

MAY, Dr. observations on the influenza, x. 291.

MAYOW, Dr. remarks on his chemical discoveries, iii. 335.

————, Mr. Charles, case of ruptured uterus, xxx. 444.

MEAD, Dr. his distinction of the varieties of tympanitis, vii. 232; his letters to Dr. Deacon of Manchester, x. 48; application made to, by the ministry, respecting a code of quarantine laws, xviii. 120.

MEADOW RUE-WEED, botanical description of, xvii. 372.

MEADOW-SAFFRON.—*See* COLCHICUM AUTUMNALE.

MEADOW-SWEET, botanical description and medicinal qualities of, xvi. 171.

MEASE, Dr. on digitalis, i. 56; on hydrophobia, viii. 88; on the efficacy of mercury in typhus and in tetanus, and on that of the cold-bath in fever, xix. 109.

MEASLES, examples of their co-existence with small-pox, iii. 572; iv. 28; x. 67; xiv. 142; some observations on the dissemination of, vii. 316; on their epidemic character, viii. 28; x. 470; xi. 309; on the popular opinion in the north of England that this disease originates in swine, 432; on inoculation of, 459; xix. 153; xxvii. 118; critical analysis of a treatise on, xiv. 362; the herb meadow-sweet recommended as a remedy, xvi. 171; great prevalence of, in London, in the winter of 1807, xviii. 572; xix. 91; and in Edinburgh at the same time, 185, 279, 471; on diseases subsequent to, xix. 133; morbid anatomy of fatal cases of, 154; epidemical in 1808, xxi. 359; remarks on the treatment of, xxiii. 348; the late fatality of, supposed to be influenced by vaccination, xxxi. 66; this opinion disputed, 273; case of secondary, xxxii. 256.

MECCA, balm of, the opinions of the Asiatics on its medical qualities, i. 33; botanical description of the plant producing it, 33, 34.

MEDICAL SOCIETIES.—*See* SOCIETIES.

MEDICINA NAUTICA, treatise on, ix. 185.—*See* NAVY.

MEDICINE, remarks on clinical, i. 176; historical enquiry into the state of amongst the Hebrews, 436; review of Kaninfeld's Institutes of, ii. 331; Swedish, annals of, 399, 498; Edinburgh, annals of, for 1799, iii. 482; recent history of, 578; xii. 419; xviii. 253; remarks on the philosophy of, v. 287; xiv. 3, 74, 76; observations on the practice of, in the navy, 36; remarks on the practice of, xviii. 394; historical sketch of the progress of, in 1806, 1; in 1807, xx. 1; in 1808, xxii. 1, 105; in 1809, xxiv. 1; in 1810 and 1811, xxvi. 1, and xxvii. 1; in 1812, xxvii. 1, and xxix. 1; in 1818, xxx. 1; remarks on the danger of popular treatises on, xxviii. 18; state of, in India, xxx. 220; state of, in Italy, in 1812, xxxi. 81; state of, in Spain, at a late period, 285; state of, in China, xxxiii. 247; state of, in France, in 1814, 298; state of, in Sicily, xxxiv. 186; review of Cabanis's work on the revolutions of, xiv. 73; on theory in, xvii. 66; on the practice of, in America, xix. 414; sketch of a natural system of, xx. 39; on education for the profession of, xxii. 302; xxiii. 438; xxiv. 303; remarks on the study of, xxvi. 68; on schools for the study of, xxviii. 461; observations on the state of it, in India, xxx. 219; state of, amongst the Patagonians, and other American tribes, xxxi. 304; state of, in France, in 1815, xxxiv. 478.

MEDICINE, on reform of the state of, in England, discussions on, viii. 107; xv. 93, 258, 551; xvi. 279, 345, 455; xvii. 28, 195; xviii. 3, 185; xix. 61; xxii. 328, 465, 489; xxiii. 48, 63, 134, 189, 209; xxiv. 173, 494; xxv. 111, 216, 402, 492; xxvi. 2, 123; xxviii. 38, 403; xxix. 1, 114, 129, 165, 201, 275, 306, 445, 456, 467; xxx. 1, 173, 177, 265, 307, 385, 452, 467, 478; xxxi. 91, 229, 321; xxxii. 322; xxxiv. 186.

MEDICAL STATISTICS, on the health of manufacturers, xvi. 100; general account of the population of England, Scotland, and Ireland, xxvii. 169. —*See* MEDICAL TOPOGRAPHY; DISEASES, REPORTS OF; *and* MORTALITY, BILLS OF.

MEDICAL JURISPRUDENCE, remarks on, ix. 69; xix. 237; xxi. 336, 399, 426; xxii. 118; xxvi. 148; xxxii. 4, 122; review of Dr. Farre's Elements of, 326.

MEDICAL TOPOGRAPHY, notice of Dr. Michell's outlines of, i. 254; of India, and Upper Egypt, xii. 77; of Madeira, xvii. 291; xix. 19; of Malta, observations tending to shew the utility of a general one of Great Britain, xxi. 1, 91; xxii. 106; xxv. 64, 425; of Mexico, xxv. 239; of Nottinghamshire, xxx. 98; of Tripoli, xxxi. 478.

MEDLAR-TREE, botanical description and medicinal qualities of, xvi. 169.

MEDULLA, observations on its use in regulating the growth of bones, xx. 286.

MEDULLA-OBLONGATA, remarks on injury of the, xxiv. 57.—*See* BRAIN.

MEDUSA, natural history of the, xxii, 526.

MEGLIN, M. on tic douloureux, xxxi. 380.

MELÆNA, case of, xxii. 180.

MELANCHOLY.—*See* MENTAL ALIENATION.

MELHERE, M. on the causes of irritability, x. 25.

MELHURST, Mr. on the influenza of 1803, x. 304.

MELKSHAM, description of the mineral water of, xxx. 299, 383.

MELIA AZEDARACH, its efficacy against worms, viii. 429.

MELILOT, botanical description and medicinal qualities of, xix. 162.

MELLOR, Mr. history of a remarkable case of paralysis, xxvi. 153; case of hæmorrhœa petechialis, 154.

MELOGRANI, L'Abbé, description of his blow-pipe, xvii. 196.

MELOS VESICATORIUS, observations on its qualities, xviii. 22.

MELVILLE, account of his experiments with a new diving-machine, xxxii. 522.

MEMBRANES, general observations on them, and on their diseases, vi. 449; xvi. 152; on inflammation of those of the bronchiæ, xx. 78; observations on inflammation of the mucous and serous, xxvii. 354, 357, 361; on inflammation of the mucous membrane of the intestines, xxxiv. 18.

———, of the tympanum, observations on puncture of, iv. 570.

MEMMINGER, Dr. on the use of alkali in pertussis, ix. 176.

MEMORY, remarkable instance of loss of, from hemiplegia, vi. 277.

MENORRHAGIA, observations on the treatment of, xix. 102; remarks on the causes and treatment of, xxi. 31.

MENSTRUATION, case of premature, vii. 1; remarks on Cullen's doctrine of, xxi. 30; history of a case of vicarious, xxv. 24.

MENTAL ALIENATION, remarks on, by Dr. Johnstone, iv. 260; by Mr. Crowther, 508; remarkable cases of, viii. 311; x. 50; treatise on melancholy, xxi. 495; observations on, xxvii. 512.—*See* INSANITY *and* DELIRIUM.

MENTHA, botanical description of, xvii. 558.

MERAT, Dr. cases of tubercles in the brain, xvi. 470.

MERCURY, observations on its use in hydrocephalus, v. 450; its efficacy in inflammatory affections, vii. 315; xxi. 257; xxii. 462; effects of in ascites and anarsarca, 401; on its deleterious effects, viii. 9; its utility in yellow fever, ix. 98; the effects of the oxy-muriate of, in gonorrhœa, x. 283; remarks on its use in venereal diseases, xiii. 395, 549; the erythema consequent on its use, 177; xv. 280; xxvi. 341; xxviii. 61; utility of, in hydrophobia, when conjoined with bleeding, xiv. 122, 538; its utility in liver complaints, xv. 87; experiments on frictions with, 274; on fumigation with, ibid.; remarks on its agency on the system, 576; xvi. 415; xviii. 192; xxv. 62; xxvi. 27; xxviii. 508; xxix. 207; its good effects in phthisis, xviii. 324; xix. 20, 324, 331; xxi. 117; its diuretic effects, xviii. 331; successful use of in typhus and in tetanus, xix. 109; remarks on its use in typhus, 315; failure of, in tetanus, 446; xxii. 185; on the danger attending its use in hepatitis, 416; deleterious influence of, in scrofula, 494; its utility in tetanus doubted, 520; its utility in hydrophobia, xxiv. 29; case of its deleterious influence on the brain, 52; its utility in diseases of hot climates, 461; its efficacy in acute rheumatism, xxvi. 277; on its utility in dysentery, xxviii. 67; its effects in its native state, xxix. 207; various remarks on its efficacy, xxx. 100, 446; utility of, in diseases of the liver, xxxi. 447; its beneficial agency in two cases of severe affection of the brain, 497; treatise on its utility in febrile diseases, xxxiv. 323; remarks on its poisonous effects, xxx. 342; its action and effects in the cure of the venereal disease, 446; its utility in some affections of the brain, xxxi. 497; remarks on its utility in fever, xxxiv. 323.—*See* CALOMEL.

————, *chemical preparations of:* remarks on fulminating mercury, viii. 190; efficacy of the oxy-muriate of, in preserving animal matter from putrefaction, 479; mode of preparing the red oxide of, 573; and the submuriate of, ix. 81; on its combinations with silver, xxv. 457; general observations on, xxvi. 27.

————, (the plant *chenopodium,)* botanical description and medical qualities of, xii. 227; xv. 71.

MERMAID, account of a supposed one that appeared on the coast of Scotland, xxvii. 76; account of that at Haerlem, 382.

MERRIMAN, Dr. observations on cow-pox, xiv. 174; xv. 231; on retroversion of the uterus, xvi. 385; remarks on Dr. Dewee's cases of midwifery, xviii. 417; histories of two cases of inversion of the uterus, xxi. 63; case of suppression of urine after parturition, xxiv. 360; dissertation on retroversion of the uterus, 406; xxvi. 33; case of monstrosity, xxv. 279; on the origin of the use of men-midwives, xxviii. 100; his synopsis of the varieties of difficult parturition, xxix. 421; case of difficult parturition from dropsy of the ovary, xxx. 163.

MESEMBRYANTHUM, *Pinnatifidum*, botanical history of, ii. 296.

————————, *crystallinum*, its utility in diseases of the urinary organs, vi. 371.

MESTIZOES, (natives of Mexico,) observations on the physical strength of the, xxv. 246.

METALS, remarks on their mode of agency on the animal economy, xi. 287; experiments on several of them with the galvanic battery, xx. 95.

METASTASIS, of disease, reflections on, xxiv. 178.

METEOR, that which appeared in the year 1719, considered as the cause of the malignant fever then epidemic, xxii. 286; account of some which fell in India, xxv. 457, in Iceland, 545, in France, xxvii. 82.

METFORD, Dr. observations on the influenza of 1803, x. 108.

METTERNICH, Dr. account of his successful exhibition of the rhododendrum chrysanthum in gout, xxvi. 508.

MEXICO, observations on the diseases prevalent in, and on the causes that arrest the progress of population in, xxv. 239; remarks on the climate of, and on its influence on man, xxvi. 10.

MEYER, Dr. account of his experiments to prove that iron enters into the composition of the chyle, iv. 186; remarks on his treatise termed the Syphilitic Physician, xxvii. 137.

MEYLER, Dr. on the use of carbonate of magnesia in calculous disorders, xxxiv. 445.

MEZEREUM, (bark of,) recommended as a local application for producing issues, v. 278; botanical description and medicinal qualities of, xiv. 548.

MIASMA, *marsh*, remarks on its morbific agency, xvii. 447; xviii. 86, 288, 473; xix. 113, 199; on its influence in the production of intermittent fever, xxi. 92; xxvii. 37; xxx. 15; its mode of agency, xxiii. 350, 470; xxiv. 365, 422.—*See* CONTAGION.

MICHAELLIS, Dr. remarks on a peculiar tumour found in the head of newborn infants, iv. 445; on the arrangement of field-hospitals, vi. 563; treatise on puerperal fever, viii. 433, 510.

MICHAUX, M. observations on the cultivation of jalap, xxi. 398.

MIDWIFERY, the London practice of, xi. 91; some remarkable cases in, with remarks, xviii. 337, 417; xix. 178; account of the early practitioners of, in England, xxvii. 117; practice of, among a tribe of South American Indians, xxxi. 305.—*See* LABOUR *and* DELIVERY.

MILDEW, observations on its nature, iv. 372.

MILK, on the acid contained in, xix. 124; chemical analysis of, 132.

MILK-WORT, botanical description and medicinal qualities of, xix. 72.

MILLER, Dr. Edward, observations on the effects of abstinence on the approach of acute diseases, i. 43; on cutaneous perspiration, 287; on the nature and treatment of febrile diseases, ii. 373; on yellow fever, ix. 103, 302; on sea-sickness, xix. 295, 422; on the epidemic fever of New York, xvii. 97; xx. 29.

MILLETT, M. R. O. case of gout, x. 254; on diarrhœa, xii. 51.

MILLINGTON, remarks on small-pox subsequent to vaccination, xvii. 233.

MILLS, Mr. case of uterine hydatids, xi. 447.

MILNE, Dr. notice of his Botanical Dictionary, xiii. 564.

MILWARD, Dr. memoirs relative to, xxvii. 69.

MIND, its influence on the organs, xiv. 16; and on the functions especially, xvi. 92; xxii. 463; query whether it be influenced by the physical condition of the head, xxix. 48.—*See* GALL *and* SPURZHEIM.

MINERALOGY, cabinets relative to, prepared at Leipzig, i. 87; treatise on, vii. 471; of Cornwall, xxix. 247; remarks on fossil remains, xxv. 519.

MINERALS, on the analysis of, vi. 373; on those occupying the fissures in the surface of the earth, xxviii. 75; vegetables formed in some species of, xxix. 430.

MINES, observations on the temperatures of, xvi. 192; deleterious influence of the working of, on the animal economy, xxv. 245; on the working of, 247.

MINORCA, observations shewing the yellow fever to be indigenous, in, xvii. 125.

MIRABEAU, Count, account of his fatal sickness, xxxi. 221.

MIRBEL, M. brief account of his observations on plants, vii. 557.

MIRZA, Mehredy Ali Khan, account of the practice of vaccination by a sect of Hindoos at Benares, xiii. 574.

MISLETOE, botanical description of, xii. 32; its utility in epilepsy, xviii. 17.

MITCHELL, Dr. John, case of exfoliation of the sternum, xxxiii. 8.

————, Dr. George, observations on the efficacy of lead in uterine hæmorrhage, xix. 69.

————, Mr. case of gout, xxvi. 244.

MITCHILL, Dr. Samuel, of New York, chemical remarks on manures, i. 45; observations on medical topography, 254; on the chemical discoveries of Mayow, 298; on septic fluids, ii. 180, 183, 380, 383, 470; ix. 304; on the perspirable fluid of animal bodies, iv. 9, 109; on the nitric acid, 24; commentary on Hoffmann's essay on the dissimilarity of fixed alkaline salts, xi. 404; abstract of his lectures on the constitution of the atmosphere, xxii. 177; account of his own case of croup, xxvii. 213.

MOFFAT, account of the mineral waters of, ii. 354.

MOFFATT, Mr. on animal chemistry, vi. 416.

MOGALLA, Dr. his opinions on cow-pox, xiv. 233.

MOISES, Dr. case of vomiting of pins and needles, vii. 17; on the efficacy of acetous affusion in fever, 337.

MOLLITIES OSSIUM.—*See* BONES.

MOLWITZ, Mr. account of his metallic brush, vi. 91; on the local application of hepatic gas, 92.

MONJENOT, Dr. observations on vaccination, xi. 323.

MONGERET, M. on the application of moxa to the sacrum in cases of incontinence of urine, xxxii. 346.

MONGIARDINI, Dr. on the application of galvanism to the cure of disease, xvii. 190.

MONNOT, M. on the extraction of the cataract, xi. 70.

MONRO, Dr. Alexander, jun. case of hepatitis, xiv. 469.

MONS, J. B. Van, on the purification of air in the chambers of the sick, i. 245; on the preparation of magnesia, ii. 87; chemical observations, iv. 469, 470; on the medicinal qualities of the toxicodendron, vi. 272.

MONSTROSITY, remarkable cases of, i. 224; ii. 226; iii. 138, 397, 402, iv. 189; vi. 94; xii. 13, 190; xiii. 575; xxiv. 61; case of an acephalous fœtus, vii. 385; viii. 382; cases of nævi materni, xv. 249; remarkable instances of, xxiv. 61; xxv. 277, 279, 494; reflexions on the mal-formations that are to be considered such, xxxii. 331; xxxiii. 157.

MONTAIN, Dr. observations on apoplexy, xxvii. 151.

MONTEGRE, Dr. account of his experiments on digestion, xxxii. 257; xxxiii. 420; observations on the lumbrici, xxxii. 347.

MONTROSE, Dr. case of diseased knee, xvii. 257.

MOODIE, Dr. case of abscess of the breast, x. 414; on the influenza of 1803, xi. 488; case of aneurism of the aorta, xi. 305; case of the bite of a viper, 481; observations on the poison of serpents, 483; case of extensive suppuration, xii. 252.

MOON, influence of.—*See* SOLAR *and* LUNAR INFLUENCE.

MOORE, Mr. S. on the vascularity of the teeth, x. 360.

———, Dr. F. observations on phlegmasia dolens, xxxii. 187.

———, Mr. James, case of ulceration of the stomach, iii. 511; on vaccination, xv. 283; xvi. 29, 477; on gouty concretions, xxii. 155; letter to Jones, on the Eau Medicinale d'Husson, xxvi. 224; on the history of small-pox, xxxiv. 121.

———, Anne, (the fasting-woman of Tutbury,) particulars of her case, xxi. 61; xxiv. 309; xxix. 109, 408, 469; xxx. 187.

MORBUS CARDIACUS, similarity of ancient and modern opinions respecting it, xv. 377.

——— SACER, case of, xv. 126.

MOREAU, Dr. on caries of the joints, x. 557.

MORGAN, Mr. John, case of cataract, viii. 215; on vaccination, xi. 41; xvii. 312.

———, Mr. Charles, observations on purgative medicines, xvii. 470; observations on deafness, xxxi. 379.

MORLEY, Mr. observations on cataract, ix. 172.

MOROMASTRIX, observations on the letter of a Derbyshire practitioner, xviii. 156; reply, 309.

MORRISON, Mr. of Dublin, remarks on the Dublin Pharmacopœia, xviii. 540; observations on the purulent ophthalmia of infants, xx. 57.

———, Dr. of Wolverhampton, on the influenza of 1803, x. 222.

MORTALITY, bills of, xii. 94, 190; xiii. 92; xiv. 382; xv. 200; xxi. 522; xxiii. 87; xxvi. 128.

MORTIFICATION. — *See* GANGRENE.

MORTON, Mr. J. case of ruptured spleen and liver from external injury, xxvi. 337.

MORVEAU, M. Guyton de, on the purification of infected air, viii. 185, 529; xiv. 382; observations on the use of carbonate of potash in diseases of the urinary organs, xxiv. 45; on the coloration of glass, xxv. 363.

MOSELY, Dr. his evidence before the House of Commons respecting cow-pox, viii. 141; treatise on the lues bovilla, xiii. 466; extracts from his address to the Rev. Rowland Hill, xvii. 333; case of hydrophobia, xviii. 550.

MOSES, (the Jewish lawgiver,) observations on his description of leprosy, xiii. 36.

MOSSES, of Sweden, systematic arrangement of, v. 370.

MOSSMANN, Dr. on the effects of digitalis in phthisis, ii. 238; iii. 311; x. 308; xx. 328; xxi. 325; on the benefit derived from cold water in scarlatina anginosa, iii. 556; remarks on fever, v. 129; observations on apoplexy, vii. 205; ix. 409; on the influenza of 1803, x. 387.

MOTION, *animal*, the cause of, x. 253; *muscular*, Croonian lecture in the year 1807, xvii. 93; in 1810, xxv. 180; observations on the organs of, xviii. 324. —*See* MUSCLES.

MOTTLES, Mr. J. C. observations on the qualities of tea, xxiv. 265.

MOXA, efficacy of, in cases of loss of speech after fever, ix. 289; and applied to the sacrum for incontinence of urine, xxxii. 346; in head-ache, xxxiii. 426; use of it by the Laplanders, xii. 35; and by the Japanese, xix. 357.

MUCUS, animal, chemical analysis of, xv. 281; tests of, xiv. 265.

MUDD, Mr. case of extirpation of the eye, xvii. 187.

MUDIE, Mr. case of the efficacy of nitrate of silver, in the cure of epilepsy, ii. 270.

MUFFIN, M. account of his experiments on sulphur and phosphorus, ii. 185.

MUG-WORT, (*artemisia*) botanical description and medicinal qualities of, xix. 357.

MULBERRY, *white*, (*morus alba*, &c.) discovery of a new acid in, xi. 288; xii. 92.

MULE, case of pregnancy of a, iv. 186.

MULOT, M. case of mal-formation of a fœtus, ix. 461.

MUMPS, remarkable case of, xxxi. 306.

MUNT, Dr. account of the radesyge of Norway, vi. 13.

MURPHY, remarks on his natural history of the human teeth, xxxi. 302.

MURRAY, Dr. John, of Edinburgh, account of his experiments on the power of fluids to conduct caloric, vii, 480.

———, Dr. T. A. remarks on the prevention of contagious fever in cities, v. 290.

———, Mr. John, review of his elements of materia medica, xi. 562; remarks on the efficacy of stramonium in the cure of mania, xxv. 504.

———, Mr. Charles, observations on small-pox and vaccination, xxxviii. 180.

MURSINNA, Hr. observations on tetanus, vi. 280; xvi. 473.

MUSCICAPA, observations on some species of that plant, xxvii. 388.

MUSCLE, the, cases of poisoning from it, used as aliment, xxi. 8.

MUSCLES, account of the conversion of them into fat, iii. 10; xvi. 303; remarks on a classification of them, iv. 71; on the galvanic influence on the, x. 53; xiii. 374; on the obstacles they offer to the reduction of luxation of the joints, xix. 289; remarks on the motion of, xxii. 5; xxiv. 485; xxv. 180; on those of the bladder, xxvii. 381; account of some unusual formation of the, xxviii. 505; observations on muscular motion, xxxi. 168, 288.

MUSCÆ VOLITANTES, (optical phenomenon,) remarks on, xxxiii. 411.

MUSHROOM, (*agaricus campestris,*) on the cause of its action as a poison, xv. 247; cases of poisoning by, xx. 457, 567.—*See* AGARICUS.

MUSK, observations on its medicinal qualities, ii. 342; efficacy in checking the progress of mortification, ix. 203; in tetanus, xviii. 401; in scarlatina, xxi. 257; remarks on its introduction to use as a medicine, xxiv. 236.

———, *artificial,* observations on its efficacy in hooping-cough, i. 177; v. 137; various observations on its medicinal qualities, iv. 234; v. 544.

MUSSIN PUSHKIN, Count, on the crystallization of platina, xiv. 373.

MUSTARD, botanical description and medicinal qualities of, xviii. 368; account of the composition termed the essence of, xiii. 502.

MUTER, Dr. remarks on the cataract and artificial pupil, xxvii. 318.

MYOLOGY, review of a treatise on, iv. 177.

MYRRH, observations on its medicinal qualities, viii. 48.

MYRTLE-FLAG, (*acorus,*) botanical description and medicinal qualities of, xiv. 67.

N.

NACQUART, M. case of organic lesion of the intestines, xxii. 150.

NÆVI MATERNI, general observations on, xv. 349.

NAMLET, Mr. observations on the diversity between small-pox and cow-pox, xv. 219.

NAPLES, remarks on the climate of xi. 284.

NAPTHA VITRIOLI, Prof. Hufeland's experiments with, vi. 378; its preparation described, viii. 189.

NARTHESIUM, botanical description and medicinal qualities of, xiv. 65.

NATRON, chemical analysis of the Egyptian and radiant, ix. 474.

NATURAL HISTORY, notice of the essays of the society of, at Berlin, iii. 284; of Paris, 272; remarks on a new elementary doctrine of, v. 369; account of the *Naturbistoriche fragmenta* of Prof. Fischer, vii. 372; various observations and reflections on, xx. 11; of the Zealand sheep, xxvii. 216; various observations and reflections on, 85, 173, 261, 341, 349; xxv. 189, 285, 372, 463, 551; xxvi. 15, 85, 173, 261, 349, 427, 515; review of a treatise on the study of, xxix. 501; xxx. 429; xxxiii. 308, 396; general abstract of, xxxii. 177.

NAUCHE, Dr. review of his treatise on diseases of the bladder and urinary passage, xxxi. 241; xxxii. 155.

NAUSEA, observations on the efficacy of acidulated minerals in that attending pregnancy, i. 284.—*See* VOMITING.

NAVALIS, Mr. jun. remarks on the treatment of burns and scalds, v. 236.

NAVIGATORS, review of a medical directory for, x. 83.

NAVY, observations on the regulations for the appointment of medical officers in the, xvii. 250; xviii. 60; xxii. 373; sketch of a plan for improving the medical department of, xxv. 442; sketches of practice in the, xxvii. 189, 284, 472; observations on the medical practice in the, xxviii. 89, 269; xxxii. 416. —*See* MEDICINA NAUTICA.

NAYLOR, Mr. case of tetanus successfully treated, x. 492.

NECROSIS, case of, of the tibia, xiv. 300; cases of, with remarks, xxix. 60; review of Mr. Whateley's treatise on, xxxiii. 510.

NEEDLE, aneurismal, observations on those of Mr. Watt and Mr. Henry Earle, xxv. 32, 238.

NEGRO, remarks on the skin of the, vii. 215; case of one who became white, viii. 97; case of gout in a, viii. 578.

NELSON, Admiral Lord, account of his death, xv. 72.

NEMNICH, M. account of his nosological lexicon in ten languages, x. 374.

NERVOUS SYSTEM, review of Mr. Purton's essay on the physiology of the, iv. 334; on the influence of galvanism on the, viii. 257; ix. 147; on the contraction of nerves under the influence of galvanism, 152, 235; observations on the nervous fluids, vii. 324; on disorders of the, xxii. 208; experiments relative to its influence on secretion, xxxi. 286; case of severe affection of, from a wound of the radial nerve, xxxi. 499; various observations on its functions, xxxiii. 23, 82, 198, 276, 351, 450; review of Gall and Spurzheim's work on the, 228, 485; observations on its influence on the action of the arteries, xxxiv. 54.—*See* BRAIN, SENSATION, LIFE, *and* VITALITY.

NETTLE-RASH, observations on the treatment of, x. 481.

NEW-CALEDONIA, observations on the earth-eaters of, viii. 191.

NEWTON, Mr. John Frank, review of his Return to Nature, xxvii. 312.

NEW-YORK, account of the epidemic there in the year 1805, xvii. 97.

NEYLE, Mr. observations on small-pox, and cow-pox, xvi. 138.

NEYRONIS, Mr. case of fatal pregnancy, with an unusual state of the uterus, ix. 470.

NICHOLAS, M. account of his experiments on the urine of patients affected with diabetes, ix. 12, xi. 338.

NICHOLSON, M. account of his galvanic experiments, iv. 113; vii. 478.

NICOLE, Dr. observations on the fever of Seringapatam, xxxiv. 244.

NICOTIANA.—*See* TOBACCO.

NISBETT, Dr. cases of cancer, and observations on the probability of curing that disease, iv. 296; v. 76; review of his treatise on diet, vi. 178.

NITRE, observations on its efficacy as a local application in gangrene, xi. 296; xii. 483; xiii. 324; xv. 504; its utility in the treatment of gun-shot wounds, xv. 97.

NITRIC ACID, observations on its utility in the treatment of venereal diseases, i. 100; Dr. Samuel Mitchill's remarks on its composition, &c. iv. 24; remarks on his opinion, vii. 217; case apparently shewing its efficacy in promoting the union of a fractured bone, xiv. 30; remarks on its utility in affections of the liver, xvii. 17; and in elephantiasis, 189; on its use in hepatitis, xviii. 301; its good effects in some cases of fever, xix. 106.

NITROUS GAS, remarks on its septic qualities and affinities, by Dr. Samuel Mitchill, ii. 180, 183, 380, 383; remarks on the opinions of Dr. Mitchill, vii. 317; ix. 304.

NOBLE, Mr. C. M. remarks on ophthalmy, iv. 358; on yellow fever, xv. 17.

———, Mr. Alexander, observations on cynanche parotidea occurring in a vessel at sea, xx. 180.

NOEF, M. on the medicinal qualities of the plantago angustifolia, vi. 276.

NOLI ME TANGERE, case of, cured by carbonate of iron, xv. 478.

NOMENCLATURE, *medical*, account of that of Dumas, iv. 73.

NOMENCLATURE, *chemical*, observations on the new, viii. 550.

NONAGENARIANA, his work termed the "Invalid," reviewed, xiii. 81.

NOOTH, Dr. on the treatment of dysentery, ii. 181.

———, Mr. observations on the treatment of schirrus and cancer, xii. 75.

NORMAN-CROSS, account of the hospital at, and the medical practice in, xii. 345.

NORRIS, Mr. William, account of the death of Professor Porson, xx. 389; case of severe contusion on the head, xxv. 165.

NORTHAMPTON, account of the infirmary at, viii. 192.

——— ———, Mr. account of the Harmattans, xxxiv. 409.

NORWAY, account of the diseases prevalent in, termed radesyge, vi. 13.

NOSOGNOMONIC EVIDENCE, remarks on, xix. 1.

NOSOLOGY, account of Dr. Hule's epitome of that of Sauvages, iv. 284; account of Dr. Hossack's, xxix. 280.

NOSWORTHY, Mr. description of a monstrous fœtus, xvii. 535.

NOTT, Dr. on the influenza of 1803, x. 80.

NOVASSILOZOFF, Dr. account of the successful treatment of a case of club-foot, iv. 496.

NUNN, Mr. account of a case of poisoning by oxy-muriate of mercury, ix. 247.

NUTRITION, observations on that of the fœtus, ix. 255; general observations on, and on nutrient substances, xxi. 509.

NYCTALOPIA, on that of tropical climates, xxvii. 165; observations on, xxxiii. 63.

NYMPHÆA, botanical description and medicinal qualities of, xvii. 68.

NYSTEN, account of his researches on physiological and chemical pathology, xxvi. 405.

### O.

O'BERNE, Mr. R. N. observations on the fevers of hot climates, x. 36.

OBESITY, remarks on, xxv. 494.

OBSTETRIC ART, reflections on the practice of it, xxiii. 13; observations on the treatment of uterine hæmorrhage during labour, xxiii. 461; observations on the management of twin cases, xxv. 286; remarks on the practice of, by men, xxxiv. 290, 450.—*See* Delivery.

ODIER, Professor, account of the death of De Sausserre, xvi. 470.

ODONTALGIA, observations on, iii. 403.

ODOURS, remarks on that exhaled from the body, considered as a symptom of health and of disease, i. 243; remarks on the different kinds of, exhaled by living animals, vi. 339, 341, 439; similar to that of musk exhaled by the human species, 342; observations on the particular effects of certain odours on the animal economy, xxiv. 223.

OEDEMA FUGAX, cases of, vi. 25, 168.

OENANTHE, botanical description and medicinal qualities of, xii. 360.

ŒSOPHAGUS, description of the operation of esophagotomy, vi. 97; of a new instrument for extracting substances from the, viii. 487; another instrument for that purpose, x. 479; case of stricture of the, ix. 552.—*See* Stricture.

OGDEN, Mr. his letter on the Cæsarean operation, ii. 476.

OHOPORDON, botanical description and medicinal qualities of, xix. 261.

OIL, chemical analysis of animal and vegetable, i. 76; utility of in arthritic affections, v. 490; observations on the medicinal use of, vi. 69; friction with, used as a preventive of infection of the plague, 247; method of purifying, viii. 383; used in the fevers of the West Indies, xi. 480; friction with, recommended in the fevers of hot climates, by Dr. Alcaraz, xii. 283; particular account of the mode of using it in friction, and of its effects, xiii. 17; friction with, used by Dr. White, in the British navy, in the year 1747, 18; employed by the Hungarians in the cure of fever, 118; remarks on the external use of, in hot climates, by Mr. Hunter, xvi. 80; the utility of olive oil as a purgative, xix. 51; observations on the mode of obtaining that of the ricinus, xxv. 498.

OKES, Mr. M. R. prescription for a styptic powder, xviii. 199.

———, Mr. T. V. review of his treatise on spina bifida, xxiv. 341.

OLDKNOW, Mr. observations on ligature of the vena saphena, xxi. 422; case of hydrophobia, xxii. 251.

O'LEARY, Dr. observations on yellow fever, xvi. 490.

OLIPHANT, Mr. observations on diseases of the breast mistaken for cancer, iv. 545; v. 188.

OLLENROTH, Mr. description of his instrument for removing extraneous bodies from the œsophagus, x. 479.

OMENTUM, case of suppuration of the, x. 29; case of wound of the, 356.

O'NEALE, Mr. case of gout treated by cold affusion, xiii. 328.

ONTYÆ, Dr. account of his enquiry into the influence of chemistry in the functions of animal bodies, vii. 261, 460.

OPHIOGLOSSUM, botanical description and medicinal qualities of, xix. 541.

OPHTHALMIA, remarks on the treatment of chronic, iv. 88; review of a treatise on, 358; observations on the endemic of Egypt, vi. 357; observations on, as it appeared in a British regiment, viii. 185; review of a treatise on the Egyptian, 555; review of another treatise on the Egyptian, ix. 575; on the use of burned sponge as an external application in, xii. 233; remarks on the nature of, 408; Baron Larrey's treatment of the Egyptian in the French army, 520; remarks on Mr. Larrey's treatment, xiii. 150; observations on the use of cold affusion in the treatment of, xiv. 239; on the treatment of the Egyptian, xvi. 283, 575; general observations on, xvii. 79, 193; on the Egyptian, 317; various observations on, 379, 385; on the puriform of children, 471; on the Egyptian, 479; xviii. 12, 474; queries respecting it, 493; observations on an epidemic at Edinburgh, 578; xix. 473, 571; utility of the actual cautery in some cases of, xix. 430; diagnosis between infectious and the common, 573; case of purulent in an infant, whose mother was affected with gonorrhœa, xx. 57, 237; on that prevalent at Edinburgh, 90; utility of stimulating applications in some cases of, 190; observations on painful sensibility to light, 472; remarks on the Egyptian, xxi. 164; observations on, xxii. 42, 116, 167, 170; on the Egyptian, 260; on the purulent of infants, 363; on the most frequent origin of, 457; various observations on, 522; remarks on the purulent, xxiii. 77, 154; cases of, with increased secretion of the aqueous humour, 125; on the origin of the Egyptian, xxiv. 130, 317; on the varieties of, prevalent in the army, xxv. 357; utility of the metallic oxides in the cure of, 497; remarks on the Egyptian, 522; review of Mr. Saunders's treatise on, xxvii. 230; observations on the Egyptian, xxix. 302, 452; on the efficacy of tartar emetic in the treatment of the Egyptian, xxx. 29, 46, 302; objection to that mode of treatment, xxxi. 17; vindication of it, 189, 355; cases of, cured by tartar emetic, 272; on the treatment of the Egyptian, 326; on the utility of evacuating the aqueous humour in some forms of, 504; on the treatment of the Egyptian, xxxii. 486.

OPIUM, on the best mode of preparing the extract of, i. 398; xxii. 351; on the utility of friction with, 440; efficacy of frictions with, in the cure of some ulcers, ii. 108; observations on its effects on the animal economy, 233, 467; on the use of frictions with, iii. 102; the utility of it when combined with oxy-muriate of mercury, in syphilitic exostoses, 168; utility of it with æther, in spasms of the stomach, iv. 444; combined with nitrous acid, beneficial in the treatment of dysentery, iii. 413; the deleterious effects relieved by the excitement of pain, illustrated by a case, 574; on the preparation of the tincture of, 582; case of salivation produced by, 586; case of severe effects of a large dose, iv. 512; applied externally with success in the treatment of epilepsy, 570; case of the deleterious effects of, v. 491; efficacy of, in the cure of tetanus, vi. 280; its sedative effects, when applied externally, 478; recommended in hydrophobia and tetanus, 479; and cases of its efficacy, in various affections, when applied externally, 480; on the mode of its agency on the animal economy, with experiments, vii. 124; objections to the opinion that it is immediately stimulant, drawn from experiments on rabbits, frogs, &c. 342; and on man, 357; its sedative quality considered, 497; observations respecting its utility in fever, viii. 50; its exhilirating qualities, 325; remarks on Brown's opinions on its mode of agency, 333; experiments with it, on frogs, 340; remarks on Mr. Crump's theory of its agency, 389; objections to the opinion that it is an immediate stimulant, ix. 335; observations on its efficacy, when applied externally in hydrophobia and tetanus, xi. 538; its efficacy in dysentery, when combined with sulphate of zinc, ix. 55; chemical analysis of, 398; x. 42; its efficacy in the treatment of puerperal fever, x. 16; observations on its effects on the system, 435, 501; remarks on Mr. Ward's doctrine of its mode of agency, 532; its use in the treatment of intermittent fever, xii. 4; observations on its cultivation, and on its agency on the system, 39; medical and chemical qualities of, 42; efficacious in the treatment of colic, xiii. 19; its efficacy in remedying the effects of the bite of the *coluberberus* LIN. xiv. 524; its utility in removing some cases of intestinal obstruction, xv. 337; its efficacy in the treatment of rheumatism, xvi. 496; case of fatal effects of, xvii. 422; its utility in preventing the ill effects of mercury on the bowels, xviii. 192; effects of a large dose of, xix. 497; xxii. 78; the utility of friction with, in febrile and spasmodic diseases, xxiii. 351; recommended in the treatment of syphilis, xxiv. 45; its great efficacy in the treatment of gonorrhœa, when combined with calomel, xxv. 445; its utility in rheumatism, xxvii. 284; effects of a large dose of, xxviii. 80; observations respecting its utility in tetanus, xxix. 100; its utility when combined with ipecacuanha, in dysentery, xxx. 75; observations on its efficacy in the alleviation of parturition, 303; its efficacy in some cases of constipation of the bowels, 441; cases of its efficacy in diabetes, xxxi. 152; fatal effects of a large dose of, 198; case of recovery from the effects of a large dose of, 468; on its use in uterine hæmorrhage, xxxii. 153; on the bad effects of blood-letting in cases of poisoning by, 271; its utility in a case of gangrene of the feet, 430; cases of recovery from the effects of a large dose of, xxxiii. 201, 350.

OPOSSUM, on its mode of delivery of its young, xxvi. 422.

OPTICS, observations on, by Dr. Walker, xvii. 522; observations on, connected with the presence of cataract in the eye, by Mr. Carmichael, xix. 313; various observations relative to, xxii. 307; observations on some phenomena of vision, xiv. 11, 117, 153; description of an organ by which the eyes of birds are accommodated to distant objects, xxix. 481; observations on perfect vision at different distances, xxxi. 247; xxvii. 257; xxxii. 243; observations on, relative to insects, 314, 400, 500. —*See* VISION.

ORCHIS SATYRION, botanical description and medicinal qualities of, xi. 441.

ORFILA, Dr. notice of his toxicology, xxxiii. 400, 471.

ORIGANUM, botanical description and medicinal qualities of, xvii. 565.

ORNITHROGALLUM, botanical description and medicinal qualities of, xiv. 63.

ORNITHORYNCHUS PARADOXUS, observations relative to the natural history of the, vi. 73.

OROBUS, botanical description and medicinal qualities of, xix. 77.

OSBORNE, Dr. remarks on his opinions respecting embryulcia, iv. 168; remarks on his opinions respecting the physical necessity of pain in parturition, xviii. 450, 545.

OSGOOD, Dr. case of extra-uterine gestation, xxiii. 462.

OS, *intermaxillare*, on the different forms of, in various animals, v. 566; *orbiculare*, anatomical queries relative to it, 349.—*See* BONES.

OSSIFICATION, two curious instances of preternatural, iii. 587; physiological observations on, xiii. 385.

OSMUNDA, botanical description and medicinal qualities of, xix. 542.

OTAHEITE, observations on the diseases of, xvi. 174.

OTTO, Dr. observations relative to the use of arsenic, xiv. 113; history of a family disposition to hæmorrhage, xx. 69.

OVARIUM, case of dropsy of the, ii. 20; case of remarkable enlargement of, viii. 444; case in which teeth and hair were present in the, xxiii. 237; cases of dropsy of the, xxx. 162; xxxiv. 103.

OVUM, observations on the formation of the human, xv. 271.—*See* EGG.

OXALIS, botanical description and medicinal qualities of, xv. 266.

OXFORD, observations on the mode of graduating in medicine at, vii. 48.

OWEN, Mr. his account of an ancient document relative to syphilis, ii. 272, 416.

OXLEY, Mr. account of his essay on the properties and medicinal qualities of the pellitory of Spain, (*anthemis pyrethrum,*) v. 329.

OXYDES, observations on that formed on the galvanic pile, xi. 63.

OXYGEN, observations on its utility in the treatment of syphilis and of psola, i. 144; on its medicinal qualities, ii. 38; on the claim of Girtanner to the application of it in the cure of syphilis, iv. 469; an enquiry into its efficacy in the cure of syphilis, x. 90; its utility in convulsive affections, xv. 592; chemical observations relative to, xvii. 197; observations on its efficacy in the cure of intermittent fever, 447; observations on its medicinal qualities, xxvii. 14.

OXYMURIATIC ACID-GAS, the doctrine of its elementary nature, xxiv. 345; xxv. 83; on the combination of it with oxygen gas, xxvi. 298; its anti-contagious properties, xxvii. 408.

P.

PACCHIONI, Sig. on the composition of the muriatic acid, xiv. 272.

PACK, Mr. remarks on the expediency of early delivery of the placenta, iii. 221, 517.

PADUA, account of the medical school of, xxxi. 81.

PAGET, Mr. on the use of belladonna in operating for the cataract, vi. 352; case in which a calculus weighing twenty-seven ounces was taken from the urinary bladder, vi. 391; on the use of belladonna for dilating the pupil, xxx. 511.

PAIN, physiological reflections on, xxxi. 97.

PALLADIUM, description of it, x. 96.

PALMER, Mr. case of hydrocephalus, xiv. 237.

PANCREAS, observations on its functions, xvi. 193, 289; cases of indurated enlargement of the, xxv. 185.

PAPAVER, botanical description and medicinal qualities of the, xvi. 559.

PAPAYA, on the anthelminthic qualities of its juice, ix. 79.

PAPULA, distinction between it and phlyctis or vesicula, xi. 232.

PARACENTESIS, *of the chest,* instances of, performed with success in a case of abscess, xiii. 22; xxxii. 130.

————, *of the abdomen,* advantages attending the performing it at the navel, xiv. 410; xvii. 29; objections to it, 352; case of opening into the abdominal cavity performed through the vagina, xxvii. 434.

PARALYSIS, of the exterior muscles of the hand and fingers, accompanied by a separation of the bones of the metacarpus, case of, iii. 253; rheumatic, and paraplegica, successfully treated by the pellitory of Spain, v. 331, 332; fatal case of, from rolling in the hands a composition containing white arsenic, v. 542; utility of the toxicodendron in the treatment of, vi. 272; of the pupil, utility of hyosciamus in, 275; cases of, successfully treated by galvanism, vii. 245; traumatica, case of, viii. 494; efficacy of the air-pump vapour-bath in the treatment of, viii. 60; of the anus and bladder, galvanism employed for, viii. 256; ix. 135; galvanism used for, in cases affecting the extremities, ix. 137, 322; a case of, cured by the air-pump vapour-bath, ix. 175; two cases cured by galvanism, x. 60; cases of, consequent on acute rheumatism, xv. 45; cases in which volatile alkali was very beneficial, xvii. 428; general observations on, 483; cases of, from want of accustomed stimulant diet, xviii. 154; observations on, 279; cases of, from lightning, xix. 119; remarkable case of, xxii. 227; on the efficacy of blood-letting and purging in, xxiv. 375; instance of its disappearing, after an attack of gout, xxvi. 153; cases of, cured by titillation, xxviii. 224; cases of, shewing its connexion with disorder of the stomach, xxviii. 453; further observations respecting its connexion with disorder of the gastric system, xxix. 462.

PARAPHYMOSIS, cases of, with observations, xvi. 140.

PARÉ, Ambrose, his account of tympanitis, vii. 230; his claims to the introduction of the use of the ligature, xiii. 391, xiv. 40, 103.

PARIETARIA, botanical description and medicinal qualities of, xii. 30.

PARIS, Dr. Ayrton, reflections on animal heat, xxi. 67; observations on the utility of muriatic acid in febrile complaints, xxiii. 51; on the physiology of the eye, xxxiv. 164; review of his pharmacology, xxviii. 69.

PARIS, (the plant,) botanical description and medical qualities of, xv. 68.

PARK, Mr. H. observations on vaccination, xxv. 304.

PARKER, Mr. observations on a mode of extracting the blood from leeches, xiv. 180.

PARKINSON, Dr. W. cases of ischuria, xx. 115; Mr James, review of his Medical Admonitions, i. 310, 491; his letters to facilitate the study of medicine, iv. 459; account of his Chemical Pocket-book, v. 287; his observations on gout, xv. 175; observations on the formation of marble, xxi. 439; observations on the Bill for regulating madhouses, xxvi. 148.

PARKINSON, Dr. T. observations on the efficacy of alcohol in the treatment of burns, xxiv. 98.

PARMENTIER, M. his new method of preparing the extract of opium, xxii. 351.

PAROLETTI, Sig. observations on morbid exhalations, xxiii. 350.

PARRY, Dr. Caleb Hillier, observations on angina pectoris, iii. 389, 476; observations on the utility of blood-letting in purpura, xxi. 146; case of nervous affection cured by compression of the carotids, xxiv. 456; review of his treatise on tetanus and rabies contagiosa, xxxiii. 315; review of his treatise on the arterial pulse, xxxiv. 80, 163.

PARSNIP, account of the production of sugar from, iv. 349.

PARTURITION, observations on the diseases consequent on, ix, 281; observations respecting the management of, in animals, xxix. 66; synopsis of various kinds of, xxix. 423; cases of, induced prematurely by puncturing the membranes in the eighth month, in a patient with a deformed pelvis, v. 312; xxxii. 100; observations on the occurrence of sudden death soon after, 135; on the efficacy of the ergot (*secale cornutum*) in some cases of difficult, 90, 192.—*See* Delivery.

PASCALIS, Dr. Felix, case of abscess of the liver, xvii. 500; observations on scarlatina anginosa, xx. 121.

PASCHMANN, description of his anemometer, xii. 283.

PASAMOT, M. observations made during a voyage in the Pyrenees, iv. 187.

PASTINACA, botanical description and medicinal qualities of, xii. 371.

PATELLA, account of a bandage for a dislocated, iv. 285.

PATERSON, Dr. of Montrose, observations on vaccination, vi. 36.

PATHOLOGY, general observations relative to, xxx. 25; on the division of diseases into general and local, 180; review of Dr. Farre's Researches, xxxii. 515; remarks on general and local diseases, xxxiii. 458; cases and dissections relative to, xxxi. 274; review of Dr. Monro's treatise on, xxviii. 509.—*See the particular Organs of the* Body.

PATRICK, Mr. observations on nervous diseases, xxii. 208.

PATTERSON, Dr. of Londonderry; observations on the medicinal qualities of digitalis, v. 441; reflections on what are considered to be new discoveries, vi. 407; history of an uncommon tumour, 309; cases of asphyxia azotica, vii. 511; observations on the yellow fever, ix. 105, 302; case of angina pectoris, x. 63; cases of disease of the brain, xii. 109, 196; on vaccination, xiv. 437; on epidemic diseases, 574; observations on chorea, xv. 119, xviii. 213.

PAUL, M. account of his artificial mineral waters, viii. 86; his letter on artificial Cheltenham water, x. 574.

PAULET, Mr. review of his treatise on champignions, xxiii. 68.

PEACH-TREE, the medicinal qualities of its leaves, iii. 563.
PEAKE, Mr. history of the appearances on dissection in two rabid dogs, xxv. 494.
PEAAL, Mr. Tuam, observations on autumnal fevers, vii. 320; on the influenza of 1803, x. 232; on the temperament and diseases of the people of the highlands of Scotland, xiii. 28.
PEARS, Dr. review of his work on phthisis, xxxii. 227.
PEARSE, Mr. C. of Newington Butts, history of a case of lepra grecorum, vi. 124; observations on vaccine inoculation, vii. 249; on some supposed failures of the prophylactic power of vaccination, viii. 189, 547.
———, Mr. of Honiton, observations on the influenza of 1803, x. 203.
PEARSON, Mr. H. observations on the prevalent diseases of hot climates, ii. 158, 200; on the influenza that prevailed at Whampsa in China, in 1800, xiii. 488.
———, Dr. George, on the origin and progress of vaccine inoculation, i. 5, 113; ii. 213; on the eruptions resembling the small-pox that sometimes appear after cow-pox, iii. 97; on the evidence relative to vaccination, iii. 399, 411; on inoculated cow-pox, v. 86; his evidence before a committee of the House of Commons on the subject of vaccine inoculation, viii. 143; observations relative to that evidence, 156, ix. 48, 95; on the influenza of 1803, ix. 377; observations relative to matter expectorated from the lungs, xxiii. 214; observations and experiments on pus, xxv. 326; on the colouring matter secreted by the lungs, xxxi. 128.
———, Mr. John, observations on the various articles of the materia medica used in the cure of syphilis, iii. 564; observations on the use of cold affusion in typhus, viii. 356; his Croonian lecture in the year 1807, xvii. 93; review of his observations on the effects of various articles of the materia medica in the cure of syphilis, xvii. 375.
———, Dr. Richard, observations on the treatment of bilious fevers, iii. 94; review of his work on the influenza of 1803, ix. 377; on the prevention of contagious diseases, xiii. 272; on the hooping-cough, xxii. 71.
PEART, Dr. E. remarks on his treatise on scarlet-fever, enteritis, strangulated hernia, erysipelas, erythema, and measles, viii. 471.
PECK, Mr. observations on the extraction of the placenta, iii. 529; on the use of caustic alkali in the treatment of ulcers, vi. 23; reflections on quackery, vi. 129.
PECKHAM, Mr. observations on the management of leeches, xxiii.
PEDICULARIS, botanical description and medicinal qualities of, xviii. 170.
PEGGE, Sir Christopher, observations relative to the origin of the vaccine disease, iv. 381.
PELLETAN, Mr. observations on some diseases of the heart, xxxi. 217.
PELLITORY OF SPAIN, its medicinal qualities, v. 329.
PELVIS, description of the female, iv. 168; on the production of premature delivery in deformity of the, v. 312, xxxii. 100.—*See* PUBIS.
PEMBERTON, Dr. review of his treatise on diseases of the abdominal viscera, xv. 481, 573; his Harveyan oration in 1786, xvii.
PEMPHIGUS, observations on it, iii. 552; cases of, xviii. 43; observations on, as it affects children, xix. 344; description of, xx. 376; cases of, xxxi. 265; historical researches respecting it, xxxii. 281; instances of it appearing epidemically, 165; case of, xxxiv. 171.
PENEL, Dr. remarks on some singular phenomena observed in a case of fracture of the thigh, xiv. 29.
PENIS, case of amputation of, for cancer, xix. 270; observations on an acute eruptive disease of, xxiii. 441; case of inflammation and suppuration of the corpus spongiosum of the, xxiv. 267.
PENKIVIL, Mr. case of rheumatic inflammation of the heart, xxxii. 22.
PEPYS, Sir Lucas, observations on the medical officers of the army, xxiii. 438.
——— Mr. account of his galvanic experiments, ix. 390.
PERCIVAL, Dr. Thomas, observations on the application of opium by friction, i. 442; review of his Medical Ethics, xi. 181; biography of, xix. 271.
——— Dr. Edward, history of a fatal case of cynanche laryngea, xxxii. 147; on the use of spirit-of-turpentine in epilepsy, xxx. 509.
PERCY, Baron, observations on a case of universal anchylosis, viii. 284, 393; observations on adhesion by the first intention, xxxiii. 424.
PERICARDIUM, observations on inflammation of the, xxi. 265; case of inflammation of the, xxxi. 274.
PERIPNEUMONIA TYPHOIDES, observations on, xxxi. 471.—*See* Lungs.

PERITONEUM, cases of inflammation of the, xxx. 333, 408; xxxiv. 11, 443.

PERIOSTEUM, observations on inflammation of the, xvi. 513.

PERON, Mr. description of the medusa, xxii. 526.

PERPERES, observations on its use in dyspepsia, xx. 193.

PERRY, Mr. case of delirium tremens, xxxi. 40.

PERSPIRATION, cutaneous, physiological inquiry respecting the, i. 287; instances of its affecting one-half of the body, xxxi. 392.

PESSARY, the use of sponge, recommended, xiii. 130; observations on the sort generally employed, 131; instances of the superior utility of the sponge, xiv. 98; xv. 21, 235.

PESTILENTIAL DISEASES, observations on some in the United States of America that were preceded by the glandular disease of horses, i. 79; observations respecting the origin of, xxi. 241, 281; amongst horned-cattle in France, history of, xxxiii. 161.—*See* Contagion, Contagious Diseases, *and* Plague.

PETECHIÆ, without fever, cases of, i. 420; three cases of, x. 444; case of, xxx. 339.—*See* Purpura.

PETIT, M. observations on entero-mesenteric fever, xxxiv. 336.

PETRIFACTION, observations on the process of, xviii. 304.

PETROFF, case of effect of lightning on a man, xvii. 50.

PETTIVER, Mr. description of the storax-tree, i. 164.

PEUCEDANUM, botanical description and medicinal qualities of, xx. 363.

PEW, Dr. Richard, observations on the influenza of 1803, x. 523; observations on an eruptive disease, somewhat resembling small-pox, occurring after vaccination, xxi. 250.

PFAFF, Professor, of Kiel, notice of his northern archives of medicine and natural history, iv. 73.

PFEIFFER, Dr. history of a case shewing the efficacy of salivation in the treatment of phthisis, viii. 308.

PHACA, description of a new species of, viii. 282.

PHARMACOLITH, a new fossil, description of, vi. 570.

PHARMACOLOGY, universal system of, by Professor Vattelm, iii. 576; review of that of Dr. Paris, xxviii. 69.

PHARMACOPŒIA, analysis of the Prussian, iii. 93; observations on the London, xi. 299, 534; historical researches respecting works of this kind, xii 55, 103; notice of Dr. Swediaur's, 565; remarks on them in general, xiii. 217; remarks on the Dublin, xviii. 540; account of the new London of 1809, xx. 137; remarks on the new London, xxii. 10, 269; xxv. 431, xxviii. 221; review of the translations of the new London, xxxiv. 488.

PHARMACY, review of a treatise on, xi. 562; miscellaneous observations respecting, xxiv. 224; review of a treatise on, xxvi. 321.

PHELLANDRUM, *aquaticum*, recommended in the treatment of consumption, vi. 95.

PHILIPS, Mr. of Painswick, observations on vaccination, v. 547.

——— case of a fœtus in the body of a child, xxxiv. 80.

PHILIP, Dr. A. P. Wilson, observations on digestion, x. 318; review of his treatise on febrile diseases, xiii. 554; xviii. 462; remarks on the diagnosis between fever and phrenitis, xix. 179; on the nature of inflammation, and its connexion with fever, xx. 174; his account of some experiments relating to the opinions of Bichât, xxii. 253.

PHILLIPS, Mr. R. observations on the London Pharmacopœia, xxviii. 226.

PHILO-MEDICUS, remarks on degrees in medicine, vii. 12; strictures on his opinions, 260; reply to, 271; further remarks, 366.

PHISELDECK, Dr. L. Von Schmidt, his mode of preparing sublimate of mercury xi. 340.

PHLEGMASIA DOLENS, Dr. Ferriar's remarks on Dr. Hull's essay on, v. 4; remarks on Mr. White's treatise on, vi. 51, 135, 220; case of, after abortion, xv. 5; observations on, xxiii. 300; cases of, and remarks on the different theories of the disease, xxxii. 187, 524.

PHLEGMON, remarks on the diagnosis of, xx. 371.—*See* Inflammation.

PHLYCTIS, diagnosis of, xi. 232.

PHONICS, theory of, xix. 384.

PHOSPHAT, *ammoniaca-magnesian*, its physical character and chemical properties, ix. 494.

PHOSPHORESCENCE, observations respecting, xviii. 289; xix. 479; xxiii. 23; observations on that of animals, xxv. 409, 512.

PHOSPHORUS, its combustion in azotic gas, disproved by Brugmans, Fourcroy, and Vauquelin, i. 85; its deleterious effects on animals when suspended in a fluid, ibid.; chemical observations on, 299, 300; proposed by Professor Grew, as a eudiometer, ii. 389; preparation of, for medicinal use, iii. 270; iv. 161; observations on its medicinal use, v. 396; a solution of it in oils, used by friction, useful in rheumatic, arthritic, and syphilitic pains, vi. 378; influence of light on, when dissolved in gases, vi. 374; experiments relative to its combination with gases, vii. 374; observations on its medical qualities, ix. 299; the mode of purifying it, xi. 178; preparation and properties of the white oxide of, 179; exhibited for the cure of chorea, xiii. 402; mode of preparing bottles of, for obtaining light, xxiv. 86; observations on its combination with sulphur, xxx. 83.

PHOSPHURET OF LIME, its chemical characters, ix. 290.

PHRENITIS, case of idiopathic, xiv. 391; case of, from external injury, xxxi. 359.—*See* BRAIN.

PHRENOLOGY, (or CRANIOLOGY,) account of the system of Gall, and Spurzheim, xiv. 327; xxxiii. 228, 485.—*See* BRAIN, *and* CRANIOLOGY.

PHTHIAN, M. observations on medical electricity, xxvii. 451.

PHTHISIS.—*See* CONSUMPTION, *and* LUNGS.

PHYMOSIS, observations on the treatment of, iii. 162.

Φυσεως Ακολυθος, his reasons for eating animal food, xxvii. 47.

PHYSICIAN, observations relative to the character of a, v. 475; remarks on the conduct of a, xix. 204, 350; account of Mr. John Bell; letters on the character of a, xxiv. 343; observations on the conduct of a, xxv. 313; observations on the privileges of the licentiates of the London College, xxviii. 49.—*See* MEDICINE.

PHYSICK, Dr. observations on the utility of blisters in checking mortification, xviii. 3.

PHYSIOGNOMY, review of a treatise on, relating to man, xxviii. 488; remarks on that of diseases, xxvii. 38.

PHYSIOLOGY, remarks on the system of Professor Schmidt of Jena, iii. 181, 280; various observations and reflections on, v. 476, 563; vi. 376, 449, vii. 472; xxii. 258, 333; xxxii. 57; review of an elementary treatise on, x. 567; account of the researches of Bichât; observations on the transformation of the organs of the body, xvi. 301, 451, 517; xviii. 36; various observations and reflections, xviii. 75, 84, 178, 215, 217, 224; on the torpidity of animals, xix. 283; the theory of phonics, 384; observations on the effects of climate on the form and colour of the human species, xx. 245, xxi. 85; a new arrangement of the vital functions, xx. 44; first principles of, 99; reflections on abstinence, 527; various observations on, xxi. 102; xxiii. 451; xxiv. 8; remarks on the axiom of the incompatibility of the simultaneous existence of two diseased actions, 110; various reflections on, xxvi. 58; xxvii. 18; review of the treatise of Mr. Saumarez, xxviii. 330, 414; general observations on, xxix. 141, 497; various reflections on, xxx. 429; review of the work of Legallois, xxxi. 417; review of Blumenbach's Institutes of, xxxiii. 279.—*See the different Organs and Functions of the* BODY.

PHYTOLACCA, its medicinal qualities, xvii. 294.

PHYTOLOGY, observations on the irritability of plants, xx. 569; various reflections on, xvii. 196; xxiv. 433; xxv. 56; xxvi. 8, 72, 291; xxvii. 406, 432; xxx. 251, 429.—*See* PLANTS.

PICTET, Mr. notice of his experiments on light and heat, viii. 572.

PIGOT, Dr. observations on purging in the treatment of typhus fever, xxvii. 459; xxxviii. 289.

PILE, *voltaic*, on its application to medicinal purposes, vii. 529; remarks on its poles, viii. 252; some new facts respecting it, 478.—*See* GALVANISM.

PIMPINELLA, botanical description and medical qualities of, xii. 556.

PINCKARD, Dr. review of his Notes on the West Indies, xv. 561; xvi. 263; observations on the effects of climate on the form and colour of the human species, xx. 245; history of a case of hydrophobia, xxi. 53; case of hydrophobia, xxviii. 449; case of hydrophobia, xxxiv. 177.

PINUS, botanical description and medicinal qualities of the, xviii. 460.

PISCIS, botanical description and medical qualities of, xix. 166.

PITCAIRN, Dr. biography of, xxi. 459.

PITNA, Dr. review of his treatise on the influence of climate on man, xx. 85.

PITT, Mr. case of fractured skull, xiii. 445; an account of the analysis of a meteoric stone, xxi. 85.

PLACENTA, case of unusual adhesion of the, iii. 6; observations on adhesion of the, 333; remarks on the expediency of early delivery of the, 221, 517; on the extraction of the, 421, 459; on adhesion of the, 447; remarkable case of expulsion of the, 482; remarks on the expulsion and extraction of the, 536; on the danger attending forcible delivery of the, iv. 3; on the expediency of early delivery of the, iv. 156; remarks on the delivery of the, 320; x. 433; xi. 63; observations respecting the functions of the, xvii. 164; case in which a large mass resembling it, but without a funis, was expelled from the uterus, xxxiii. 233.

PLAGUE, some remarks on its nature, vi. 255, 365; on the inoculation of the, v. 243; observations on the memorable epochs of its appearance, and its treatment, iv. 364; cases of, as it appeared in a British ship of war on the coast of Syria, in 1799, v. 538; its contagious nature denied, v. 5, 292; on the prophylactic and curative effects of oil used in friction, vi. 247; on the efficacy of mercury and nitric acid in the treatment of the, xii. 79; remarks on its importation, 91; opinion that it arises in Egypt from the exhalation after the overflowing of the Nile, ibid; observations on its origin, symptoms, and progress, xv. 113; attributed to bad state of the air, 114; the influence of the simoons on it, 114; originates in the environs of Damietta, ibid; it originates in Greece about the lakes of Albania and the Morea, 115; its symptoms particularly severe in childbed women, 116; prophylactic measures, 118; not caused by contagion, xvii. 506; on the time when it chiefly prevails, 506; observations on its origin and propagation, xviii. 115, 470; observations on its treatment, xxi. 106; account of that which depopulated Barbary in 1799 and 1800, xxii. 193; account of, amongst the horned cattle in Grenada in 1783, xxiii. 166, 430; objections to the opinion of its being contagious, xxvii. 272; account of that of Malta, xxxiii. 477.—*See* Contagion, Contagious Disease, Pestilence, *and* Miasma.

PLANCHE, Mr. account of the angustura bark, xxii. 144.

PLANTAGO, botanical description and medical qualities of, xii. 28.

PLANTS, description of Hungarian, i. 368; phytological experiments, iii. 175; enquiries respecting the nature of the irritability of, ix. 291; on the distilled waters of inodorous, x. 95; catalogue of British, xi. 326, 524; xii. 28, 124; xv. 261; xvi. 73, 165, 261, 557; xvii. 68, 371, 464, 558; xviii. 169, 270, 363; xix. 71, 162, 257, 353, 435, 538; on the motion of the sap in, xiv. 207; *poisonous*, observations on, 425; on the culture of, xviii. 386; observations on the Chinese mode of propagating fruit-trees, xxi. 84; phytological observations, xxii. 107; on the general destruction of, in 1809, xxiv. 433; various observations on, xxiv. 438, 528; xxv. 82, 187, 248, 370, 452, 549.—*See* Botany.

PLATINA, on the crystallization of, iv. 373; account of some experiments on, xi. 288; notice of some found at St. Domingo, xxv. 509; observations on its utility in mechanics, xxi. 262.

PLATT, Mr. review of his enquiry into the efficacy of oxygen in the cure of syphilis, x. 90; xiii. 78; on the existence of ulceration of the urethra in gonorrhœa, 548.

PLAYFAIR, Mr. observations on dysentery, xxx. 75.

PLEURA, observations on inflammation of the, xviii. 392; xxxi. 277.

PLICA POLONICA, observations on its origin and nature, xvi. 235; xxiii. 204, 388; xxii. 500; review of La Fontaine's work on, xxiv. 329.

PLOUQUET, Prof. his reflections on the state of the trachea in drowned persons, vi. 93; his opinions on the local origin of fever, xix. 169.

PNEUMATIC CHEMISTRY, observations on its application to the cure of diseases, i. 143.

————, *doctrine*, remarks on the, xiii. 80.

PNEUMONIA TYPHODES, review of an essay on, iii. 279.—*See* Lungs.

POISONS, observations on the prophylactic qualities of the balm of Gilead, i. 34; observations on the introduction of them into the system, 449; on the fever excited by venereal, iii. 198; on the deleterious effects of opium, 574; remarkable effects from an unknown, iv. 516; deleterious effects from opium, v. 491; morbid, observations on, v. 498; explanation of Hunter's doctrine of the venereal, viii. 421; deleterious effects of opium, ix. 247; morbid, observations on, ix. 309; on the varieties resembling syphilis, xiii. 79; case of death from the external use of tobacco, xiv. 305; on the use of alkali in remedying the effects of animal, 478; case of death from the sting of a wasp, 479; case of death from the effects of opium, xvii. 569; morbid, observations on, xviii. 82; account of the deleterious agency of champignons, xx. 459, 566, 567; on those of fish, 461; observations on the presence of the mineral in the stomach after death, xxi. 148; experiments with the *upas tieuté*, xxii. 96; various observations on vegetables, xxv. 361, 398; researches respecting the mode in which the vegetable poisons cause death, xxvi. 129; various observations on, xxvii. 3, 429; fatal effects of bitter almonds in a child, xi. 92; deleterious action of cerussa acetata, ix. 173; case of death from champignons, iii. 41; xv. 247; xx. 352, 457; deleterious action of cantharides, xviii. 55; the poisonous qualities of prussic acid, xi. 193; deleterious agency of agaric, iii. 41; case of death of a child from arsenic, iv. 293; other cases of poisoning by arsenic, xvi. 95; xxii. 427; xxiii. 384; xxiv. 59; xxviii. 63, 345, 349; xxix. 44; xxxiv. 441; remarks on poisoning from arsenic, xi. 192; xxiii. 238; xxvi. 32; xxviii. 117, 123; xxix. 133, 136; xxxi. 8, 178; xxxiv. 443; remarks on the poisonous effects of mercury, xxx. 342; account of 180 persons being poisoned by belladonna, xxxii. 390; on the detection of mineral poisons in the stomach, xxi. 148, 340; account of the evidence on the trial of Mr. Angus for suspicion of poisoning Miss Burns, xxi. 340, 343, 345, 427, 430; xxiv. 38; xxxii. 327; remarks on the power of alkali in counteracting the deleterious effects of corrosive sublimate, iii. 535; xxviii. 223; case of death from the external application of corrosive sublimate, xxviii. 223; remarks on the poisonous qualities of stramonium, xxv. 500; xxvi. 22; on the poisoned honey of Heraclea Pontis, by Dioscorides, xxxii. 393; case of death from the evolution of septic gas, xvii. 225; cases of several persons poisoned in Scotland by eating some species of fungus, xxxii. 364; observations on the effects of a large dose of arsenic, xxviii. 65; of opium, 80; observations respecting their action on the system, 115; cases of death from oxymuriate of mercury, 223; effects of the bite of the *choppa poora*, (an Indian black snake) xxix. 122; effects of various minerals, 133; observations on those of serpents, 122; from arsenic, 64, 345; from opium, xxxi. 198; effects of a large dose of opium, 468; xxxii. 271; observations respecting the effects of colchicum, 216; of upas antiar, 343; of belladonna, 390; of mushrooms, 364; of opium, xxxiii. 201, 350.

POLE, Dr. meteorological observations made at Bristol from 1802 to 1809, xxi. 435; thermometrical and barometrical observations, xxxiii. 255.

POLE, Mr. history of a case of monstrosity, iii. 397, 497; observations on vaccine inoculation, v. 340.

POLIDORI, Dr. observations on the treatment of typhous fever, i. 104.

POLLOCK, Mr. observations respecting the submersion of swallows, xix. 352; case of tænia cured by a decoction of pomegranate, xxxii. 395.

POLYDIPSIA, case of, iii. 509; observations on the cure of, iv. 304; case of, and remarks on, 403.

POLYGALA, botanical description and medical qualities of, iv. 72.

POLIGONIUM, botanical description and medical qualities of, xv. 66.

POLYPODIUM, remarks on its vermifuge qualities, xx. 125; botanical description and medical qualities of, 157.

POLYPUS, of the uterus, case of, iii. 1; of the lungs, cases of, viii. 201, 360; of the antrum of Highmore, case of, from a wound, xii. 5; case of nasal, xiv. 179; in the nose, case of, xvi. 7; of the bronchial membrane, case of, xx. 471; of the heart, observations on, xxii. 511.—*See* TUMORS.

POMMADE, *oxygenated*, a mode of preparing it, iii. 580.

POMEGRANATE, its efficacy in the cure of tænia, xxxii. 395.

PONTIER, M. case of epilepsy, x. 51.

POPE, Dr. his evidence before a committee of the House of Commons, respecting vaccination, viii. 20.

———, Mr. case of hernia, xiv. 454; remarks on the treatment of gout, xviii. 52.

POPULUS, botanical description and medical qualities of, xiv. 542.

PORTLAND POWDER, remarks on the use of it, v. 417; xiii. 146.

PORRIGO, observations on a species affecting the scalp, xxi. 132; review of Dr. Willan's treatise on, xxxii. 128.

PORTAL, M. observations on the efficacy of frictions with spirit of hartshorn in the cure of epilepsy, iv. 380; some reflections on the action of the spinal marrow, vi. 370; his opinions respecting epilepsy, 377; on the nature and treatment of apoplexy, xxx. 193, 371; review of his treatise on fever, xxxi. 425.

POTASH.—*See* ALKALI, *and* KALI.

POTASSIUM, account of the production of, xxii. 12; Mr. Tenant's mode of obtaining it, xxxiv. 26.

POTENTILLA, botanical description and medical qualities of, xvi. 264.

POTERIUM, botanical description and medical qualities of, xvii. 70.

POTTER, Dr. of Baltimore, remarks on American epidemics, xi. 309.

POTTS, Mr. on the mode of supplying the loss of limbs, v. 277; vi. 445.

POUQUEVILLE, M. observations respecting the plague, xv. 113.

POWELL, Dr. history of a case of hydrophobia, xx. 201; on the effects of the nitrate of silver, xxxi. 148; review of his translation of the London Pharmacopœia, xxxiv. 488.

POWER, Mr. George, review of his treatise on the Egyptian ophthalmy, ix. 575.

PRADIER, M. account of his remedy for the gout, xxxiv. 430.

PRAGUE, the monk of, notice of his treatise on odours, vi. 339.

PRECOCITY, (physical, in human beings,) remarkable instances of, xxii. 243; xxiv. 389; xxvii. 522; xxxii. 145.

PREGNANCY, *uterine*, remarks on the diseases that accompany the state of, ix. 281; observations and reflections on the occurrence of sudden death during, xxvii. 181.—*See* FEMALES, FŒTUS, *and* GESTATION.

PREPARATIONS, *anatomical*, on the mode of preserving, vi. 274.

PRESCOTT, Mr. dissertation on the natural history and medical qualities of the *secale cornutum*, xxxii. 89.

PRETERNATURAL JOINTS, case treated by the seton, xxxiii. 507.

PRICE, Mr. Rees, observations on the conduct of the opposers of vaccination, xix. 426.

PRIESTLEY, Dr. account of his arguments in favour of the doctrine of phlogiston, i. 271; continuation of those arguments, and his objections to the new theory of the composition of water, iv. 266; vii. 380; his experiments with the voltanic pile, 381; his remarks on the new chemical doctrine, viii. 305, 307; ix. 273; on the conversion of iron into steel, xx. 347.

PRIMROSE, Mr. history of a case of mortification of the uterus, vi. 331.

PRIMULA, botanical description and medical qualities of, xii. 127.

PRING, Mr. history of a case of hernia cerebri, xxx. 74.

PRINGLE, Sir John, notice of his mode of treating tympanites, vii. 232.

PRIOR, Mr. remarks on pulmonary inflammation, xv. 56.

PRITCHARD, Dr. observations on epilepsy, xxxiv. 517.

PRIZE QUESTIONS, i. 205, 330, 399; v. 313, 558; vii. 300; viii. 280; ix. 91, 371; xii. 96; xiv. 381, 382; xvii. 198; xviii. 35; xxii. 260; xxiii. 352; xxiv. 261; xxv. 454, 542; xxvi. 76, 168, 345; xxxi. 517, 521; xxxii. 76, 521; for reports on the successful dissertations in reply to, *see* SOCIETIES.

PROCEDENTIA ANI ET UTERI.—*See* ANUS, *and* UTERUS.

PROCTOR, Mr. observations on the preparation of the mercurial ointment, i. 356; his mode of concentrating vinegar, ii. 109.

PROSTATE GLAND, remarks on induration of the, iv. 506; xxxiii. 159; account of the discovery of the middle lobe of the, xxviii. 6; remarks on that discovery, xxix. 226, 238; observations on the diseases of the, xxxi. 226.

PROUST, Dr. his experiments on the constituent parts of urine, vii. 474; observations on the gastric origin of insanity, xix. 94.

PROUT, Dr. his observations on the injecting of blood-vessels, xxx. 89.

PROVISIONS, account of a mode of preserving them by soda, ii. 383, 470; remarks on the mode of long preservations of, xxvii. 75.

PRUNELLA, botanical description and medical qualities of, xviii. 169.

PRUNUS, botanical description and medical qualities of, xvi. 76, 165.

PRURIGO, severe case of, xiv. 525.

PRUSSIC ACID, observations respecting the preparation of, x. 479; xxvii. 406; its poisonous qualities, xi. 93.

PSORA, observations respecting its nature, vii. 520; on the use of tobacco in the treatment of, xxvii. 45; account of a peculiar species of, xii. 194; on its origin from insects, xxxii. 496.—*See* ITCH.

PSYLLI, historical account of them, xxix. 304.

PTERIS, botanical description and medical qualities of, xix. 542.

PTERYGIUM, remarks on the treatment of, x. 563; observations on it, xvii. 80.

PTYALISM, on the use of diuretics in the treatment of, iii. 445; case of, induced by opium, iii. 586; its efficacy in curing consumption, viii. 308; (*see* CONSUMPTION;) observations on it, xxvii. 275; xxxiii. 454; on its good effects in the removal of disease, xxx. 37; case of, occurring without the use of mercury, xxvi. 452; remarks on that case, xxvii. 276.—*See* CALOMEL, *and* MERCURY.

PUBIS, observations on the division of the bones of the, during labour, ix. 57; xxiii. 498.

PUERPERAL CONVULSIONS.—*See* CONVULSIONS.

———— FEVER, abstract of the theories of, i. 381; remarks on the different opinions respecting it, 386; remarks on the cause of, ii. 387; on the utility of carbonate of potash in, iii. 80, 165, 264, 363; on the good effects of bark in, 442; description of the disease, viii. 433; variety of its symptoms, 434; appearances on dissection, 435; on its origin and supposed connexion with lacteal metastasis, 436; its difference from common inflammatory diseases, 438; remarks on the treatment of, and the necessity of caution in the use of stimulants, 442; the tincture of valerian and carbonate of potash beneficial in, 510; on the use of camphorated liniment in, 511; on the management of the breasts in, 512; cases of, 514; remarks on the alleged causes of, x. 11; different from milk-fever, 13; danger of bleeding in, 14; remarks on the different remedies, emetics, opium, diaphoretics, blisters, and bark, in, 15, 16, 17, 18; remarks on the inefficacy of the alkalies in, xii. 28; fatal case of, xxiv. 107; its appearance as an epidemic, xxv. 193; fatal case of, xxvi. 265; remarks on the character and treatment of, 269; on its supposed infectious nature, 272; remarks on, and especially on the necessity of early blood-letting, xxvii. 183; cases of, xxx. 515; remarks on, and on the efficacy of spirit of turpentine in its treatment, xxxii. 403; on its connexion with diarrhœa, 461; review of Dr. Armstrong's treatise on, 507; remarks on the use of spirit-of-turpentine in, xxxiii. 179; case of, with inflammation of the peritoneum, 447; remarks on the efficacy of spirit of turpentine in, xxxiv. 183; its fatality at Paris, 487.

PULLEY, Mr. history of a remarkable case of monstrosity, i. 224; some remarks on a case in which venesection was employed, ii. 265; observations on a case of monstrosity, iii. 138; case of abdominal dropsy, xii. 249; remarks on that case by Dr. Adams, who attributed it to hydatids, 552; case of amputation at the shoulder-joint, xiv. 108; observations on the cranium, 529; his analysis of James's powder, xv. 294; xxi. 438; xxv. 315; observations on a supposed case of vomiting of urine, xxx. 481; xxxi. 196.

PULMONARY AFFECTIONS, a strict limitation of the use of liquids, recommended in, by Dr. Lower, i. 20.—*See* LUNGS, *and* CONSUMPTION.

PULSATION in the abdomen, remarks on the causes of, xvii. 188; xxxi. 159.

———— of *Arteries.*—*See* ARTERY.

PULSE, review of Dr. Parry's treatise on the arterial, xxxiv. 163.—*See* ARTERY.

PUNCH, recommended in typhus as a substitute for wine, iii. 217.

PUPIL, (of the eye,) on the closure of, after the operation for cataract, x. 565; effects of the belladonna on the, iii. 367; iv. 5; on the dilitation of it, by belladonna, in operating for cataract, vi. 352; on the obliteration of it, and the use of belladonna to prevent that occurrence, xv. 55; on the formation of artificial, xvii. 403; xxv. 535; xxvii. 318; xxx. 192; xxxiii. 71, 192; on the influence of belladonna on it, xxviii. 258, 363; remarks on contraction and obliteration of the, xxix. 318; on the use of belladonna for preventing dilatation of it, xxx. 68, 511.

PURGATIVES, their utility in some cases of diarrhœa, i. 237; their utility in catarrhal fever, ix. 376; their utility in the cure of dysentery, xi. 442; remarks on the danger of violent, in children, xii. 108; their efficacy in chorea, xiii. 179; xv. 185; review of Dr. Hamilton's treatise on the use of, xv. 182; their utility in the treatment of fever, 183; especially when combined with emetics, xx. 165; their utility in diseases of the digestive organs, xvi. 272; remarks on the utility of, with bleeding, in chorea, xviii. 226; their utility in typhus when conjoined with bleeding, xxi. 169; remarks on their use in gout, 380; efficacy in dysentery, when combined with emetics, xxii. 429; in various local diseases arising from gastric disorder, xxiii. 242; their utility in dropsy, 335; xxvii. 340; effects of purging with elaterium in dropsy, xxiii. 335; xxxi. 144; their utility, especially elaterium, in hydrothorax, xxiii. 335; in gout, xxvi. 191; in dropsy, xxxi. 144; their great utility in scrofula, xxiv. 148; the progress of the use of in fever, in England, 430; their utility in the treatment of gout, xxvi. 190; on the use of scammony in gout, 191; on the efficacy of elaterium in the cure of that disease, 193; their general utility in that affection, 481; their good effects in the treatment of scarlatina anginosa, 442; cases of chorea cured by, xxvii. 421; further observations on their use in typhus, 458; vindication of their use in typhus, xxviii. 278, 289; authorities for the practice of purging in fever, 292; further remarks on their use in fever, 446; their great utility in the treatment of yellow fever, 447; xxxiii. 6; observations on their use in the treatment of petechiæ, xxx. 342; efficacy of strong purgatives in gout, 412; further remarks on the treatment of gout, xxxii. 199, 200, 201, 380; their efficacy in the treatment of epilepsy, xxxiii. 519; the good effect of powerful cathartics in epilepsy, xxxiv. 517.

PURPURA, (hæmorrhœa petechialis,) remarkable case of, i. 420; cases of, vi. 234; remarks on it, x. 444; and on the efficacy of the rhus radicans in,

480; observations on its nature and origin, xx. 280; diagnosis of, xxi. 321; on the utility of venesection in the treatment of, 146; history of a case of, xxix. 419; on the particular utility of purgatives in the treatment of, xxx. 342; fatal case of, 339.

PURSH, Mr. observations respecting the eupatorium perfoliatum, xxvii. 449.

PURTON, Mr. case of difficult parturition, iii. 44; observations on the croup, 240; on the physiology of the brain and nerves, iv. 334; case of secondary natural small-pox, v. 403; cases of paralysis of the bladder after parturition, and observations on the use of the catheter, vi. 1; reflections on the identity of the electric and nervous fluids, vii. 324; on the influenza of 1803, xi. 81; on the treatment of burns and scalds, xx.

PUS, experiments to determine the difference between it and mucus, iv. 49, 108, 203; observations and experiments relative to its formation, xxv. 326.

PUTREFACTION, observations and experiments on the means of preventing it in animal bodies, ix. 209; xi. 358; xvi. 35; xxxii. 435.

PYROLA UMBELLATA, observations on its diuretic qualities, xxxiii. 507.

PYROPHORUS, observations respecting the formation of it, xxxiv. 436.

PYRRHO, observations on apoplexy, vii. 515; Tyricola in reply to, viii. 64.

PYRUS, botanical description and medicinal qualities of, xvi. 169.

### Q.

QUACKERY, observations on the most effectual means of preventing it, i. 337; on the means of suppression of, ii. 150; remarks on, 274; remarks on the pernicious effects of, iii. 433; observations on, 523; iv. 16, 230, 404; vi. 129; strictures on, vii. 369; viii. 246; remarks on domestic, vii. 392; on the means of suppressing it, 461; xi. 554; remarks on the various forms of, xxxiii. 20.—*See* Charlatanism, *and* Medical Reform.

QUADRUPEDS, extract from a memoir on two species of non-descript, viii. 283.

QUARANTINE, oration on the laws of, delivered at Philadelphia in 1806, xviii. 111, 423, 497; remarks on the laws of, xix. 113, 198; xxi. 102, 296.

QUARRIER, Mr. observations on the poison of fish, xxv. 398.

QUEBEC, topography of, and account of the fever in the garrison there in 1805, xv. 446.

QUENSEL, Dr. his observations and experiments on galvanism applied to medicine, viii. 525.

QUERCUS, botanical description and medicinal qualities of, xv. 68.

QUICKENING, (relating to fœtal life,) an historical and critical dissertation on, xxiv. 38.

### R.

RABIES, *canina*, observations respecting its spontaneous origin in dogs, xiii. 478; remarks on its nature, origin, and propagation, xxvi. 437; a general dissertation on, xxvii. 236; observations on its nature and origin, xxxi. 231; review of a treatise on, xxxiii. 315; remarks on the same treatise, 293.—*See* Hydrophobia.

RACHITIS, remarks on the cause of, vi. 191; xiv. 375.

RADDLESDEN, Mr. observations on the influenza of 1803, x. 213.

RADESYGE, account of the nature and opinions entertained respecting the origin of that disease, xxii. 432.

RADFEARN, Dr. history of a case of diabetes mellitus successfully treated, i. 219; observations on vaccination, ii. 23; on the influenza of 1803, x. 211.

RAMOND, history of the *cagots* of the Pyrenees, xxxiii. 219; remarks on his history, by Dr. Adams, 433.

RAMSAY, Dr. David, of Charleston, U. S. case of the bite of a snake cured by volatile alkali, xi. 332; remarks on that case, by Dr. Barton, 334; review of his Charleston Medical Register, x. 468.

———, Dr. Alexander, description of a disease incidental to the lymphatics, xiv. 50; composition of a new species of anatomical injection, ibid.; case of mercurial erythema, xxvi. 341; observations respecting his plates of the brain, xxviii. 11; account of some unusual conformation of muscles, 505; observations on sexual diseases, xxxi. 210; review of his anatomical treatise, 249; observations on muscular motion, 288, 458.

———, Mr. Alexander, case of erythema mercuriale, xx. 341.

RAMSBOTHAM, Dr. observations on puerperal fever, xxvi. 265; case of sudden death without obvious cause, in the last month of pregnancy, xxvii. 179; case of rupture of the uterus during labour, xxx. 316; case of extra uterine fœtation, 318; reflections on the occurrence of sudden death after parturition, xxxii. 135; case of rupture of the uterus during labour, 194.

RAMSDEN, Mr. observations on a membranous septum at the orifice of the urethra, xxv. 118; review of his work on diseases of the testicle, 175, 265, 350.

RANCE, Mr. case of fungus hæmatodes of the kidneys, xxxii. 19.

RAND, Mr. on the use of calomel in yellow fever, i. 79.

RANULA, remarks on its treatment, vii. 292.

RANUNCULUS, botanical description and medicinal qualities of, xvii. 372.

RAPHANUS, botanical description and medicinal qualities of, xviii. 369.

RASORI, account of his new theory of the agency of the materia medica, xiv. 572.

RASPATORY, description of an improved, viii. 485.

RATAULICA, botanical description and medical qualities of, xiv. 127; observations on its medicinal utility and effects, xxii. 107.

RAYMOND, Mr. observations on the danger consequent on the cure of some diseases, xxiv. 121, 214.

READE, Dr. Joseph, account of his experiments to shew the power of friction in the production of heat, xx. 97; observations on epiphora, xxvi. 227.

RECTUM, case of disease of, and remarks on induration of the, iv. 506; case of invagination of the colon in the, xi. 115; case of cancer of the, xiii. 306; observations on schirrus of the, xxii. 281; observations on the diseases of the, xxiv. 415; observations on stricture of the, xxvii. 413; xxxi. 403; observations on a carcinomatous affection of the, xxxii. 246; review of Mr. Copeland's work on the diseases of the, xxxiii. 236; case on unusual disease of the, xxxi. 225.

REECE, Mr. of Cardiff, observations on the influenza of 1803, x. 229.

———, Mr. of Chepstow, notice of his Medical and Surgical Pharmacopœia, vi. 88.

———, Dr. Richard, observations on extraction of the teeth, with the description of a new instrument, vii. 229; notice of his new preparation of Peruvian bark, 473; notice of his domestic Medical Guide, ix. 485; observations on the anti-phthisical qualities of the lichen islandicus, xi. 90.

REED, Dr. case of hydrocephalus, xxiv. 516.

REES, Dr. George, review of his treatise on disorders of the stomach, xxiv. 237; remarks on that work, by Dr. Bree, 357.

REEVE, Mr. history of a case of hydrocephalus terminating favourably, iii. 61; remarks on aneurism, 531; historical sketch of the use of cold affusion in fever, iv. 397.

———, Dr. history of Cretinism, xiv. 82; remarks on his work on Cretinism, xxii. 9; observations on chorea, xxix. 64.

REGIMEN, review of Dr. Willich's lecture on, ii. 301, 393, 493; account of that recommended by Professor Tode, of Copenhagen, in hypochondriasis, &c. i. 150.—*See* Diet.

REGISTER, the Charlestown Medical, review of, x. 468.

REGNAULT, Dr. J. B. review of his observations on pulmonary consumption, and on the efficacy of lichen islandicus in the treatment of that disease, viii. 463; notice of the French translation of that work, 564; an extract from his observations on pulmonary consumption, xi. 283; further observations on the medicinal qualities of lichen islandicus, xii. 463.

REICH, Professor, review of his treatise on the theory and practice of fever, iv. 556; v. 178; vi. 86.

REID, Mr. observations on Mr. Pott's remarks on the artificial leg, vi. 547; review of his treatise on ophthalmia, xvii. 385.

———, Dr. Peter, observations on the study of medicine, xxvi. 68.

———, Dr. John, extract from the notes subjoined to the French translation of his treatise on consumption, ii. 50; reflections on the relative disposition of the different classes of society to disease, iii. 391; remarks on the character of the epidemic diseases of London, and on the proportion of the fatality of fever in the year 1800, 393; observations on the efficacy of emetics in cutting short fever, when exhibited in its first stage, ibid.; observations on the treatment of fever, ibid. and 394; case of remarkable periodical paralysis in a girl, 395; observations on the utility of active exercise in the cure of amenorrhœa, 409; on the treatment of lumbago in women, 410; on the origin and nature of the diseases of the poor in London, ibid.; remarks on the medical treatment of General Washington in his last illness, 473; on the origin and character of pulmonary diseases, 506; remarks on the influence of the imagination as a means of cure of disease, 507; on the character of the diseases particularly prevalent in different artisans, &c. 508; observations on the origin and character of disorders of the stomach, iv. 15; case of extraordinary affection from the influence of music, in a girl, ibid.; on the medical use of electricity in amenorrhœa, ibid.; remarks on the use of violent stimulants in chronic diseases, 16; extraordinary case of catalepsy, ibid.; observations respecting the contagious nature of typhus fever, 106; on the disposition of females to mania at certain periods of life, ibid.; on the treatment of dyspepsia and hypochondriasis, ibid.; observations on typhus fever, 203; on the seasons in which cholera and diarrhœa are respectively prevalent, 298; on the origin and treatment of cholera, 299; on the character and treatment of diarrhœa, 394; remarkable case of ptyalism occurring without the use of mercury, ibid.; observations on typhus fever, 395; remarks on the propagation of typhus fever by contagion, ibid.; on the origin and character of autumnal pulmonary diseases, 490; on the atrophia of infants, 492; on the prevalence of typhus fever, and its connexion with want of a sufficiency of food, v. 97; on the necessity of hospitals for the sick poor of the metropolis, 98; on cerebral affection in typhous fever, 194; observations on the state of the poor of the metropolis, 195; case of melancholia occurring subsequently to the loss of sight, ibid.; case of pulsation in the abdomen mistaken for aneurism, ibid.; on the effects of moral influence on disease, 196; on the state of the air respired in the metropolis, and its influence on the animal economy, ibid.; on the utility of stimulants in the treatment of typhus fever in the poor, ibid. and 197; on cerebral affection in fever, 301; on the nature and treatment of amenorrhœa, 338; on catarrhal and asthmatic affections, 339; on the origin of hypochondriasis, ibid.; on the origin and nature of scrofulous affections, especially on their connexion with a low temperature of the atmosphere, 413; on the treatment of that disease, 414; case of trismus traumatica, x. 345; observations and cases demonstrative of the efficacy of cold ablution in scarlatina, xi. 27; observations on the asthenic character of the typhus of the existing period, compared with the inflammatory nature of that of former ages, especially at the era of Sydenham, xiii. 376; remarks on the progress and treatment of phthisis, 377; reflections on the state of parts accompanied with pain, 476; on the agency of blisters, ibid.; observations on what are termed nervous diseases, ibid.; on the efficacy of hyoscyamus in the cure of those diseases, 477; on pulmonary disorders mistaken for phthisis, 568; his opinion that no person *ought* to die of fever, ibid.; on the merits of the doctrines of Brown, 569; explanation of his proposition that no person *ought* to die of fever, xiv. 14; case of amaurosis induced by terror, 15; case of paralysis induced by grief, 16; on the disposition of women to melancholia at certain periods of life, ibid.; on the improper use of bleeding commonly adopted, 183; on the utility of cold affusion in scrofulous ophthalmia, 184; on the state of the atmosphere of the metropolis, ibid.; on the influence of the passions on the body, 275; on the diseases of the poor, 276; on the relative importance of mental and bodily exercise, 277; case of apoplexy consequent on inordinate bodily and mental exertion, which terminated fatally, 379; remarks on the use of bleeding in that affection, ibid.; case of fatal apoplexy, treated by bleeding, in a female, consequent on the application of cold to the feet about the menstrual period, ibid.; general remarks on the treatment of apoplexy, 380; on the use of purgative glysters in some cases of intestinal inflammation, accompanied with debility, 476; case of dyspepsia, accompanied with particular fœtor of the breath, 477; reflections on the importance of attending to moral influence in the treatment of disease, 478; on the influence of the atmosphere of the metropolis on the human body, 569; on the utility of cold ablution in the treatment of scarlatina, 570; on the origin and character of dropsical affections, ibid.; observations on the disease vulgarly termed a *cold*, xv. 91; considers that of Brown, which attributes it to heat when the body is cold, as the true doctrine of the origin of that affection, ibid.; considers most of the inflammatory diseases of the present day as of asthenic origin, 194; on local and constitutional disorders, ibid.; on the use of the Portland powder in gout, ibid.; on the importance of an healthy state of the stomach to the welfare of the system, 195; on nervous affection, ibid.; on dyspepsia, 291; on the importance of due mastification of the food, 292; on the importance of activity of the body and mind to the health of the system, 293; review of his treatise on consumption, 460; remarks on that work, xviii. 10; remarks on scrofula, xxv. 178; case of epilepsy, attended with some remarkable premonitory sensations, 179.—*See* DISEASES, REPORTS OF, *in the Finsbury Dispensary.*

REIMANUS, Dr. observations on the utility of the application of belladonna to the eyes, previous to the operation for the cataract, iii. 367.

RELIGION, remarks on that of physicians, xxxii. 30.

REMEDIES, remarks on the necessity and advantage of gradually augmenting the dose of active ones, in the treatment of obstinate chronic diseases, iv. 160.—*See* MATERIA MEDICA.

REMEMBRANCER, on the mode of obtaining metallic casts from the osseous cavities of the ear, x. 171; reply to, by Mr. Low, 357.

RENAUD, M. observations respecting arsenic in its metallic state, xi. 287.

RENAULT, M. observations on a case of strangulated hernia, x. 248.

REPREHENSOR, observations on small-pox, xxv. 490.

RESEDA, botanical description and medicinal qualities of, xxv. 74.

RESIN, *animal*, bezoardic, history, physical character, and chemical properties of, ix. 498.

RESPIRATION, enquiry respecting the causes of disordered, iv. 260; physiological observations on, 274; enquiry respecting its dependance on volition, vi. 372; Mr. Ellis's theory of, xix. 371; remarks on that theory, by Dr. Bostock, 527; experiments shewing its relation to the influence of the eighth pair of nerves, xxvi. 98; remarks on Dr. Bostock's strictures on Mr. Ellis's treatise on, 181; observations and experiments respecting its connexion with the development of heat, xxvii. 9; experiments relative to the chemical changes with which it is accompanied, xxxii. 132; observations of M. Becland, tending to show that the fœtus breathes the liquor amnii, 521; remarks on Dr. Priestley's theory of, xxxiii. 82.

RESUSCITATION, observations on the act of, by Dr. Struve, ii. 497; instance of, after supposed death from yellow fever, xiv. 8.

REVIEWING, remarks on, xxii. 441.

REWELL, Mr. observations on the influenza of 1803, x. 409.

REYNAUD, M. case of abscess of the liver consequent on injury of the head, xxxii. 119.

REYSS, Dr. his letter to Dr. Jenner on the introduction of vaccination into Poland, xxvi. 39.

RHAMNUS, botanical description and medicinal qualities of, xii. 221.

RHEUMATISM, remarks on the most effectual treatment of, i. 384; on the efficacy of acetic æther in, used by friction, iv. 369; review of a treatise on the nature, cause, prevention, and cure, of, v. 289; comparative view of the theories of Cullen, Brown, and Darwin, respecting it, 388, 577; observations on the efficacy of the air-pump vapour-bath in the treatment of, 59; on the efficacy of galvanism in the treatment of some cases of, viii. 257; on the mode of applying galvanism in the treatment of rheumatism, ix. 135; efficacy of cold-bathing in a violent case of, 549; remarks on, and on the efficacy of diminished temperature in its cure, x. 256, 357; observations respecting the cure of, xi. 492; a severe case of, cured by arsenic, xiii. 16; further observations on the efficacy of arsenic in the cure of, 525; case of, successfully treated by opium, xiv. 81; remarks on its characters, 118; observations respecting the treatment of, by cinchona, 323; case of acute, terminating in paralysis, xv. 175; general observations on its nature and treatment, ibid.; observations on the nature and treatment of, xviii. 275; case in which electricity was beneficial, 428; general observations on, xix. 368; observations on the utility of arsenic in chronic, 369, 467; on the use of cinchona in acute, 427; on the utility of the oil of the Chinese radish in the treatment of, 479; on the utility of ginger in, 527; various reflections on its nature and treatment, xx. 246, 356; xxi. 518; xxii. 267, 348; on the good effects of calomel and opium in the treatment of, xxvi. 210; cases and remarks respecting the efficacy of mercury in the treatment of, 277; observations on its nature, 495; on the good effects of opium and cinchona in, 496; observations respecting the utility of opium in the cure of, xxvii. 289; on the utility of exciting perspiration by muscular action in, xxx. 235; observations on its treatment by cinchona, xxxi. 89; on the use of low temperature in the treatment of, 269; various observations on, 363; case of, affecting the heart by metastasis, xxxii. 230; observations on the remarkable efficacy of percussion and compression in the cure of, xxxiv. 196.

RHODIOLA, botanical description and medicinal qualities of, xv. 70.

RHODIUM, discovery, and mode of obtaining the oil of, xiii. 90.

RHODODENDRON, *chrysanthum*, supposed to be the eanche, xxv. 183.

RHUS, *radicans*, (the *rhododendron* of TOURNEFORT,) its utility in palsies and in herpetic diseases, vi. 272; x. 480.

———, *vernix*, account of some experiments on, i. 108; observations on its medicinal qualities, ibid.

RIBES, botanical description and medicinal qualities of, xii. 221.

RICA, M. observations on the influenza of 1803, x. 112.

RICARDS, Mr. on the use of electricity in blindness, with an account of the successful treatment of a case of blindness from effusion of blood into the chambers of the eye, v. 68; case of hydrocephalus successfully treated, 341; observations on cancerous affections, vi. 509.

RICHARD, M. account of his analysis of the broth prepared from bones, xii. 353.

RICHERAND, Professor, review of his elements of physiology, vii. 469; his arrangement of Boyer's treatise on the diseases of the bones, xix. 79; observations on gun-shot wounds, xxx. 377, 458.

RICHMOND, Mr. account of his improvement of the speculum oculi, iv. 103; observations on ischuria and on hernia, 331; observations on the case of Anne Moore, xxix. 469.

RICHTER, Dr. of Gottingen, observations on the hydrops vagus, ii. 182; remarks on the test for arsenic, iii. 269; account of his method of obtaining tungsten, x. 479; his discovery of niccolanium, xv. 390.

RIEG, Dr. description of his new method of operating for the hare-lip, xi. 195.

RIGBY, Dr. E. remarks on the improbability of measles being influenced by vaccination, xxxi. 273; observations on vaccination, xxxii. 107.

RING, Mr. remarks on vaccine inoculation, ii. 23; observations on venesection, 346; observations respecting a case of catalepsey, iii. 402; on the utility of burned sponge in bronchocele, and observations on the Coventry remedy for that disease, iv. 412; further remarks on bronchocele, and on an instance of the fatal effects of a large dose of Dalby's Carminative, v. 38; observations on cow-pox, and in favour of its permanent prophylactic influence, 281; further observations on bronchocele, 354; on the establishment of vaccine inoculation in the East and West Indies, in Sicily, and in Russia, 543; on the anodyne quality of camphor, vi. 155; on vaccine inoculation, 486; on the progress of vaccination in different parts of the world, vii. 109; on the treatment of ranula, itch, and tinea capitis, vii. 292; review of his treatise on cow-pox, 168; his evidence in favour of vaccination before a committee of the House of Commons, 492; observations on anti-psoric preparations, viii. 43; remarks on Mr. Pear's cases of vaccine inoculation, 198; on the progress of vaccination in different parts of the earth, ix. 64; remarks on vaccine innoculation, 296; x. 159; history of a case of dolor faciei, 420; remarks on vaccination, 421; remarks on his treatise on cow-pox, 473; his remarks on a letter addressed to him from Jamaica on cow-pox, 545; extract of a letter from Dr. Marshall, with observations, xi. 39; observations on the pustulous eruptions of animals, 431; further observations on the use of burned sponge in bronchocele, xii. 9; on diarrhœa and cholera morbus, 102; reply to Mr. Goldson in favour of cow-pox, 181; observations relative to the exposure of patients to small-pox infection after vaccination, 183; observations on the variolous pustule occurring after vaccination, 356; on the mode of preserving leeches, 357; on pustular eruptions after vaccination, 358; remarks on a supposed case of small-pox after cow-pox, 446; remarks on Dr. De Carro's work on the introduction of vaccination into Turkey, Greece, and the East Indies, 447; on a reported case of small-pox after vaccination, 429; remarks on Dr. Rollo's cases, 530; remarks on Dr. Mc Donald's answer to Mr. Goldson, 534; case of co-existence of chicken-pox with scarlatina 537; observations on the blue vaccine vesicle, xiii. 147; case of malformation of the heart, 120; remarks on Dr. Walker's reply to Dr. Adams, 168; remarks on inoculation, 245; remarks on Mr. Shoolbred's report, 338; observations on inoculation, and on the preservation of vaccine virus, 345; on the state of vaccination in France, 419; observations on Mr. Goldson's account of vaccination, 457; remarks on vaccination, 569; remarks on a reported case of small-pox after cow-pox, xiv. 6; case of severe chicken-pox, 141; observations on vaccination, and on cow-pox, 192; observations on Dr. Adams's paper on cow-pox, (p. 193.) 355, 401, 509; further observations on vaccination, xv. 56; remarks on Dr. Rowley's pamphlet, 157; address to Dr. Mosely, 191, 291; on vaccination in America, 311; cases of secondary small-pox, 432; observations on vaccination, 516; case of angina pectoris, xvii. 9; observations respecting the origin of cow-pox, 11; history of several cases of secondary small-pox; remarks on some opinions respecting vaccination given in the Edinburgh Review, 141; observations on cow-pox, 233; case of ticdouloureux cured by cicuta, xviii. 135; observations on cow-pox, 314; replies to the statements of Mr. Birch and Dr. Mosely, 386; reply to Mr. Davies, xix. 53; observations respecting the use of cathartics in gout, xxi. 380; review of his treatise on gout, 478; observations on the effects of the Eau Medicinale, xxv. 204; on various topical remedies, 497; remarks on the supposed identity of cow-pox with the grease of horses, xxvi. 122; various remarks respecting vaccination, 23, 239, 315, 441, 539.

———, Mr. J. case of wound of the femoral artery, xxvi. 14.

ROBERTSON, Dr. A. notice of his Colloquia Anatomica, xix. 178.

————, Mr. Archibald, case of poisoning by oxy-muriate of mercury, xxviii. 223.

ROBERTON, Dr. John, remarks on the internal use of cantharides in gleet and leucorrhœa, xv. 415; xvii. 279; observations on some diseases of horses, xviii. 555; remarks on medical police, 558; report of the diseases of Edinburgh, in the year 1807, 565; reports of the diseases of Edinburgh in the year 1808, xix. 182, 273, 361, 470, 569; xx. 89, 190, 283, 378, 477, 561; plan of medical police, xix. 237; cases illustrative of the efficacy of cantharides in various species of ulcers, xx. 290, 429; observations on the principal diseases of the female organs of generation, xxi. 29; reports of the diseases of Edinburgh in 1809, xxi. 82, 258, 332, 431, 517; observations on stricture of the urethra, 303, 371; further remarks on strictures, xxii. 17, 126; continuation of the report of the diseases of Edinburgh for 1809, 84, 169, 257, 346, 433, 522; observations on hydrocephalus internus, 378; report of the diseases of Edinburgh in 1810, xxiii. 81, 170, 237, 347, 433, 521; further remarks on the use of cantharides in various diseases, 311; continuation of the report of the diseases of Edinburgh in 1810, xxiv. 83, 172, 252; reply to some remarks on his opinions respecting hydrocephalus, 210; further remarks on the internal use of cantharides in gleets and leucorrhœa, xxvi. 78; review of his treatise on the diseases of the generative system, xxvii. 139; observations on pneumonia, xxxii. 67, 358; cases of pneumonia, xxxiii. 171, 283; observation on ptyalism, 454.

————, Mr. W. case of suppuration of the omentum, x. 29; case of hernia congenita, 33; observations on an enlargement of the knee-joint, xxii. 515.

ROBERTSON, Dr. H. review of his view of the natural history of the atmosphere, xx. 365.

ROBINSON, Dr. of Newcastle, observations on the influenza of 1803, x. 109.

————, Dr. of Derby, history of a case of abscess of the liver, x. 489; case of compound fracture of the humeurs, xiv. 267.

ROBIQUET, M. analysis of cantharides, xxvii. 126.

ROCHE, M. cases of diseased bladder, xi. 61; of divided larynx, 12.

RODMAN, Dr. observations on the influenza of 1803. x. 46; case of tumor of the eyes, xi. 197; cases of aneurism of the aorta, and of imperforature of the anus and vagina, xii. 335; case of large calculus removed by breaking the stone in the bladder, xxx. 245; review of his treatise on cancer in the female breast, xxxiv. 156; case of a child born between the fourth and fifth months being reared alive, 517.

ROGERS, Dr. observations in favour of the external bark of the mezereum to produce issues, v. 278; on the efficacy of laurel-water in restraining hæmorrhages, v. 500; on the use of cold water and ice in typhous fever, vi. 106.

————, Mr. W. H. of Gravesend, histories of two cases of phthisis successfully treated by digitalis, viii. 82; review of his work on cow-pox and small-pox, xiii. 468.

————, Mr. of Maningtree, history of a case in which cow-pox inoculation superseded the agency of the contagion of small-pox, ix. 544.

ROGET, Dr. history of a case in which a large dose of arsenic was taken that terminated favourably, xxviii, 64; observations on the tests for arsenic, 187, 469,

ROLFE, Mr. histories of eighteen cases of dropsy unsuccessfully treated by digitalis, v. 406; on the efficacy of yeast in typhous fever, 508; case of secondary small-pox in a woman, on which occasion it was communicated to her infant, vi. 3. further observations on the efficacy of yeast in typhous fever, vi. 112, 196; observations on the influenza of 1803, x. 98; observations on superfœtation, xxiv. 234.

ROLLO, Dr. observations on diabetes mellitus, i. 102; remarks by Fourcroy on his chemical treatment of that disease, 195; observations on the means of destroying the virus of ulcers and contagious miasmata, 429; account of the royal artillery hospital at Woolwich, with observations on the management of the soldiery, and the preservation of their health, v. 383; remarks on re-inoculation with vaccine and variolous matter, xii. 203; observations on sulphurous mineral waters, xx. 136.

ROOSE, Professor, observations on the corpora lutea in the ovaria of females, v. 587; remarks on the power of volition in respiration, vi. 372.

ROSA, botanical description and medical qualities of, xxvi. 261.

ROSANSHŒLD, observations on the lues bovilla of Sweden, xxiii. 431.

ROSEOLA, observations on, xx. 278.

ROSES, on the mode of forming the essential oil of, into crystals, xi. 180.

ROSSI, Professor, account of his experiments on artificial impregnation of animals, i. 47.

ROTTERDAM, observations on the practice of vaccination at, vii. 251.

ROUSSEAU, John James, his opinion cited on the advantages resulting from the study of nature, i. 73.

——————, Dr. experiments on cutaneous absorption, xv. 274; case of resuscitation of a still-born child, xviii. 307.

ROUTIER, Dr. notice of his work on puerperal fever, xi. 383.

ROUX, Dr. case of invagination of the colon in the rectum, xi. 115.

ROWE, Mr. remarks on vaccination, xxxiii. 274; case of recovery from the effect of a large dose of opium, 201.

ROWLANDS, Mr. account of two successful cases of lithotomy, ii. 287; remarks on an uncommon occurrence after amputation, iii. 4; on the evolution of the fœtus, 5; observations on hydrocele, viii. 34.

ROWLEY, Dr. remarks on the treatment of syphilis, iii. 480; review of his Schola Medicinæ, xii. 469, 564; review of his treatise on cow-pox, xiv. 565.

ROYAL SOCIETY, of *London*, abstract of the history of the xxviii. 314. —*See* SOCIETIES.

ROYSTON, Mr. cases of secondary small-pox, xv. 442; observations on the advantages that would result from the construction of a Bibliographia Medicinæ Brittanicæ, xvii. 392; his historical sketch of the progress of medicine, in the year 1806, xviii. 1.; the same for the year 1807, xx. 1; for the year 1808, xxii. 105; observations on an acute eruptive disease of the penis, xxiii. 441; historical sketch of the progress of medicine in 1809, xxiv. 1.

RUBIA, botanical description and medical qualities of, xii. 28; its efficacy as an emenagogue, xxvii. 105.

RUBUS, botanical description and medical qualities of, xxvi. 262.

RUBY, analysis of the, iii. 173.

RUDOLPHI, Professor, notice of his Annals of Medicine and Natural History, ii. 399, 498.

RUIZ, Don Hypolito, account of the plant ratanhia, xiv. 127.

RULE, Mr. remarks on the means of preventing bilious fevers, vi. 310.

RUMFORD, Count, account of his doctrine respecting heat, xxvii. 510.

RUMSEY, M. observations on the croup as it appeared in the years 1793 and 1794, at Chesham, iv. 67; case of enteritis, with remarks on that disease, xiii. 182.

RUMEX, botanical description and medical qualities of, xiv. 70.

RUSH, Dr. Benjamin, account of his treatment of yellow fever by producing salivation, i. 79; notice of the fifth volume of his Medical Inquiries and Observations, 105; observations on the nature and cure of hydrophobia, ii. 173; his observations and opinions on the beneficial effects of depletion in strangers who have to encounter a baneful climate, 178; his remarks on the nature and cure of gout, 376; account of his lectures on animal life, iii. 184, 283; his remarks on the nature and origin of the yellow fever at Philadelphia, v. 291; account of a case of resuscitation from supposed death, xiv. 7; observations on the efficacy of acetate of lead in epilepsy, xiv. 10; account of the change in his opinion as to the contagious nature of yellow fever, xv. 250; cases of consumption, and remarks on the utility of mercury in the treatment of that disease, xix. 47, 506; reflections on the means of lessening the pain and danger of child-bearing, xx. 73; his remarks on Dr. Physick's proposal to open the trachea in hydrophobia, 358; reflections on the nature of the functions of the spleen, liver, pancreas, and thyroid gland, xxvi. 193.

RUSPINI, Il Cavalliero, description of his instrument for extracting balls from gun-shot wounds, xv. 455.

RUSSEL, Mr. W. notice of his Medical Queries and Investigation, viii. 380.

———, Dr. history of two cases of the existence of small-pox and measles in the same person, iv. 67.

RUTHERFORD, Mr. history of the appearances on dissection in a case of diabetes, xiv. 268.

RUTTER, Dr. observations on the influenza of 1803, x. 519; case of hysteralgia, xix. 555; history of a case of erythema, xxi. 417.

RYAL, Dr. observations respecting the use of cold affusion in colic, xiii. 9.

RYAN, Dr. observations on the medicinal qualities of acetate of lead, vii. 507; further observations on the internal use of lead, viii. 509; history of a case of apoplexy, 210; remarks on the cure of autumnal fevers, ix. 213; reflections on contagion, 217; observations on the influenza of 1803, x. 296; account of an epidemic that prevailed at Kilkenny in the year 1800, 296; continuation of the same subject, 305.

SALSOLA, botanical description and medical qualities of, xii. 229.

